# UHRF1 overexpression generates distinct senescent states with different Tp53 dependencies

**DOI:** 10.1038/s44319-026-00941-y

**Published:** 2026-09-28

**Authors:** Elena Magnani, Charlene Chen, Filippo Macchi, Tijana Randic, Bhavani P Madakashira, Ian McBain, Kirsten C Sadler

**Affiliations:** 1 Program in Biology, NYU Abu Dhabi, PO Box 129188, Abu Dhabi, UAE; 2 Center for Genomics and Systems Biology, NYU Abu Dhabi, Abu Dhabi, UAE

**Keywords:** Autophagy & Cell Death, Cancer, Cell Cycle

## Abstract

Senescence is a pleiotropic phenotype that alternatively suppresses or promotes cancer. Tumor-suppressive roles are attributed to irreversible cell cycle arrest and immune clearance, while tumor-promoting functions include apoptosis resistance and immune evasion, allowing cells to persist and support the tumor microenvironment or escape senescence and proliferate. Epigenetic changes are common features of senescent cells, and we used a preneoplastic liver cancer model caused by overexpression of the epigenetic regulator, UHRF1, in zebrafish hepatocytes to investigate how epigenome repatterning generates diverse senescent phenotypes. Early responses to UHRF1 overexpression include DNA damage, DNA methylome repatterning, retrotransposon derepression, cell cycle withdrawal, and *atm* and *tp53*-dependent senescence. scRNAseq analysis uncovers distinct senescent cell populations, some expressing immunogenic and anti-apoptotic genes, and others expressing proliferation genes. These populations have different capacities and susceptibilities based on UHRF1 levels. Cells with low UHRF1 levels proliferate in *tp53* mutants, while cells with high UHRF1 levels do not re-enter the cell cycle and are targeted by the senolytic Navitoclax. This indicates that UHRF1 expression level can generate diverse senescent cell phenotypes with divergent outcomes.

## Synopsis

UHRF1 overexpression in zebrafish hepatocytes induces heterogeneous senescent phenotypes that persist, escape, or undergo senolytic elimination.

- UHRF1 overexpression triggers DNA damage and activates Atm-Tp53 pathway.
- Distinct levels of UHRF1 generate different senescent phenotypes.
- Following loss of Tp53, hepatocytes with low or mid UHRF1 levels resume proliferation.
- Hepatocytes with high UHRF1 levels remain arrested despite Tp53 loss and are selectively eliminated by Navitoclax.

## Abstract (web summary)

UHRF1 overexpression in zebrafish hepatocytes induces heterogeneous senescent phenotypes that persist, escape, or undergo senolytic elimination.

## Graphical abstract

**Figure:**
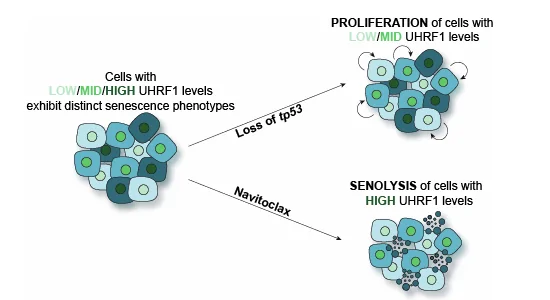

## Introduction

Tumor suppression is achieved through the coordination of cell death, senescence, and immune clearance of pre-cancerous cells. Cancer arises when damaged cells evade these safeguards, acquiring the ability to exploit resources, withstand stress imposed by the microenvironment, and proliferate. Senescence was originally conceived as a homogenous cellular state characterized by irreversible cell cycle arrest resulting from replicative exhaustion, aging, genotoxic stress, or other extreme forms of irreparable damage. It is now clear that senescence is a much more complex phenotype (Avelar et al, 2020; Chan and Narita, 2019; Hsu et al, 2019; Kwiatkowska et al, 2023; Ogrodnik et al, 2024). Some senescent cells can simultaneously activate tumor-suppressive processes, such as cell cycle arrest, as well as tumor-promoting features (Hsu et al, 2019), which reinforce the tumor ecosystem, promote mechanisms to either outcompete their neighbors for resources or kill them off (Vishwakarma and Piddini, 2020), and to survive in conditions that are suboptimal for non-transformed cells (Chan et al, 2024; Ciriello et al, 2024; Hausser and Alon, 2020; Loukas et al, 2023). An understanding of senescence pleiotropy and of the features conferring tumor-suppressive or tumor-promoting activities to senescent cells can enable manipulation of this complex phenotype as a cancer therapy.

While all senescent cells have some features in common, including cell cycle withdrawal and phenotypic changes, their identity and fate vary depending on the senescence-inducing stimuli, cellular context, and duration of senescence (Campisi and d’Adda di Fagagna, 2007; Chan and Narita, 2019; Hsu et al, 2019; Huna et al, 2024; Ogrodnik et al, 2024). Defining different phenotypes of senescent cells—i.e., senotypes—has proven challenging, as there is no single marker to distinguish them (Anerillas et al, 2026; Casella et al, 2019; Fernandez et al, 2025; Ogrodnik et al, 2024). DNA damage is a common senescent trigger, and a persistent DNA damage response (DDR) with activation of Tp53, Cdkn1a (p21) and other downstream targets that block proliferation is a feature of most senotypes (Di Micco et al, 2011; Fumagalli et al, 2014; Ogrodnik et al, 2024), with the dynamics of Cdkn1a expression dictating whether cells will persist or proliferate (Hsu et al, 2019). Other defining features are irreversible cell cycle arrest, increased senescence-associated beta-galactosidase (SA-β-gal) staining, activation of a pro-inflammatory senescence-associated secretory phenotype (SASP), which induces an inflammatory response and senescence-associated chromatin alterations, including senescence-associated heterochromatin foci (SAHF) and DNA methylation changes. Not all senescent cells display all these features, reflecting the pleiotropy of senotypes (Fernandez et al, 2025; O’Sullivan et al, 2024; Ogrodnik et al, 2024).

This diversity in senotypes translates to distinct cancer-related fates. Some senescent cells are immunogenic, cleared by the immune system, and therefore do not contribute directly to cancer. Others have an opposing phenotype and become resistant to immune clearance and to apoptosis, persisting even as neighboring cells are cleared (Chan et al, 2024; Colucci et al, 2025; Kang et al, 2011). Persistent senescent cells are dangerous, as some acquire a phenotype that retains the capacity to proliferate when Tp53 or other cell cycle inhibitors fluctuate or are inactivated, allowing these damaged cells to escape, transform, and contribute to cancer (Hsu et al, 2019; O’Sullivan et al, 2024; Reyes et al, 2018). Other senescent cells, such as those in deep senescence (De Cecco et al, 2019; Goy et al, 2023; Gurkar et al, 2025), are incapable of proliferating but are still dangerous, as they support the tumor microenvironment by stimulating angiogenesis or by directly supporting neighboring cancer cells with mitogens, pro-survival signals, or factors promoting epithelial-to-mesenchymal transition (Colucci et al, 2025; Huna et al, 2024).

Senescent cell diversity is directly relevant to clinical applications, as approaches to selectively reinforce tumor-suppressive senescence while eliminating pro-tumorigenic senescent cells require methods to identify these different identities. An example is provided by senolytics, such as Navitoclax, which dismantle proteins and pathways that render senescent cells resistant to apoptosis (Colucci et al, 2025; Zhu et al, 2016). However, Navitoclax is not effective on all senescent cell phenotypes. This could underlie the finding of minimal efficacy of Navitoclax in lung cancer (Rudin et al, 2012) and the failed clinical trial for advanced hepatocellular carcinoma (HCC) using a pro-drug (Emiloju et al, 2024), despite showing promise in a mouse model of HCC (Yang et al, 2025). This suggests that Navitoclax is ineffective in removing tumor-promoting senescent cells. Thus, defining the cellular and molecular characteristics of diverse senotypes is essential to develop therapies to target the senescent cell populations that support cancer development.

To understand the fate of diverse senotypes, it is important to understand how they are generated. Epigenetic modifications are a primary mechanism dictating cell identity, and epigenetic changes occur both as a consequence and as a cause of senescence (Cruickshanks et al, 2013; Di Micco et al, 2011; Hernandez-Segura et al, 2018; Ogrodnik et al, 2024; Parry et al, 2018). Therefore, epigenetic changes present a possible mechanism of phenotypic diversification of senescent cells. The widespread loss of DNA methylation on transposable elements (TEs) and the hypermethylation of some promoters are hallmark features of cancer (Hansen et al, 2011; Lister et al, 2009), which are already present in senescent cells (Cruickshanks et al, 2013; Hlady et al, 2017). The finding that different senescence stimuli generate distinct patterns of DNA methylation changes (Kwiatkowska et al, 2023; Lowe et al, 2016) suggests that methylome changes occur as a result of the senescence process, however, other studies show that manipulation of the DNA methylation machinery causes DNA methylation loss and induces senescence (Chen et al, 2025; Jung et al, 2017; Yang et al, 2017). Thus, the relationship between DNA methylation and senescence is complex, as in some cases methylome repatterning can trigger senescence and in others, oncogenic stimuli cause cancer-promoting changes in the methylome.

The epigenetic regulator ubiquitin, PHD, and ring finger domain containing protein 1 (UHRF1) regulates the maintenance of DNA methylation during DNA replication (Bostick et al, 2007; Qian et al, 2008; Sharif et al, 2007; Yamaguchi et al, 2024), recruits histone methyltransferases that deposit repressive histone modifications (Kong et al, 2019; Mancini et al, 2021), and is required for DNA repair (Mancini et al, 2021; Zhang et al, 2016). Our work shows that overexpression of human UHRF1 in zebrafish hepatocytes *Tg(fabp10a:hsa.UHRF1-EGFP)*^*mss1a*^ (hereafter referred to as hUHRF1) causes liver cancer (Mudbhary et al, 2014). This provided functional evidence that an epigenetic regulator can be an oncogene, which is important as UHRF1 is upregulated in nearly every type of cancer (Kim and Benavente, 2024), including HCC (Liang et al, 2015; Liu et al, 2017; Mudbhary et al, 2014; Wang et al, 2023). We previously showed that within 2 days of hUHRF1 overexpression in zebrafish hepatocytes, classical markers of senescence, including *tp53*, SA-β-gal-positive staining, and cell cycle withdrawal, were present, with liver cancer developing 2 weeks later (Mudbhary et al, 2014). We used this system to investigate the mechanism of UHRF1-induced senescence and delineate the fate of cells that overexpress hUHRF1. We report that this single oncogene can induce pleotropic senotypes that evolve over time. Within hours of hUHRF1 overexpression, hepatocytes withdraw from the cell cycle, induce TE expression, and have DNA double-strand breaks (DSBs), activating Atm and Tp53 to promote some, but not all, features of senescence. As senescence progresses, distinct populations of senescent cells appear, which are distinguished by hUHRF1 levels, Tp53 activation, and mitogen responsiveness. At later stages, cells with reduced hUHRF1 levels can proliferate when Tp53 is removed, whereas cells with the highest hUHRF1 expression cannot. Navitoclax treatment of *hUHRF1* larvae increases cell death, reduces SA-β-gal staining, and reduces heterogeneity in hUHRF1-EGFP expression in preneoplastic livers, sparing cells that have lower levels of hUHRF1. This suggests that in UHRF1-driven cancers, senolytic treatment may inadvertently promote tumor growth and highlight the importance of understanding senescence heterogeneity in cancer progression.

## Results

### UHRF1 overexpression rapidly induces a DDR and SASP

**Figure 1:**
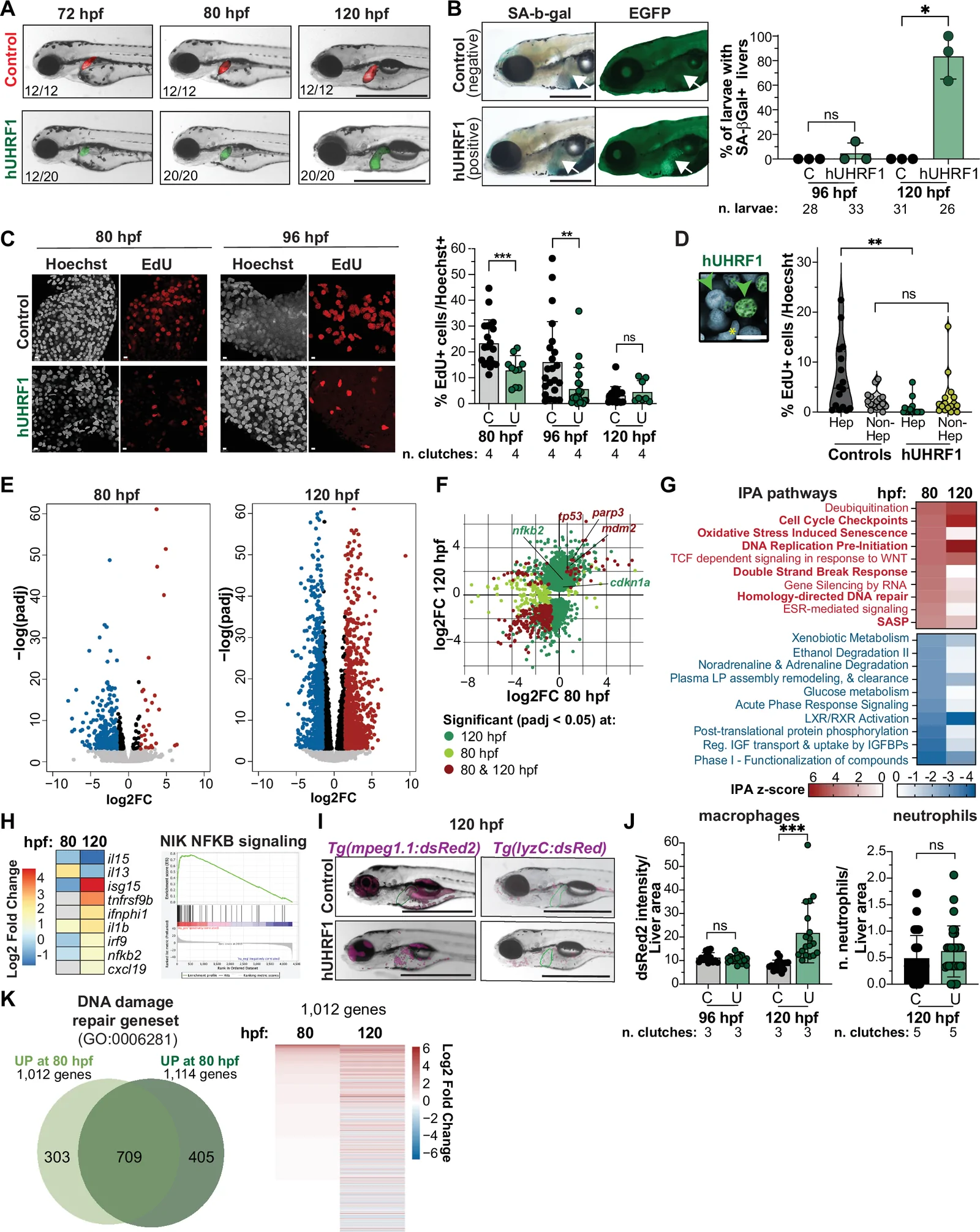
UHRF1 overexpression rapidly induces a DDR and SASP. (**A**) Images of live *hUHRF1* and control *Tg(fabp10a:nls-mCherry)* larvae at 72, 80, and 120 hpf. The number of larvae with fluorescent livers at each time point is indicated over the total number of larvae assessed in 1 clutch. Scale bar: 1 mm. (**B**) Representative images of *hUHRF1* and WT sibling (control) stained with SA-β-galactosidase (SA-βGal) at 120 hpf and the EGFP signal. The arrow points to the liver. The image's background has been modified to uniform white. Scale bar: 0.5 mm. Percent of positive larvae in each biological replicate at 96 and 120 hpf. Dots indicate the number of biological replicates (clutches), and the total number of larvae is indicated. Data are presented as mean ± SD. *P value < 0.05 by unpaired *t* test. Exact *P* value for the annotated comparison is: *hUHRF1* versus controls at 96 hpf, *P* value = 0.4226; *hUHRF1* versus controls at 120 hpf, *P* value = 0.016. (**C**) Representative images of EdU in *hUHRF1* and WT sibling livers (control) at 80 and 96 hpf. Percentage of EdU-positive cells at 80, 96, and 120 hpf normalized to the total number of cells in each sample. Each dot indicates the percent positive EdU cells per liver in four biological replicates (clutches). Data are presented as mean ± SD. ***P value < 0.001, **P value < 0.01 by unpaired *t* test. Exact *P* value for the annotated comparison are: *hUHRF1* versus controls at 80 hpf, *P* value = 0.004479; *hUHRF1* versus controls at 96 hpf, *P* value = 0.007979; *hUHRF1* versus controls at 120 hpf, *P* value = 0.2362. Scale bar: 5 μm. (**D**) Cells in the liver at 96 hpf were scored as hepatocytes based on round nuclear morphology (green arrow in inset) and non-hepatocytes with non-spherical nuclei (* in inset). Each dot indicates the percent positive EdU cells per liver in four biological replicates (clutches). **P value < 0.01 by unpaired *t* test. Exact *P* value for the annotated comparison is: *hUHRF1* versus controls in Hep, *P* value = 0.0006; *hUHRF1* versus controls in non-Hep, *P* value = 0.5313. Scale bar: 10 μm. (**E**) Volcano plot of differentially expressed genes in three biological replicates of *hUHRF1* compared two biological replicates of control livers at 80 and six biological replicates of *hUHRF1* compared to six biological replicates of control livers at 120 hpf. Gray dots indicate genes with *P* value adjusted >0.05, black dots indicate genes with *P* value adjusted <0.05, red dots indicate genes with *P* value adjusted <0.05 and log2 fold change >1.5, and blue dots indicate genes with *P* value adjusted <0.05 and log2 fold change < −1.5. *P* values were calculated using the Wald test implemented in DESeq2 and adjusted for multiple testing using the Benjamini–Hochberg method. Genes with an adjusted *P* < 0.05 were considered significantly differentially expressed. (**F**) Crossplot of the Log2 fold change (Log2FC) of all differentially expressed genes in *hUHRF1* compared to control livers at 80 and 120 hpf. At each time point, *P* values were calculated using the Wald test implemented in DESeq2 and adjusted for multiple testing using the Benjamini–Hochberg method. Genes with an adjusted *P* < 0.05 were considered significantly differentially expressed. (**G**) The top ten Ingenuity Pathway Analysis (IPA) upregulated (red) and downregulated pathways (blue) in *hUHRF1* livers compared to controls at 80 hpf and their relative changes at 120 hpf. Pathways relevant to senescence are in bold. (**H**) Heatmap of log2 fold change of a subset of genes associated with SASP at 80 and 120 hpf, and the Gene Set Enrichment plot of the NFKB pathway identified from RNAseq analysis of *hUHRF1* compared to control livers at 120 hpf. (**I**) Representative images of macrophages (purple) in *Tg(mpeg1.1:dsRed2)* and neutrophils (purple) in *Tg(lyz:dsRed)* in *hUHRF1* and control larvae at 120 hpf. The liver is outlined in green. Scale bar: 1 mm. (**J**) Relative fluorescence intensity of macrophages and number of neutrophils over the left liver lobe at 96 and 120 hpf. Scale bar: 1 mm. Each dot indicates one liver, and the number of biological replicates (clutches) is indicated in the figure. Data are presented as mean ± SD. ***P value < 0.001 by unpaired *t* test. Exact *P* value for the annotated comparison are: macrophages in *hUHRF1* versus controls at 96 hpf, *P* value = 0.228982; macrophages in *hUHRF1* versus controls at 120 hpf, *P* value = 0.000282; neutrophils in *hUHRF1* versus controls at 120 hpf, *P* value = 0.2752. (**K**) Venn diagram of DNA damage repair genes (GO: 0006281) upregulated at 80 and 120 hpf (log2 fold change >0). Heatmap of upregulated DNA damage repair genes upregulated at 80 hpf compared to their expression (log2 fold change) at 120 hpf. Source data are available online for this figure.

The *fabp10a* promoter drives transgene expression in hepatocytes as soon as they differentiate (Her et al, 2003). A control transgene under this promoter (*Tg*(*fabp10a:nls-mCherry*)) can be detected in the liver of all larvae starting at 72 h post-fertilization (hpf), whereas EGFP can be detected in 100% of *hUHRF1* larvae only at 80 hpf and later (Fig. 1A). A SA-β-gal staining protocol optimized for whole mount zebrafish larvae (Magnani et al, 2024; Mudbhary et al, 2014) was negative in nearly all larvae at 96 hpf, but by 120 hpf, over 80% of *hUHRF1* livers had positive staining (Fig. 1B) as previously reported (Mudbhary et al, 2014). Cell cycle withdrawal, as a defining feature of senescence, was investigated using EdU incorporation at 80, 96, and 120 hpf, showing 25%, 15%, and 2% EdU-positive cells in controls, respectively, and a significant reduction to 15% and 5% in *hUHRF1* larvae at 80 and 96 hpf. There was no difference in EdU incorporation at 120 hpf, since nearly all cells in control livers had completed S-phase at this stage (Fig. 1C). Using nuclear morphology to identify hepatocytes, we found that both hepatocytes and non-hepatocytes incorporate EdU in control livers at 96 hpf, but virtually no hepatocytes incorporated EdU in *hUHRF1* livers (Fig. 1D). This indicates that within less than 24 h of hUHRF1 overexpression, cells withdraw from the cell cycle.

We eliminated the possibility that the transgene insertion caused senescence due to a bystander effect by showing that CRISPR-Cas9 mutagenesis of the transgene (Appendix Fig. S1A,B) rescued SA-β-gal staining (Appendix Fig. S1C). We mapped the insertion of the transgene to Chromosome 14 and analyzed bulk RNAseq data from livers from *hUHRF1* and control larvae at 80 hpf and 120 hpf (Dataset EV1) to examine the expression of genes at this locus (Appendix Fig. S1D). None were altered in *hUHRF1* livers (Appendix Fig. S1E), eliminating the likelihood of a positional effect of the transgene.

To determine the immediate and sustained transcriptional response to hUHRF1 overexpression, we assessed transcriptomic changes at 80 hpf, the earliest time point when hUHRF1-EGFP is detected in 100% of larvae (Fig. 1A) compared to the time when the senescence phenotype is fully penetrant at 120 hpf characterized by robust SA-β-gal staining (Fig. 1B) and activation of *tp53* and its downstream targets (Ajouaou et al, 2023; Mudbhary et al, 2014). At 80 hpf, there were 445 significantly differentially expressed genes (DEGs; *P* value adjusted <0.05), with 51 upregulated and 394 downregulated, compared to 5353 DEGs at 120 hpf (*P* value adjusted <0.05), with 2640 upregulated and 2713 downregulated (Fig. 1E; Dataset EV1). Importantly, most of the DEGs identified at 80 hpf were similarly expressed at 120 hpf, including *tp53* and its key targets (Fig. 1F).

Ingenuity Pathway Analysis (IPA) of DEGs at 80 hpf showed that SASP, cell cycle checkpoint, and the response to double-strand breaks (DSB) were induced at 80 hpf and were sustained through 120 hpf (Fig. 1G). Downregulated pathways at both time points reflected a range of hepatocyte functions (Fig. 1G). Interestingly, some pathways were enriched at only 1 time point, such as many SASP genes and NF-kB, a key upstream immune regulator, which were induced only modestly or not at all at 80 hpf but were highly induced at 120 hpf (Fig. 1H). Similarly, transgenic reporter lines for macrophages, and neutrophils ((*Tg(mpeg1.1:dsRed)* and *Tg(lyzC:dsRed)*, respectively), showed an increased number of macrophages the liver starting at 120 hpf while neutrophils were not increased either timepoint (Fig. 1I,J).

Given the importance of the DDR in senescence, we examined expression of the DDR gene set (GO:0006281) at both time points. This showed that 1012 genes were induced at 80 hpf, 70% of which were also induced at 120 hpf (Fig. 1K), suggesting a persistent DDR. Together, these data show that markers of DDR and cell cycle withdrawal are an early response to hUHRF1 overexpression, and that these responses evolve over time, with SASP, macrophage recruitment, and SA-β-gal staining developing later. This suggests an evolution of the senescence phenotype over the course of 2 days following hUHRF1 overexpression.

### DNA damage and TE activation associated with DNA methylation loss are early responses to hUHRF1 overexpression

**Figure 2:**
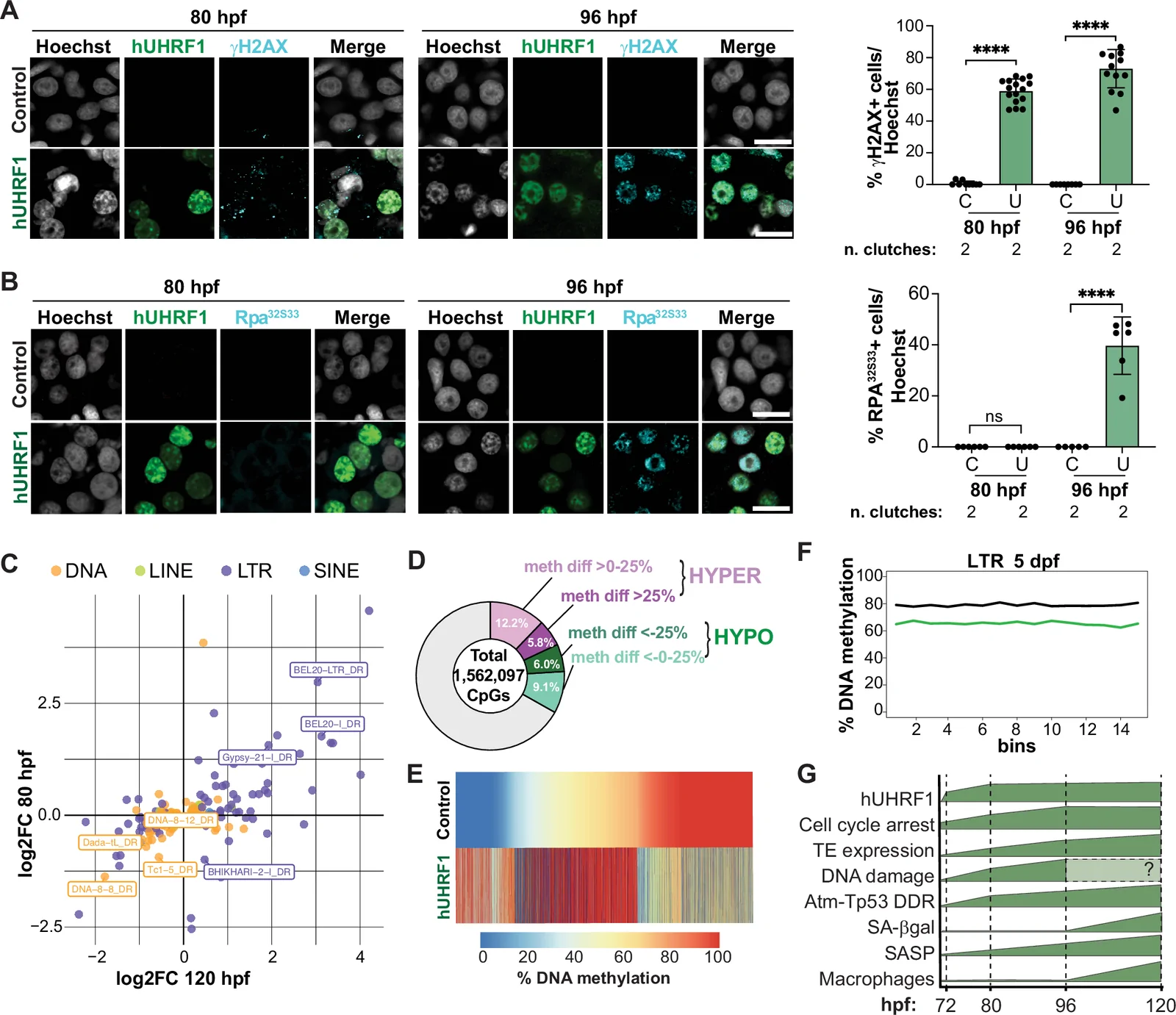
DNA damage and TE activation associated with DNA methylation loss are early responses to hUHRF1 overexpression. (**A**) Immunofluorescence of γH2AX in *hUHRF1* (U) and wild-type sibling (control; C) at 80 and 96 hpf. Percentage of γH2AX-positive cells at 80 and 96 hpf normalized to the total number of cells in each sample. Each dot represents one liver in two biological replicates (clutches). Data are presented as mean ± SD. ****P value < 0.0001 by unpaired *t* test. Exact *P* value for the annotated comparison are: *hUHRF1* versus controls at 80 hpf, *P* value = 2.67098 × 10^−16^; *hUHRF1* versus controls at 96 hpf, *P* value = 2.70654×10 − ^13^. Scale bar: 20 μm. (**B**) Immunofluorescence of phosphorylated Rpa (Rpa32S33) in *hUHRF1* (U) and wild-type sibling (control; C) at 80 and 96 hpf. Percentage of Rpa32S33 positive cells at 80 and 96 hpf normalized to the total number of cells in each sample. Each dot represents one liver in two biological replicates (clutches). Data are presented as mean ± SD. ****P value < 0.0001 by unpaired *t* test. Exact *P* value for the annotated comparison are: *hUHRF1* versus controls at 96 hpf, *P* value = 0.0000264. Scale bar: 20 μm. (**C**) Crossplot of log2 fold change of differentially expressed TEs in *hUHRF1* compared to control livers at 80 and 120 hpf. (**D**) Pie chart classification of RRBS analysis of livers from 120 hpf control and *hUHRF1* larvae. All CpGs with 10X coverage in all samples were categorized as hypermethylated or hypomethylated by an FDR < 0.01 and then classified as 0–25% change or >25% change in *hUHRF1* livers compared to controls. (**E**) Heatmap of CpGs that were significantly differentially methylated (FDR < 0.01) and had an effect size of >25% change, rank-ordered by level of methylation in controls. (**F**) Metaplot of CpG methylation levels on all LTR transposons in *hUHRF1* (green) and control (black) livers at 120 hpf. (**G**) Time course of senescence-associated phenotypes caused by hUHRF1 overexpression in hepatocytes at 80, 96, and 120 hpf. Source data are available online for this figure.

The enrichment of genes involved in the DSB response at 80 and 120 hpf suggested that hUHRF1 overexpression induced DNA damage. To assess this, we stained for γH2AX foci, which is commonly used as a marker of double-strand breaks (DSBs), and phosphorylated replication protein A (p-Rpa), as it accumulates on single-strand DNA during replication stress or DSBs repair. γH2AX foci were absent from control nuclei at all stages, but were detected in over half the liver cells in *hUHRF1* larvae as early as 80 hpf, and this persisted to 96 hpf (Fig. 2A). p-Rpa was not detected in hepatic cells of *hUHRF1* larvae at 80 hpf, but was found in less than half of the cells at 96 hpf (Fig. 2B). These data, together with the strong decrease of EdU in hepatocytes as early as 80 hpf (Fig. 1C,D), suggest that hUHRF1 overexpression causes DSBs followed by the formation of single-stranded DNA. It is also possible that p-Rpa staining could be attributed to ongoing DNA repair, or multiple mechanisms of DNA damage in this model, potentially attributed to hUHRF1 levels or the cell cycle stage in which the damage occurs.

UHRF1 is required for DNA methylation (Bostick et al, 2007; Sharif et al, 2007), a primary mechanism of TE suppression. Since active TEs can cause DSBs (Gasior et al, 2006), we investigated TE expression in bulk RNAseq of *hUHRF1* livers. LTR retrotransposons were induced at both 80 hpf and 120 hpf (Fig. 2C), indicating that the epigenetic mechanisms suppressing these TEs are deregulated as early as 80 hpf. We therefore profiled DNA methylation at 120 hpf, which was the earliest time point when we could obtain sufficient genomic DNA from pools of dissected livers to carry out low-input reduced representation bisulfite sequencing (RRBS). We obtained 10X coverage of over 1.5 million CpGs in all samples analyzed. hUHRF1 overexpression caused a change in methylation levels in nearly 1/3 of all CpGs analyzed, with 15% of all CpGs having lost methylation (Fig. 2D; Table EV1). Loss of methylation was most pronounced on the CpGs that were fully methylated in controls, while methylation gain was detected in regions that were partially methylated in controls (Fig. 2E). Importantly, LTR retrotransposons were hypomethylated in *hUHRF1* livers (Fig. 2F), reminiscent of the profile in zebrafish mutants where DNA methylation is depleted (Chernyavskaya et al, 2017; Magnani et al, 2021). This shows that hUHRF1 overexpression causes DNA hypomethylation and derepression of TEs, providing a potential mechanism of DNA damage in this model. Together, these data define the temporal sequence of cellular and molecular responses to hUHRF1 overexpression, with TE activation, DNA damage, DDR activation, and cell-cycle arrest occurring within hours, followed by SASP, macrophage recruitment, and SA-β-gal-positive staining within 2 days (Fig. 2G). We hypothesize that these events are a downstream effect of DNA methylation changes caused by UHRF1 expression.

### The *atm*-*tp53* pathway is required for distinct UHRF1-induced senescence features

**Figure 3:**
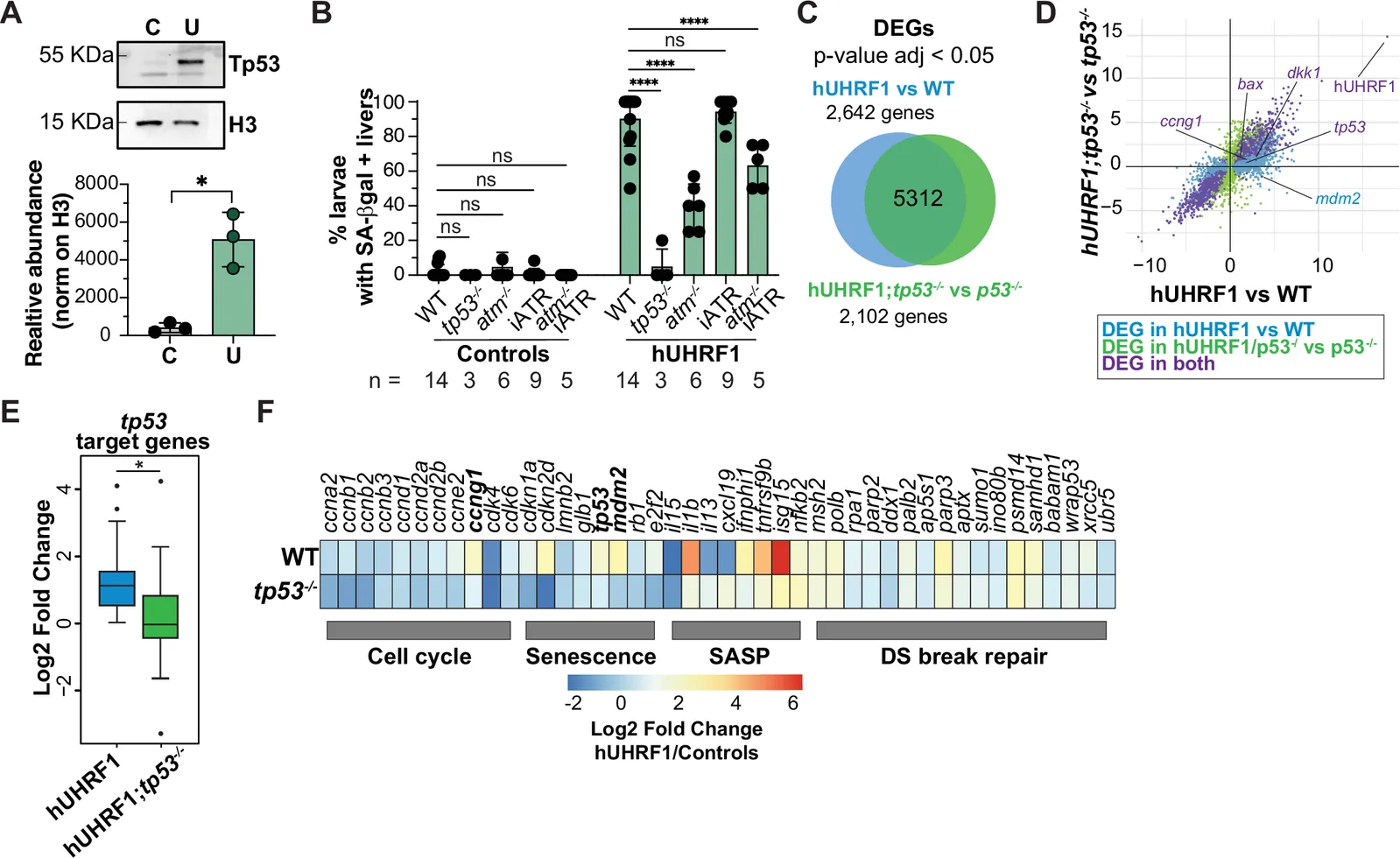
The *atm*-*tp53* pathway is required for distinct UHRF1-induced senescence features. (**A**) Western blot of Tp53 in *hUHRF1* (U) and controls (C) livers at 5 dpf. Quantification of Tp53 is normalized to Histone 3 (H3). Data are presented as mean ± SD of three biological replicates (clutches). *P value < 0.05 by unpaired *t* test. Exact *P* value for the annotated comparison are: *hUHRF1* versus controls, *P* value = 0.0275. (**B**) Percentage of SA-β-gal-positive livers at 5 dpf in *hUHRF1* and control livers in WT background, with mutation of *tp53, mutation of atm*, chemical inhibition of Atr (iATR), or combination of *atm* mutation and iATR. ****P value < 0.0001 by unpaired *t* test. Dots represent biological replicates (clutches), and the exact number of biological replicates (*n*) is indicated in the figure. Data are presented as mean ± SD. Exact *P* value for the annotated comparison are: *tp53*^*−/−*^ versus WT for controls, *P* value = 0.999999; *atm*^*−/−*^ versus WT for controls, *P* value = 999829; iATR versus WT for controls, *P* value = 1; *atm*^*−/*^*;iATR* versus WT for controls, *P* value = 999995; *hUHRF1* versus *hUHRF1; tp53*^*−/−*^, *P* value = 1.4 × 10^−14^; *hUHRF1* versus *hUHRF1; atm*^*−/−*^, *P* value = 3.09 × 10^−14^; *hUHRF1* versus *hUHRF1; iATR*, *P* value = 0.991884; *hUHRF1* versus *hUHRF1; atm*^*−/*^*; iATR*^*−*^, *P* value = 4.69 × 10^−5^. (**C**) Venn diagram of differentially expressed genes (DEGs) in the liver of *hUHRF1* compared WT or in *hUHRF1;tp53*^*−/−*^ compared *tp53*^*−/−*^ of five biological replicates (clutches) for each condition. *P* values were calculated using the Wald test implemented in DESeq2 and adjusted for multiple testing using the Benjamini–Hochberg method. Genes with an adjusted *P* < 0.05 were considered significantly differentially expressed. (**D**) Crossplot of log2 fold change of differentially expressed genes (DEGs) in *hUHRF1* compared to WT controls or *hUHRF1*;*tp53*^*−/−*^ to *tp53*^*−/−*^ at 120 hpf. For each comparison, *P* values were calculated using the Wald test implemented in DESeq2 and adjusted for multiple testing using the Benjamini–Hochberg method. Genes with an adjusted *p* < 0.05 were considered significantly differentially expressed. (**E**) Box plot of the log2 fold change of *tp53* target genes (Fischer, 2017) upregulated in *hUHRF1* on a WT background (WT) compared to the expression of the same genes in the liver of *hUHRF1*;*tp53*^*−/−*^ compared to *tp53*^*−/*^ of five biological replicates (clutches) for each condition. The median of the distribution is represented by the horizontal line, and the boundaries of the boxes represent quartiles, while the whiskers mark the 10th and 90th percentiles. *P value < 0.05 by Wilcoxon paired test. Exact *P* value for the annotated comparison are: *hUHRF1* versus *hUHRF1; tp53*^*−/−*^, *P* value = 0.0333. (**F**) Heatmap of a subset of Tp53 target genes relevant to senescence in *hUHRF1* on a WT background (WT) compared to the expression of the same genes in *hUHRF1*;*tp53*^*−/−*^ compared to *tp53*^*−/−*^. Source data are available online for this figure.

*tp53*, *atm* and Tp53 downstream targets are induced at 120 hpf in *hUHRF1* livers (Ajouaou et al, 2023; Mudbhary et al, 2014). Tp53 activation was confirmed by Western Blotting of dissected livers, showing a significant induction of Tp53 protein in *hUHRF1* livers at 5 dpf (Fig. 3A). We investigated the requirement for Tp53 and its upstream activators, Atm and Atr, in hUHRF1-mediated senescence. Homozygous loss-of-function mutation in *tp53* (Berghmans et al, 2005) or *atm* (Ajouaou et al, 2023) abrogated SA-β-gal staining in *hUHRF1* larvae (Fig. 3B). In contrast, the Atr inhibitor VE-821 used at a concentration that eliminated phosphorylated Chk1 in response to peroxide-induced damage (Appendix Fig. S2) and was effective in mitigating replication stress in another zebrafish model (Madakashira et al, 2024), did not reduce SA-β-gal staining alone or in combination with *atm* mutation (Fig. 3B). This suggests that both Atm and Tp53, but not Atr, are required for hUHRF1-induced SA-β-gal. While these data suggest Atr does not compensate for *atm* loss in this early stage of UHRF1-induced changes, it does not rule out the possibility that Atr plays a role in a subset of senescent cells, which could not be detected through this assay, or that Atr may have other roles in response to hUHRF1 deregulation.

**Figure EV1:**
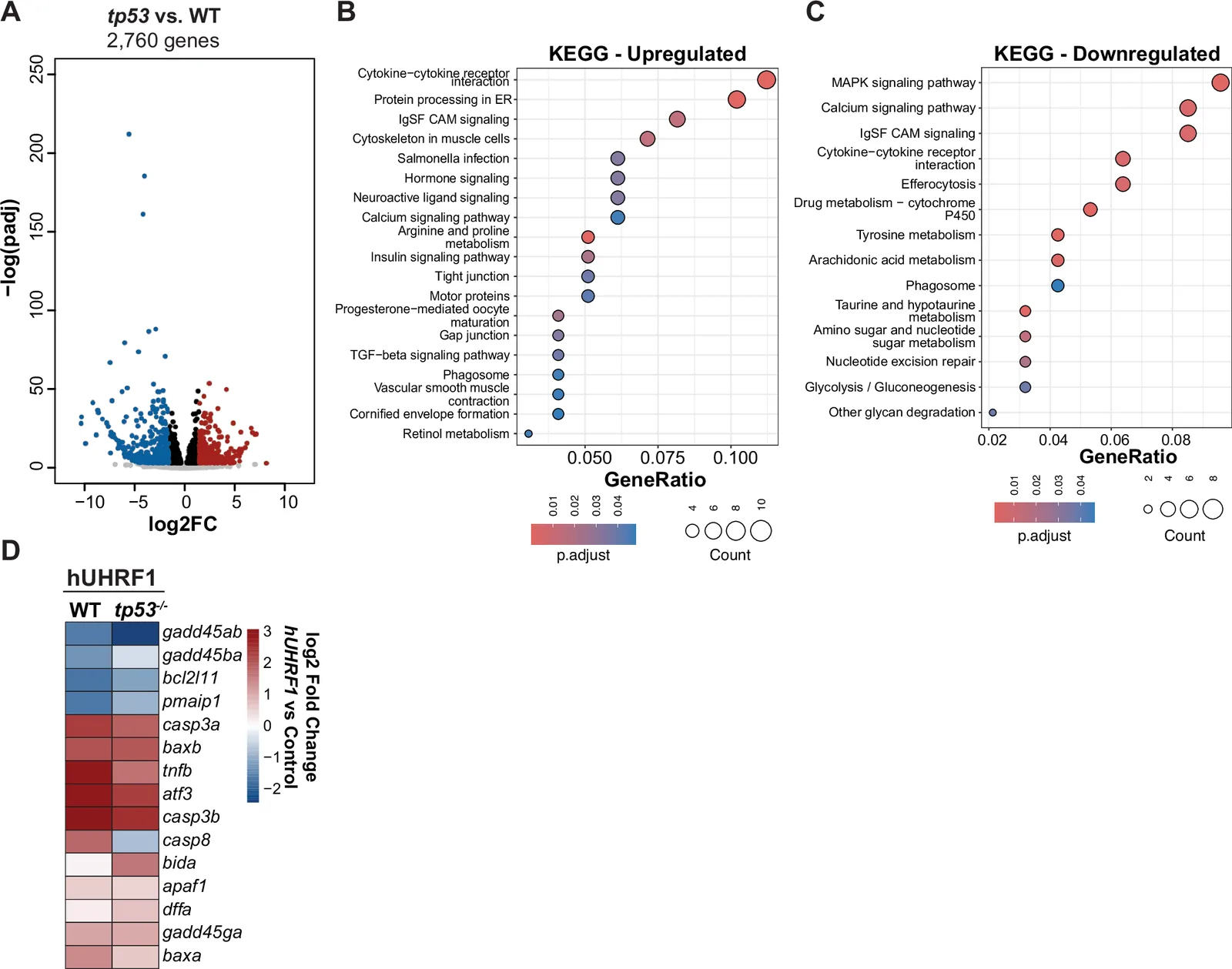
Bulk RNAseq shows many differentially expressed genes in the liver of *tp53*^*−/−*^mutants. (**A**) Volcano plot of differentially expressed genes in the liver of *tp53*^*−/−*^ larvae compared to wild-type at 120 hpf in five biological replicates. Gray dots indicate genes with *P* value adjusted >0.05, black dots indicate genes with *P* value adjusted <0.05, red dots indicate genes with *P* value adjusted <0.05 and log2 fold change >1.5, and blue dots indicate genes with *P* value adjusted <0.05 and log2 fold change < −1.5. *P* values were calculated using the Wald test implemented in DESeq2 and adjusted for multiple testing using the Benjamini–Hochberg method. Genes with an adjusted *P* < 0.05 were considered significantly differentially expressed. (**B**) KEEG pathway analysis of upregulated genes (*P* value adjusted <0.05 and log2 fold change >0). KEGG pathways enrichment was assessed using a one-sided hypergeometric test with Benjamini–Hochberg correction for multiple testing. Dot size represents the gene ratio, and color represents −log10 of the adjusted *P* value. (**C**) KEEG pathway analysis of downregulated genes (*P* value adjusted <0.05 and log2 fold change <0). KEGG pathways enrichment was assessed using a one-sided hypergeometric test with Benjamini–Hochberg correction for multiple testing. Dot size represents the gene ratio, and color represents −log10 of the adjusted *P* value. (**D**) Heatmap of log2 fold change of genes involved in apoptosis in samples overexpressing hUHRF1, comparing *hUHRF1* to WT (WT) and *hUHRF1;tp53*^*−/−*^ to *tp53*^*−/−*^(*tp53*^*−/−*^).

To investigate the *tp53*-dependent senescence phenotypes induced by hUHRF1 overexpression, we carried out bulk RNAseq on control and *hUHRF1* livers with wild-type (WT) and *tp53*^*−/−*^ background (Fig. 3C; Dataset EV2). *tp53*^*−/−*^ loss had a profound effect on gene expression in the liver even without hUHRF1 overexpression. There were 2,760 DEGs (*P* value adjusted <0.05; Fig. EV1A, Dataset EV2), with deregulated pathways representing a range of cellular functions, including upregulation of signaling pathways and downregulation of metabolic processes (Fig. EV1B). Interestingly, most DEGs caused by *hUHRF1* overexpression did not change when *tp53* was removed (Fig. 3C,D), with the exception of canonical *tp53* target genes (Fischer, 2017) such as *mdmd2*, *bax*, *dkk1*, *ccng1*, and *gadd45ab* (Figs. 3D,E and EV1C). Importantly, genes involved in senescence and SASP were reduced in *hUHRF1*;*tp53*^*−/−*^ livers, while genes involved in apoptosis (Fig. EV1C) and many genes in the DDR pathway were largely unchanged (Fig. 3F). Notably, all the genes that drive cell division were suppressed in *hUHRF1* livers, even when *tp53* was removed (Fig. 3F; Dataset EV2). Together, these data show that hUHRF1 overexpression induces DNA damage and activates the Atm-Tp53 pathway, which in turn induces senescence features, including SA-β-gal activity and the SASP. However, at this early stage of senescence, *tp53* loss is insufficient to allow UHRF1-overexpressing cells to re-enter the cell cycle, as seen in other models (Reyes et al, 2018).

### UHRF1 overexpression in hepatocytes changes liver composition and hepatocyte function

**Figure 4:**
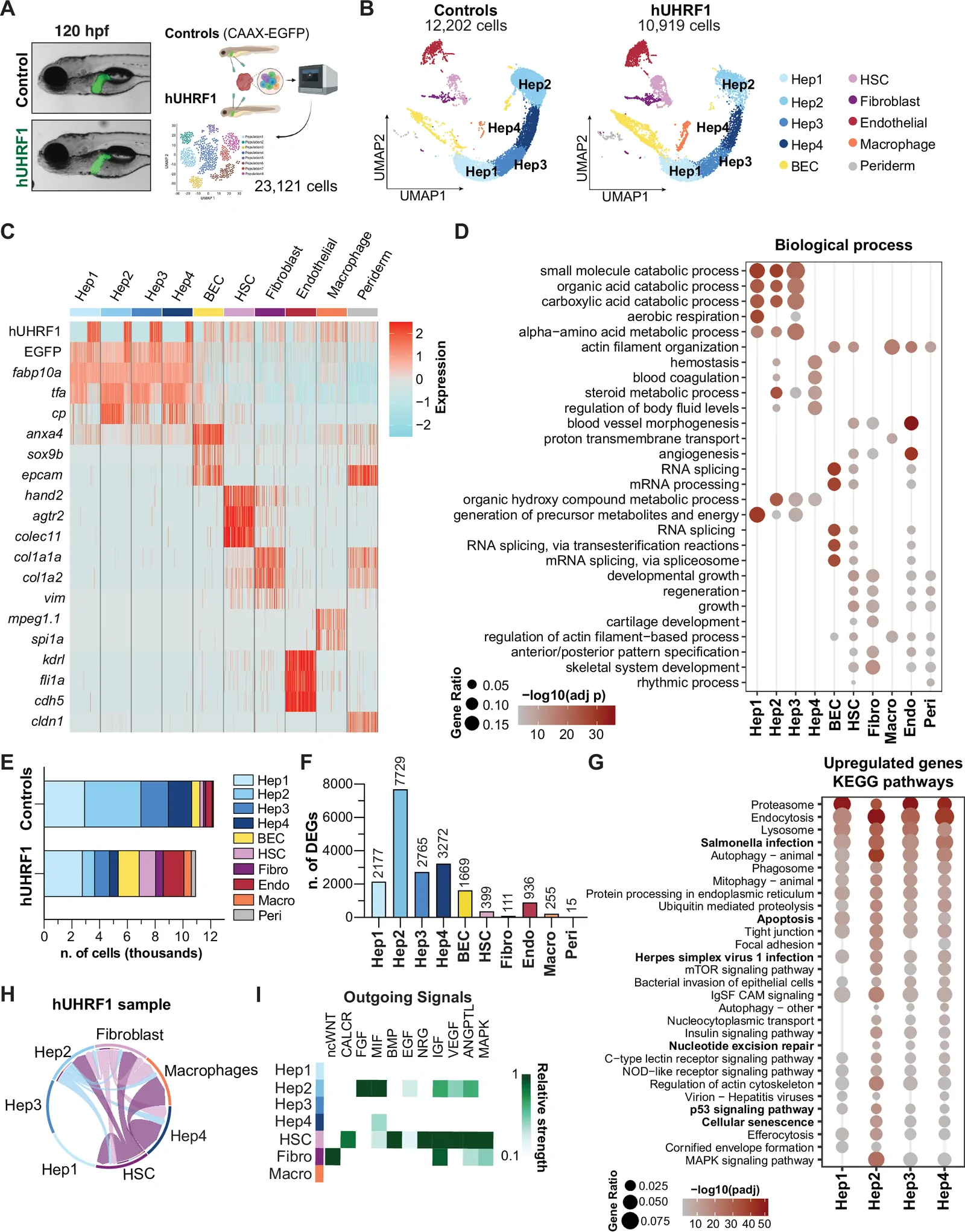
UHRF1 overexpression in hepatocytes changes liver composition and hepatocyte function. (**A**) Scheme of the approach and representative images at 120 hpf of samples used for scRNAseq analysis. (**B**) UMAP plot of cells analyzed by scRNAseq of *hUHRF1* and control livers. (**C**) Heatmap of identity markers in hepatic cell populations pooled for controls and *hUHRF1* samples. (**D**) Gene Ontology of enriched genes (*P* value adjusted <0.05 & Log2FC > 0.5) in each cell population from the combined dataset. GO Biological Process enrichment was assessed using a one-sided hypergeometric test with Benjamini–Hochberg correction for multiple testing. Dot size represents the gene ratio, and color represents −log10 of the adjusted *P* value. (**E**) Number of cells in each population in *hUHRF1* and control livers. (**F**) Number of differentially expressed genes (DEGs; *P* value adjusted <0.05) in *hUHRF1* compared to controls in each population. (**G**) KEGG pathways analysis of differentially expressed genes (*P* value adjusted <0.05 & Log2FC > 1.5) in *hUHRF1* compared to controls for each population of hepatocytes. KEGG pathways enrichment was assessed using a one-sided hypergeometric test with Benjamini–Hochberg correction for multiple testing. Dot size represents the gene ratio, and color represents −log10 of the adjusted *P* value. (**H**) Chord diagram showing cell–cell communication in *hUHRF1* sample, with the width of each chord representing the proportion of cells with that signal and the arrows representing the direction of the signal. (**I**) The top outgoing signaling molecules in each cell population in the *hUHRF1* sample are plotted as the relative strength of the signal.

To identify cell-type-specific gene expression changes caused by hUHRF1 overexpression, we performed single-cell RNAseq (scRNAseq) on pools of manually dissected livers from 120 hpf *hUHRF1* and control larvae expressing membrane-targeted EGFP under the same hepatocyte promoter (*Tg(fabp10a:CAAX-EGFP)*; hereafter referred to as controls; Fig. 4A). This time point was chosen because it was the stage at which all senescence markers were present (Fig. 2G), and a sufficient number of liver cells could be isolated for sequencing. After quality control, the combined dataset contained 23,121 cells segregated into 10 clusters, each assigned to a different hepatic cell type according to expression of established cell identity markers (Morrison et al, 2022; Oderberg and Goessling, 2023) (Fig. 4B,C; Dataset EV3 and Appendix Fig. S3A). There were four populations of hepatocytes and, as expected, together these constituted the majority of all cells (15,986 cells; 69.1% of total). Biliary epithelial cells (BEC), characterized by the expression of *alcama*, *sox9b*, *epcam* and keratin genes, represented the next largest population of cells in the combined dataset (2216 cells; 9.2% of total), followed by endothelial and mesenchymal cells, which were divided into two populations, hepatic stellate cells (HSC) and fibroblasts based on canonical markers (Oderberg and Goessling, 2023), which showed distinct and overlapping gene sets reflecting their function (Fig. 4B–D). It is not known whether the increase in representation of mesenchymal cells represents collection bias or expansion of these populations in *hUHRF1* samples. The expansion of macrophages in the *hUHRF1* dataset (Fig. 4B,E) supports imaging data showing macrophage infiltration in the liver (Fig. 1I). The hUHRF1 and EGFP sequences were added to the danRer10 genome to allow transgene mapping. In both samples, the transgene was expressed primarily in hepatocyte populations (Appendix Fig. S3B,C).

**Figure EV2:**
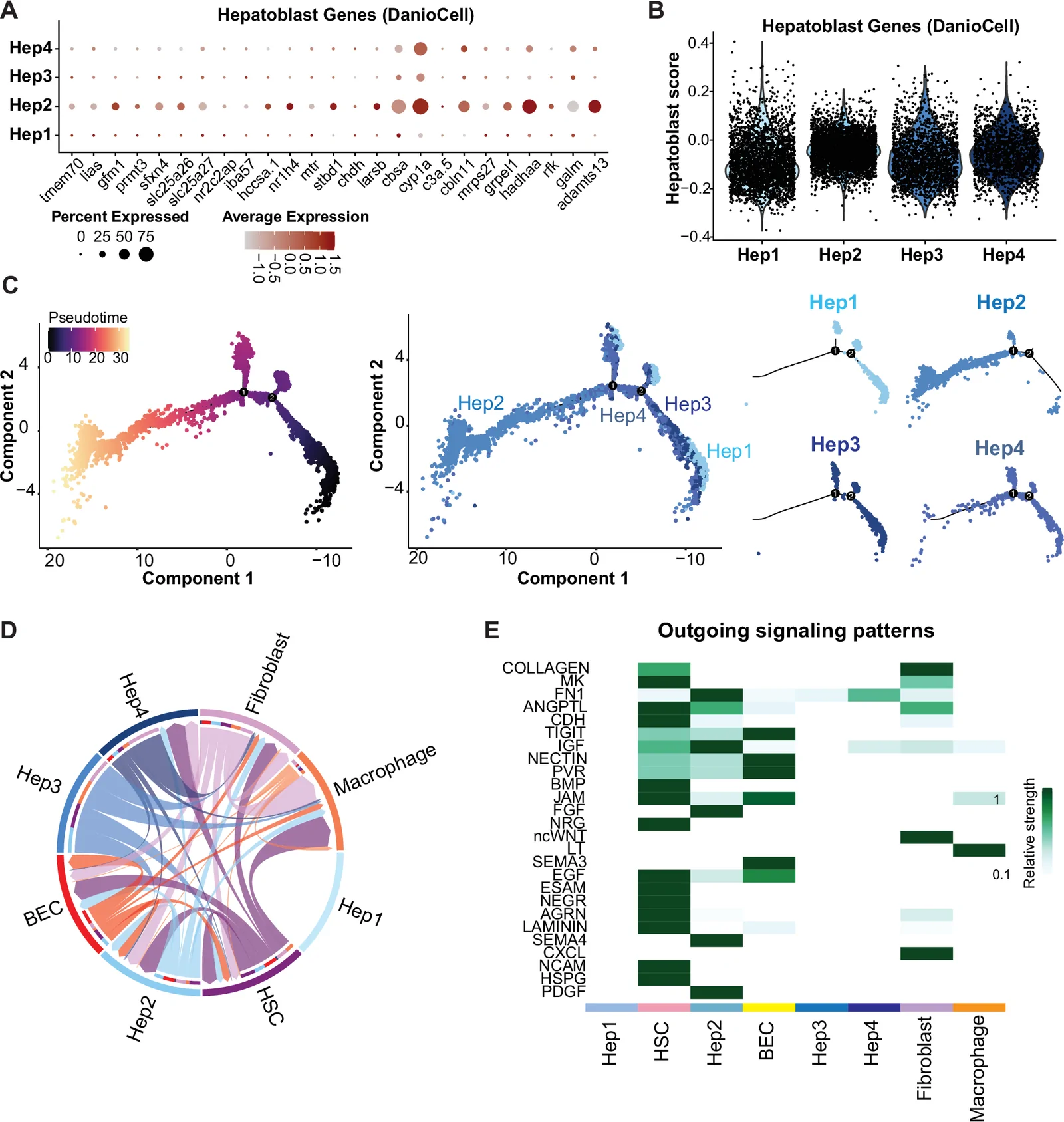
Hepatocytes in control larvae cluster based on expression profile reflecting maturation. (**A**) Expression of hepatoblast genes obtained from DanioCell in hepatocytes from control larvae at 120 hpf. (**B**) Hepatoblast Score of genes present in A in Hep1-4 in controls graphed in individual cells identified from scRNAseq. (**C**) Pseudotime projection of hepatocytes in controls. (**D**) Chord diagram showing cell–cell communication in control, with the width of each chord representing the proportion of cells with that signal and the arrows representing the direction of the signal. (**E**) The top outgoing signaling molecules in control are inferred based on the relative strength of the signaling.

Gene Ontology analysis of significantly enriched genes in each population (*P* value adjusted <0.05 & Log2 Fold Change >0.5) identified hepatocyte populations with different functions, with Hep4 distinguished from others by pathways associated with hemostasis and blood coagulation while Hep1-3 were enriched in metabolic processes (Fig. 4D). This indicates that, as in mammals (Aizarani et al, 2019) different populations of hepatocytes in zebrafish assume distinct functions. We further investigated what features differentiated the hepatocyte clusters in controls and found that a subset of genes specifically expressed in hepatoblasts as indicated by DanioCell (Sur et al, 2023) were enriched in Hep2 (Fig. EV2A,B). Pseudotime analysis confirmed that Hep2 were the least mature (Fig. EV2C), indicating that different clusters are led by maturation status and metabolic abilities. Interestingly, cell–cell communication analysis using CellChat showed that the HSC sent the most outgoing signals, and reciprocal signaling between Hep2, Hep4, and parenchymal cells was also enriched, while Hep1 had the least interaction (Fig. EV2D). Interestingly, PDGF and FGF, both of which are required for liver development in zebrafish (Kan et al, 2009; Shin et al, 2007; Zhao et al, 2026), were identified in control samples as strong outgoing signals from Hep2, the most immature hepatocyte population (Fig. EV2D).

hUHRF1 overexpression generated a striking population shift (Fig. 4E), with depletion of hepatocyte populations and relative expansion of non-parenchymal cells (Fig. 4B,E). Hep2 stood out as it was the most depleted population in *hUHRF1* samples and had the greatest number of DEGs (Fig. 4E,F). DEGs identified in each hepatocyte population compared to the same population in controls (*P* value adjusted <0.05 & Log2FC > 1.5) were assessed for KEGG pathway enrichment. All hepatocyte populations in *hUHRF1* samples were enriched for Tp53 signaling, apoptosis, and pathways related to infection (Fig. 4G). Hep2, Hep3, and Hep4 populations were enriched for pathways associated with senescence, pro-apoptotic, and proliferative components of the MAPK signaling pathway (Fig. 4G,H). This is consistent with the finding of a modest but significant increase in TUNEL-positive cells (Appendix Fig. S4A) and induction of some apoptosis regulators (*baxa, baxb*, *bbc3* (*puma*), *casp3a*, and *casp3b)* in a subset of cells in both control and *hUHRF1* samples (Appendix Fig. S4B).

CellChat analysis of *hUHRF1* samples showed that the most reciprocal signaling occurred between Hep2 and mesenchymal cells, similar to controls, while Hep4 and macrophages were only involved in receiving signals (Fig. 4H). We investigated the specific signaling pathways involved in each cell type and found that Hep2 cells were enriched for *Angptl* and *Mif*, two important signaling pathways in SASP, and for VEGF, which is important for endothelial outgrowth and the tumor microenvironment. Interestingly, the mitogenic signals IGF, FGF, and EGF were all enriched in Hep2 (Fig. 4I). This demonstrates that major changes in hepatic cell composition and identity occur within 2 days of hUHRF1 overexpression.

### UHRF1 overexpression causes pleiotropic senescence phenotypes

**Figure 5:**
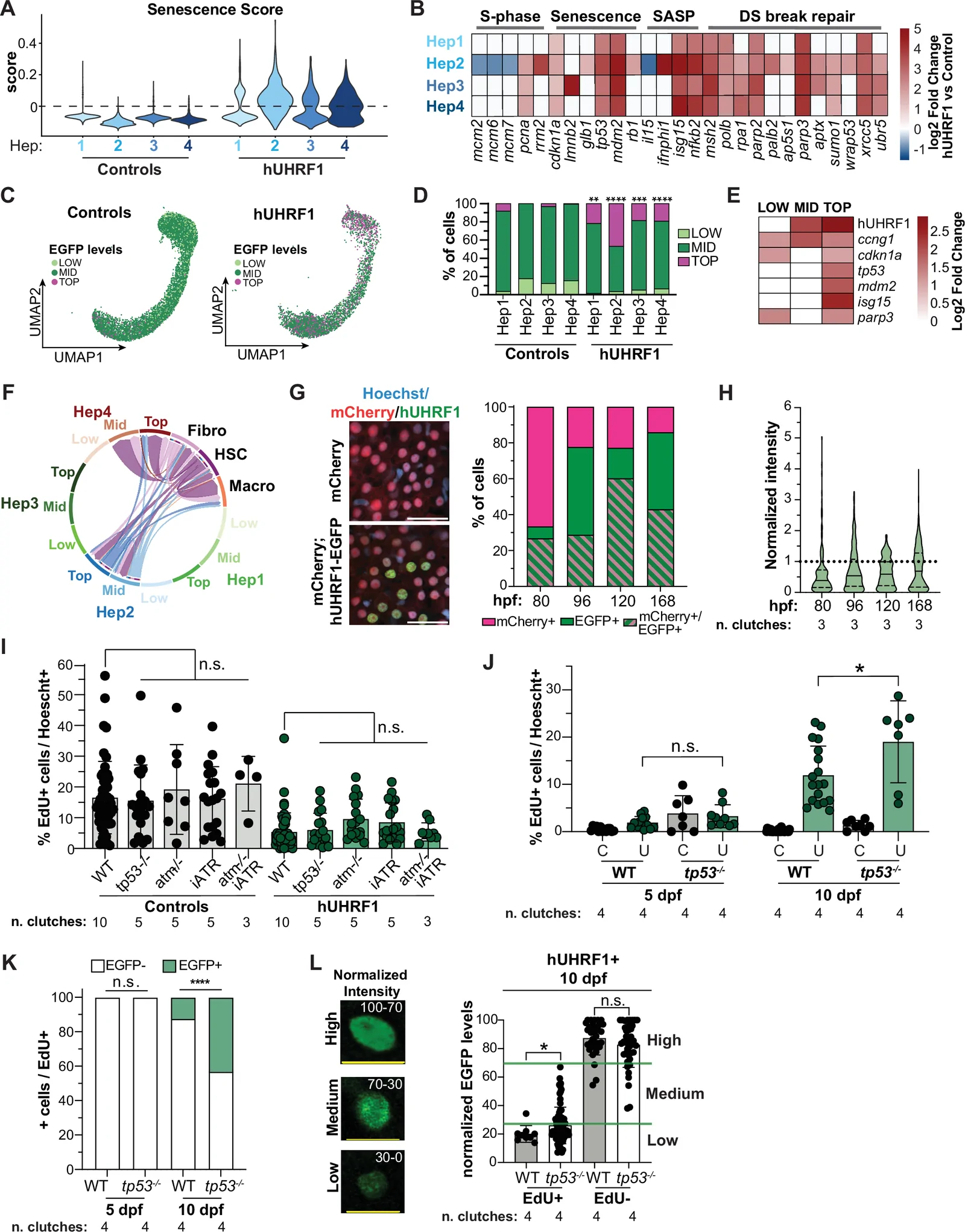
UHRF1 overexpression causes pleiotropic senescence phenotypes. (**A**) Violin plot of Senescence score calculated on all genes in the KEGG Senescence pathway (hsa04218) across all hepatocyte populations identified in scRNAseq. (**B**) Heatmap of key genes involved in senescence and DNA replication. Log2 fold change was calculated from *hUHRF1* samples compared to controls for each population. (**C**) UMAP of hepatocytes divided into high (top 10% EGFP expressing cells), mid, and low (bottom 10% EGFP expressing cells) based on EGFP expression in *hUHRF1* and control livers. (**D**) Percent of cells in each population that have high, mid, or low expression of EGFP. **P value < 0.01, ***P value < 0.001, ****P value < 0.0001 by Chi-square test on percentage of high cells in *hUHRF1* compared controls for each population. Exact *P* value for the annotated comparison are: *hUHRF1* versus controls in Hep1, *P* value = 0.0092; *hUHRF1* versus controls in Hep2, *P* value = 4.569 × 10^−15^; *hUHRF1* versus controls in Hep3, *P* value = 0.0008; *hUHRF1* versus controls in Hep4, *P* value = 4.606 × 10^−6^. (**E**) Heatmap of a panel of genes involved in Senescence. Log2 fold change is calculated in each category of *hUHRF1* levels compared to controls for the same EGFP category. (**F**) Chord diagram of cell–cell communication of Hep populations in *hUHRF1* divided in top, mid and low EGFP expression and other cell types. The width of each chord represents the proportion of cells with that signal, and the arrows represent the direction of the signal. (**G**) Representative images of hUHRF1-EGFP and nls-mCherry in controls and *hUHRF1* livers at 120 hpf. Scale bar: 25 μm. Quantification of cells positive for both EGFP and mCherry, EGFP only, and mCherry only in *Tg(fabp10a:hUHRF1;nls-mCherry)* and controls (mCherry) livers. (**H**) Quantification of the ratio between the fluorescent intensity of EGFP normalized on maximum intensity, and mCherry normalized on maximum intensity for each liver at each time point. The solid line represents the median, and the dashed lines indicate the quartiles. (**I**) EdU incorporation at 96 hpf in *hUHRF1* and control livers in WT, *tp53*^−/−^, *atm*^−/−^, chemical inhibition of Atr (iATR) alone or in combination with *atm*^−/−^. Dots represent individual livers, and the number of biological replicates (clutches) is indicated. n.s. *P* value > 0.05 with unpaired *t* test. Data are presented as mean ± SD. (**J**) EdU incorporation in *hUHRF1*, WT control, *tp53*^*−/−*^*;hUHRF1* and *tp53*^*−/−*^ livers at 5 and 10 dpf. Dots represent individual livers, and the number of biological replicates (clutches) is indicated. Data are presented as mean ± SD. *P value < 0.05 by unpaired *t* test. Exact *P* value for the annotated comparison are: *hUHRF1* versus *hUHRF1; tp53*^*−/−*^, *P* value = 0.03092. (**K**). Percent of EdU-positive cells that are either EGFP-positive or negative. ****P value < 0.0001 by Chi-square test. Exact *P* value for the annotated comparison are: *hUHRF1* versus *hUHRF1; tp53*^*−/−*^, *P* value = 9.145 × 10^−7^. (**L**) Relative intensity of EGFP in each cell, normalized for each liver in *hUHRF1*; *tp53*^*−/−*^. Dots represent individual livers, and the number of biological replicates (clutches) is indicated. Data are presented as mean ± SD. *P value < 0.05 by unpaired *t* test. Exact *P* value for the annotated comparison is: *hUHRF1* versus *hUHRF1; tp53*^*−/−*^, *P* value = 0.0255. Scale bar: 10 μm. Source data are available online for this figure.

To better define the senescence phenotype caused by hUHRF1 overexpression, we assessed the expression of a senescence-associated gene set (Cellular Senescence in KEGG pathways) across hepatocyte populations which showed highest enrichment in Hep2 (Fig. 5A). Senescence-associated genes which were differentially expressed in *hUHRF1* bulk RNAseq (Fig. 3E) were enriched in Hep2 (Fig. 5B). In contrast, *tp53* and key downstream targets, *cdkn1a* and *mdm2*, were highly expressed in all hepatocyte populations, as was the key SASP regulator, *nfkb2*, and multiple DSB repair genes (Fig. 5B). This indicates that some markers of the senescence phenotype were present in all hepatocytes at this time point, but that there was a clear distinction in the transcriptomic profile of Hep2 cells. Interestingly, this population was depleted for genes associated with initiation and elongation of DNA replication (*mcm2*, *mcm6*, and *mcm7)*, but genes involved in DNA replication and DNA damage repair (*pcna* and *rrm2*) were upregulated (Fig. 5B). This suggests that while all cells are senescent at this time point, their expression profiles identify distinct senotypes, as found in other systems (Fernandez et al, 2025; preprint: Iwasaki et al, 2026; Kamat et al, 2025; Tao et al, 2024).

To determine whether the presence of dying cells could explain these diverse populations, we used the previously defined senescence score (Fig. 5A) to classify hepatocytes as positive or negative for the score (Appendix Fig. S4C; Table EV2). Next, we generated an apoptosis score (Table EV2) and assessed its association with senescence. Cells with high apoptosis scores were sparsely distributed across all hepatocyte populations (Appendix Fig. S4D,E), and apoptosis scores did not correlate with senescence scores (Appendix Fig. S4F). This suggests that hUHRF1 induces senescent cell phenotypes that are resistant to apoptosis due to expression of Bcl-2 family genes. We conclude that hUHRF1 overexpression induces cell death in a small population of cells that do not overlap with senescent cells.

### hUHRF1 levels correlate with senotypes

A recent study showed that Ras level determines the behavior and fate of senescent cells: cells with high Ras levels are immunogenic, leading to their elimination by the immune system, whereas cells with lower Ras levels are immunoresistant and can escape senescence (Chan et al, 2024). We hypothesized that different levels of hUHRF1 could correlate with different senotypes. To test this, hepatocytes from control and *hUHRF1* samples were segregated based on quantile expression of EGFP (Appendix Fig. S5). Cells with the lowest and highest 10% of EGFP expression were classified as low and top, respectively, and the remaining cells were assigned as mid (Fig. 5C,D). Nearly all the cells in controls livers expressed mid or low levels of EGFP, while cells in *hUHRF1* samples were heterogenous, with Hep2 having the highest proportion of top-expressing cells (Fig. 5D). Interestingly, *cdkn1a*, *tp53* and other senescence-associated genes were enriched in cells with top *hUHRF1* expression (Fig. 5E). The interaction between cell types was different based on *hUHRF1* expression, with the top and mid cells from Hep2 and Hep4 mostly engaged in crosstalk with each other, mesenchymal cells and macrophages while Hep3 and Hep1 had little interaction with any cell type (Fig. 5F). In contrast, Hep1, Hep3, and all hepatocyte populations with low *hUHRF1* expression showed no detectable cell–cell communication. This suggests that hUHRF1 levels determine distinct senotypes and could regulate their capacity to signal to neighboring cells.

To examine hUHRF1 heterogeneity and the relationship to cellular phenotypes in vivo, we used confocal imaging of livers expressing nuclear mCherry in hepatocytes *Tg(fabp10a:nls-mCherry)* together with *hUHRF1-EGFP* and scored each nucleus as positive or negative for either mCherry or EGFP (Fig. 5G,H). By 80 hpf most hepatocytes expressed nls-mCherry with minimal cell-to-cell variability (Appendix Fig. S6A), and by 120 hpf, the majority of hepatocytes were positive for both transgenes (Fig. 5G). However, the levels of EGFP were heterogeneous compared to mCherry, with significant variability from 1, which represents equal levels of EGFP and mCherry (Fig. 5H). In situ hybridization detected *hUHRF1* mRNA expression and hUHRF1-EGFP as similar (Appendix Fig. S6B), suggesting that EGFP serves as a proxy for the heterogeneous *hUHRF1* expression identified by scRNAseq.

Our previous finding that heterozygosity for *tp53* causes increased cell proliferation in early cancer stages and accelerated tumor formation in hUHRF1 animals (Mudbhary et al, 2014) suggested that proliferation in this model would be possible if cells had both acquired the ability to respond to proliferative signals and all cell cycle inhibitors were dismantled. To investigate whether Tp53 signaling acts as a gatekeeper that maintains senescence in cell populations susceptible to proliferative cues, we assessed EdU incorporation in *hUHRF1* livers with *tp53* and *atm* mutation and with iAtr inhibition at 96 hpf, as this was the stage with most profound difference in proliferation (Fig. 1C). However, despite effectively blocking SA-β-gal staining (Fig. 3A), neither *tp53* nor *atm* loss restored proliferation in *hUHRF1* samples (Fig. 5I). This indicates that at this stage of senescence, cell cycle arrest is maintained by factors acting upstream of *atm* and *tp53*, potentially as a direct consequence of UHRF1 overexpression.

We next reasoned that the effects of *tp53* loss on proliferation at later stages could be associated with distinct senescent phenotypes. Using hUHRF1-EGFP as a surrogate marker for the top-expressing cells identified in scRNAseq, we assessed EdU incorporation in the liver of *hUHRF1* larvae with, and without, functional *tp53* at 10 dpf (Fig. 5J). As expected at this precancer stage, some cells in *hUHRF1* livers with functional Tp53 showed an increased EdU incorporation (Mudbhary et al, 2014), with nearly all the EdU-positive cells lacking hUHRF1-EGFP (Fig. 5K-L). In *tp53*^*−/−*^ mutants, there was a threefold increase in the number of EGFP-positive cells that were also EdU positive (Fig. 5K). This indicates that Tp53 restrains proliferation in cells overexpressing hUHRF1, as suggested by the scRNAseq finding of active Tp53 signaling in cells with the highest *hUHRF1* expression (Fig. 5E). We examined whether hUHRF1 levels were correlated with proliferation by categorizing hUHRF1-EGFP levels in all cells as high, medium, and low based on expression quartiles (Fig. 5L) and then correlated this with EdU incorporation. We found that nearly all EdU-positive cells had low and medium EGFP in *hUHRF1* livers with WT *tp53*, whereas in *tp53* mutants, cells with medium levels of EGFP levels were proliferating. However, cells with the highest EGFP levels were EdU negative regardless of *tp53* status (Fig. 5L). Together, these data show that hUHRF1 levels correlate with distinct senescent phenotypes and capacities, some of which can respond to proliferative signals but are restrained by *tp53*, others which cannot proliferate, even when Tp53 is absent.

### Navitoclax reduces senescence and eliminates cells with high hUHRF1 levels

**Figure 6:**
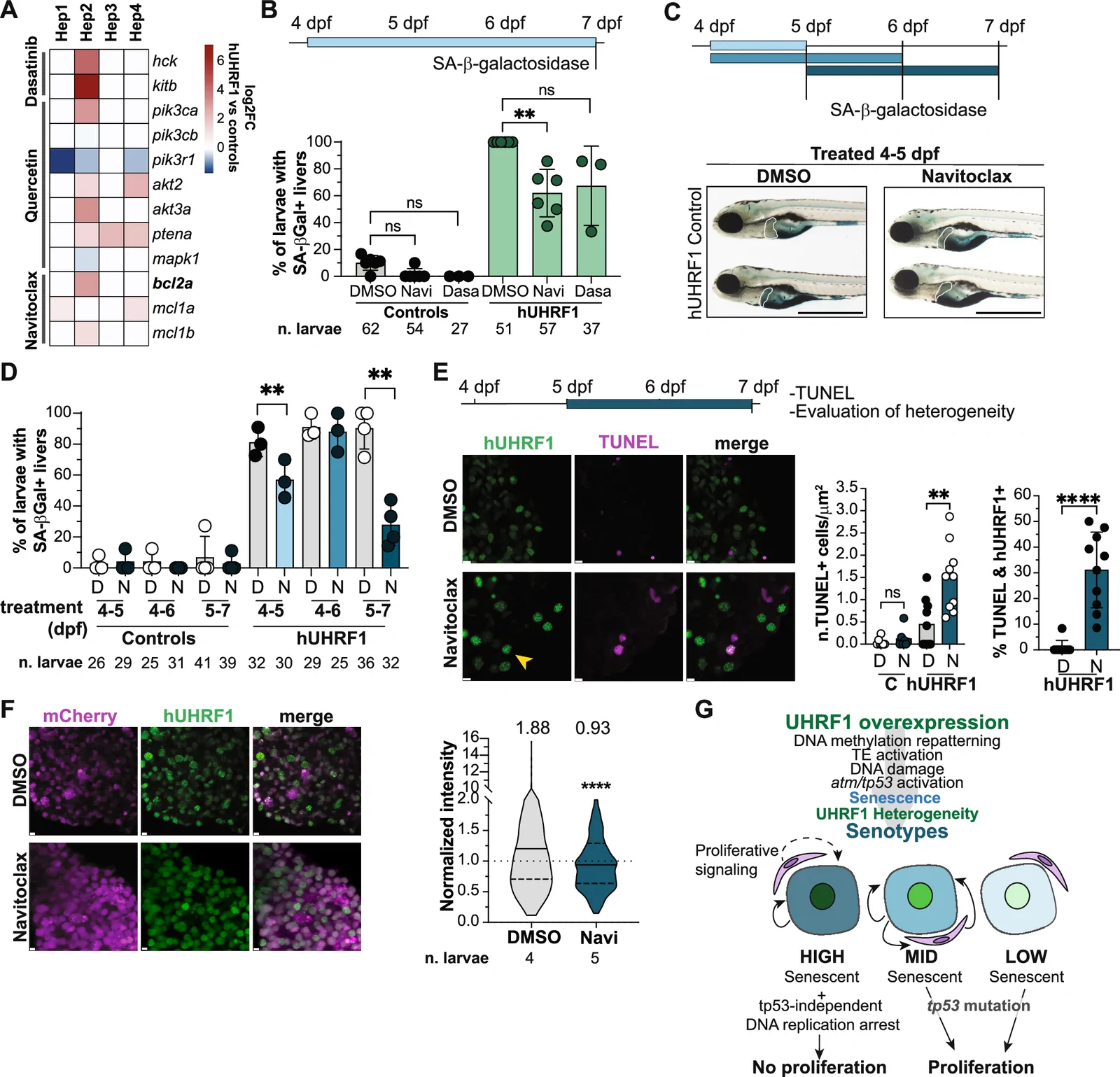
Navitoclax reduces senescence and eliminates cells with high hUHRF1 levels. (**A**) Heatmap of Log2 fold change of senolytic targets in *hUHRF1* hepatocytes compared to controls based on scRNAseq. (**B**) Percent of SA-β-gal-positive livers at 7 dpf in *hUHRF1* and control livers treated with Navitoclax (Navi), Dasatinib (Dasa), or DMSO (DMSO) from 4 until 7 dpf. Each dot represents biological replicates (clutches), and the number of larvae is indicated in the figure. Data are presented as mean ± SD. **P value < 0.01 by unpaired *t* test. Exact *P* value for the annotated comparison is: Navi versus DMSO in *hUHRF1*, *P* value = 0.0033. (**C**) Navitoclax treatment protocol. Representative images of SA-β-gal treated with DMSO or Navitoclax from 4 to 5 dpf. Scale bar: 1 mm. (**D**) Percent of SA-β-gal-positive livers in *hUHRF1* and control larvae treated with DMSO (**D**) or Navitoclax (N) as described in (**C**). Each dot represents biological replicates (clutches), and the number of larvae is indicated in the figure. Data are presented as mean ± SD. **P value < 0.01 by unpaired *t* test. Exact *P* value for the annotated comparison are: Navi versus DMSO in *hUHRF1* at 5 dpf, *P* value = 0.0057; Navi versus DMSO in *hUHRF1* at 5 dpf, *P* value = 0.0018. (**E**) Representative images of TUNEL staining of *hUHRF1* livers treated with DMSO or Navitoclax from 5 to 7 dpf. The yellow arrow indicates double-positive cells. The number of TUNEL-positive cells over the liver area (μm^2^) in controls (C) and *hUHRF1* treated with DMSO (D) or Navitoclax (N) is shown in the graph. The percent of cells that are positive for both TUNEL and EGFP cells in the livers of larvae treated with DMSO (D) or Navitoclax (N) is shown. Dots represent the number of livers in four clutches. Data are presented as mean ± SD. **P value < 0.01 and ****P value < 0.0001 by unpaired *t* test. Exact *P* value for the annotated comparison are: number of TUNEL-positive cells in Navi versus DMSO in *hUHRF1*, *P* value = 0.0024; number of TUNEL and hUHRF1 positive cells in Navi versus DMSO in *hUHRF1*, *P* value = 9.297 × 10^−5^. Scale bar: 5 μm. (**F**) Representative images of EGFP and nls-mCherry in the liver of larvae treated with DMSO or Navitoclax from 5 to 7 dpf. A violin plot represents the ratio of fluorescent intensity between EGFP and nls-mCherry in each cell, normalized to maximum intensity per liver for each marker. The solid line represents the median, and dashed lines indicate quartiles. The number on top of each violin indicates the standard deviation. The number of livers in two clutches is indicated. ****P value < 0.0001 by unpaired *t* test. Exact *P* value for the annotated comparison is: Navi versus DMSO, *P* value = 4.997 × 10^−10^. Scale bar: 5 μm. (**G**) Model of mechanism generating pleiotropic senotypes induced by *hUHRF1* overexpression. Source data are available online for this figure.

We next asked whether the hUHRF1-induced senotypes conferred differential sensitivity to senolytic therapy. Analysis of genes targeted by different senolytics showed most enrichment in Hep2 cells (Fig. 6A). This suggested that only a subset of senescent cell populations would be sensitive to senolytic targeting. To test this, *hUHRF1* larvae were treated from 4 to 7 dpf, the time course in which SA-β-gal develops, with concentrations of Navitoclax or Dasatinib that had no measurable effects on phenotype, and SA-β-gal staining was assessed. Navitoclax significantly reduced the percentage of larvae with SA-β-gal staining in the liver (Fig. 6B). Navitoclax added at 5 dpf (Fig. 6C) was even more effective at reducing SA-β-gal staining (Fig. 6D). We hypothesized that the cells expressing the highest levels of the Navitoclax target, *bcl2a* (Fig. 6A) and senescence signature genes (Fig. 5B) would be most susceptible to elimination. Navitoclax increased TUNEL staining in *hUHRF1* livers but not in controls (Fig. 6E). Importantly, TUNEL-positive cells in DMSO-treated *hUHRF1* samples were mostly negative for EGFP, but 30% of the TUNEL-positive cells were EGFP-positive in Navitoclax-treated livers (Fig. 6E). Since sample processing for the TUNEL assay diminished EGFP levels overall, we were unable to correlate hUHRF1-EGFP levels and apoptosis directly. Instead, we assessed which cells were present after Navitoclax treatment by comparing the levels of hUHRF1-EGFP to nls-mCherry in hepatocytes in Navitoclax and DMSO-treated *hUHRF1* samples. This showed that Navitoclax treatment significantly reduced hUHRF1-EGFP heterogeneity and cells with the highest level of hUHRF1-EGFP were no longer detected in Navitoclax-treated samples (Fig. 6F). Together, these data indicate that this senolytic is effective at removing some, but not all, senescent cells, sparing a cell population that can be prone to escape senescence and re-enter the cell cycle (Fig. 6G).

## Discussion

The cause, behavior, and fate of cells with different senotypes is an important, unanswered area in cancer biology. It is not clear how a single oncogene can induce diverse cell identities and fates. We address this using a precancer model in zebrafish whereby the epigenetic regulator and oncogene, UHRF1, is overexpressed in hepatocytes to first induce senescence and then later, develop into cancer (Mudbhary et al, 2014). We identified DNA damage, *atm*, and Tp53 activation as a mechanism of senescence caused by hUHRF1 overexpression, potentially mediated by DNA hypomethylation of TEs and the activation of LTR transposons. Moreover, we show that this response occurs within hours of UHRF1 overexpression, indicating that even a short-term disruption in UHRF1 expression can induce cancer-causing changes. The diversity of hepatocyte populations induced by hUHRF1 overexpression reflects diverse senotypes, which we hypothesize are driven by the widespread DNA methylation changes caused by deregulated UHRF1 levels, disrupting multiple pathways. We further show that different senescent cell phenotypes evolve, so that at early stages, senescence is sustained even if *atm* and *tp53* are removed. At later stages, however, a population of cells emerges. These have reduced UHRF1 levels and are receptive to proliferative signals and, if *tp53* is lost, can re-enter the cell cycle. In contrast, the population of cells with the highest levels of UHRF1 cannot proliferate even when Tp53 is removed. Importantly, our data suggest that senolytic therapy targets a subset of senescent cells, potentially leaving behind those with the ability to escape senescence, potentially contributing to cancer.

Senescence escape occurs when cells in a senescent state that retain the ability to reactivate proliferative genes lose key tumor-suppressive signals (Evangelou et al, 2023). Our finding that hepatocyte proliferation increases during later stages of the senescence-to-cancer transition suggests senescence escape. This is reminiscent of a mouse model of HCC, whereby Ras overexpression induced senescence, and the Ras-expressing cells were rapidly cleared by the immune system. However, when immune cells were depleted, Ras-expressing cells persisted and progressed to form liver cancer (Kang et al, 2011). A more recent study demonstrated that this is attributed to the level of Ras, which can act as a potent oncogene in HCC only when expressed at low levels, because hepatocytes with high Ras expression acquire an immunogenic senescence phenotype and are rapidly cleared. In contrast, cells with lower Ras expression have a senescence phenotype that is immune-evasive and escapable, leading to HCC (Chan et al, 2024). This phenomenon parallels our observations with UHRF1. High UHRF1 levels induce senescence, functioning as an effective tumor-suppressive barrier, and at the beginning of the senescence process, these cells remain non-proliferative even when the Atm-Tp53 pathway is inactivated. However, a subset of cells which have epigenetic alterations and DNA damage reduces hUHRF1 expression through an unknown mechanism or potentially had reduced hUHRF1 at the outset of the process and become proliferation competent.

It is not known how one stimulus provided by hUHRF1 can generate different senotypes, and we hypothesize that this is due to heterogeneity in UHRF1 levels. One possibility suggested by a study (Hsu et al, 2019) is that the cell cycle stage in which DNA damage occurs can influence subsequent cell behavior: cells with high levels of DNA damage in S and G2 phase rely primarily on ATR to activate TP53, while those in G1 with lower levels of DNA damage primarily rely on ATM. In this model, the G1 damaged cells transiently activate p21, resulting in subsequent proliferation, while those in S and G2 sustain high levels of p21, resulting in senescence (Hsu et al, 2019). Our data are consistent with this and suggest that UHRF1-induced senescence generates DNA damage in G1, as Atm is required for some senescent phenotypes in this model. Another possibility is that the senotypes generated by hUHRF1 reflect different hUHRF1 levels, with cells with the highest levels unable to proliferate while those with lower levels escape immune elimination and retain the ability to respond to proliferative cues, similar to what was found with Ras overexpression (Chan et al, 2024). While we do not know the cause of hUHRF1 heterogeneity in this model, it provides a useful system to study how hUHRF1 causes cancer, as there is also variable expression in human HCC (Mudbhary et al, 2014).

This model can be used to understand the mechanisms that repattern the epigenome in cancer. As DNA methylation changes occurring during oncogene-induced senescence are similar to those found in cancer (Cruickshanks et al, 2013; Hlady et al, 2017; Xie et al, 2018), it is likely that methylome changes are an early event in tumorigenesis. UHRF1 could be a key player in this important process, as it can change DNA methylation patterns during DNA replication either by titration or degradation of Dnmt1 (Bostick et al, 2007; Mudbhary et al, 2014; Qin et al, 2011; Shamma et al, 2013; Sharif et al, 2007), or by recruitment of Dnmt3a and repelling Tet2-mediated demethylation (Yamaguchi et al, 2024). Since loss of UHRF1 has been shown to be sufficient to induce diverse senescence phenotypes (Chen et al, 2025; Jung et al, 2017) it is possible that both loss and gain of UHRF1 can repattern the methylome, leading to diverse senotypes.

The study provides insight into the pleotropic nature of senescence and therapies targeting senescence. The finding that dramatic changes to the transcriptome, including TE activation, DNA damage, and widespread epigenetic changes, occur within 1 day of UHRF1 overexpression indicates that even a modest deregulation of UHRF1 can have dramatic, cancer-causing effects. In this early stage, therapies that remove all senescent cells may be advantageous for preventing cancer. However, as the precancer state evolves, approaches to selectively reinforce tumor-suppressive senescence while eliminating pro-tumorigenic senescent cells using senescent-promoting (senogenic) and senescent-eliminating (senolytic) drugs (Colucci et al, 2025) would be useful, albeit difficult to apply to achieve the desired outcome. To effectively develop and apply drugs to activate the anti-cancer senescent phenotypes and restrict those that promote the tumor ecosystem, a more nuanced understanding of the mechanisms by which diverse senescent states are generated, and their response to therapies, is needed. hUHRF1 overexpression in vivo provides a useful tool to address both questions. Delineating how senescence evolves into cancer in this model will be an important next step.

## Methods

### Zebrafish husbandry

Zebrafish (*Danio rerio*) care was conducted according to the New York University Abu Dhabi (NYUAD) for the Animal Care and Use Committee (IACUC) Committee (protocol number 22-0003A2). Adult fish were raised on a 14:10 h light: dark cycle at 28 °C and fed two times a day with artemia and once with Zeigler Zebrafish Diet. Larvae after 5 dpf were fed two times daily with paramecia. *Tg(fabp10a:hsa.UHRF1-EGFP*^*mss1*^*)*^*high*^ (Mudbhary et al, 2014) are hereafter called *hUHRF1*. Tg(*fabp10a:CAAX-EGFP)* (Nussbaum et al, 2013), Tg(*fabp10a:nls-mCherry)* (Mudbhary et al, 2014), or wild-type sibling (referred to as WT) of *hUHRF1* were used as controls. Tg(*mpeg1.1:dsRed2)* (see below), and *Tg(lyzC:dsRed)* (Hall et al, 2007) were used to identify macrophages and neutrophils, respectively. *tp53*^*−/−*^ (Berghmans et al, 2005) and *atm*^*−/−*^ (Ajouaou et al, 2023) mutant zebrafish were raised as incross and genotyped by PCR to identify homozygous *tp53*^*−/−*^ or genotyped by Sanger sequencing to identify homozygous *atm*^*−/−*^ mutants. *Tg(fabp10a:hsa.UHRF1-EGFP*^*mss1*^*), Tg(fabp10a:hsa.UHRF1-EGFP*^*mss1*^*);atm*^*−/−*^ or *Tg(fabp10a:hsa.UHRF1-EGFP*^*mss1*^*);tp53*^*−/−*^ were outcrossed to WT*, atm*^*−/−*^, or *tp53*^*−/−*^ fish respectively so that in all experiments, the *hUHRF1* allele was heterozygous. For experiments, 60 embryos were collected in 10 cm Petri dish and defined time points, larvae were screened for the presence of hUHRF1-EGFP under fluorescent stereomicroscope in hUHRF1 positive and hUHRF1-negative siblings (controls). The required number of larvae from controls and *hUHRF1* were randomly selected and used for the experiment. Each experiment has been performed on at least 3 independent biological replicates (clutches) and three individuals per clutch per group. The exact number of clutches and larvae is indicated in the figure and figure legends.

### Generation of a zebrafish transgenic line

*Tg(mpeg1.1:dsRed2)* fish expressing dsRed2 in macrophages was generated by PCR amplification of dsRed2 (Yoshinari et al, 2012). BamH1 (New England Biolabs) and MfeI (New England Biolabs) restriction enzyme sites were inserted with Q5® HotStart High-Fidelity DNA Polymerase (New England Biolabs) in the forward and reverse primers, respectively. A vector containing tol2 sites and mpeg1.1:Dendra2 cassettes (Addgene: #51462) was digested with the same restriction enzymes. After ligation and transformation in DH5alpha (Thermo Fisher Scientific), colonies were screened by PCR for the presence of dsRed2. Positive colonies were sequenced to avoid the presence of point mutations. One clone was selected and amplified by the GenElute Plasmid Miniprep kit (Sigma-Aldrich) and further purified with the MinElute PCR Purification Kit (Sigma-Aldrich). In total, 1 nl of a mixture prepared with 40 ng of plasmid and 80 ng of Tol2 transposase mRNA was injected into 1–2 cell stage embryos. Once embryos reached sexual maturity, they were crossed to WT fish to generate F1, and the founder with the brightest expression of dsRed2 in macrophages was selected to generate the stable line.

### Crispr/Cas9 deletion of human UHRF1

sgRNA targeting the human UHRF1 gene was produced by the sgRNA IVT kit (Takara Bio), the resulting RNA was isolated using Trizol (Invitrogen) and quantified by Qubit. The sgRNA was diluted to 50 ng/µL, mixed with an equal volume of previously diluted nls-Cas9 protein (IDT; 0.5 µl of nl-Cas9 added with 9.5 µL of 20 mM HEPES; 150 mM KCI, pH 7.5) and incubated at 37 °C for 5 min. In all, 1 nl of the injection mixture was injected into 1–2 cell stage embryos from an incross of *hUHRF1* and raised to 120 hpf. At 120 hpf, larvae were screened for the presence of EGFP in the liver in both injected and non-injected controls. EGFP-positive and negative cells from each group were used to perform SA-ß-galactosidase staining.

### Mapping *fabp10a:hUHRF1-EGFP* cassette integration into genome of *Tg(fabp10a:hUHRF1-EGFP)* transgenic zebrafish

Genomic DNA (gDNA) from hUHRF1-EGFP-positive zebrafish was extracted as described from 15 larvae derived from three independent biological replicates (clutches). In total, 5 µg of gDNA was digested with AluI (New England Biolabs) at 37 °C for 3 h. Digested DNA was purified with a PCR purification kit (Sigma-Aldrich) then ligated with T4 DNA ligase (New England Biolabs) at 16 °C overnight. Ligated products were PCR amplified with PCR followed by nested PCR by targeting the 5’ and 3’ region of the cassette, directed outside the cassette. Water was used as a PCR control, and WT gDNA was processed in parallel as a negative control. The PCR product was run on a 1% agarose gel, and the of interest band was gel-purified with the Gel Elute kit (Sigma-Aldrich). Purified products were sequenced and mapped to the zebrafish genome by nBLAST.

### In situ hybridization chain reaction

Larvae from *hUHRF1* and controls (*Tg(fabp10a:CAAX-EGFP)*) at 5 dpf were fixed in 4% paraformaldehyde overnight at 4 °C, washed three times with PBS, and gradually dehydrated in 100% methanol. After gradual rehydration in 100% PBS in situ HCR was performed as per the manufacturer’s instructions without modifications (Molecular Instruments). After washing, whole larvae were stained for 1 h at room temperature with Hoechst (1:2000 in PBS, Thermo Fisher Scientific) and washed three times with PBS, followed by mounting in 3% methylcellulose. Imaging was performed at the confocal microscope Stellaris.

### Drug treatment of zebrafish larvae

All treatments were performed on ten larvae from the control and ten larvae from *hUHRF1* in six-well plates containing 10 ml of embryo medium and incubated at 28 °C with 14:10 dark/light cycle according to standard protocols (Ramdas Nair et al, 2021). Atr inhibitor, VE-821 (ab219506, Abcam) was dissolved in DMSO (Sigma) at 20 mM stock solution and stored at −20 °C in single-use aliquots. Larvae were treated with 10 μM VE-821 or 0.05% DMSO from 72 to 96 or 120 hpf when larvae were collected for EdU or SA-β-gal staining, respectively. Dasatinib (Sigma) was resuspended in DMSO to prepare 20 mM stock and Navitoclax (Sigma) was resuspended in DMSO to prepare a 10 mM and stored at −20 °C in single-use aliquots. Larvae were treated from 4 to 7 dpf or 5 to 7 dpf as described in the figures with a final concentration of 2.5 μM Dasatinib or with 25 μM Navitoclax in 1% DMSO. DMSO 1% was used for the DMSO-treated. At 7 dpf, larvae were collected for SA-β-gal staining, TUNEL, and heterogeneity analysis.

### EdU incorporation

*hUHRF1* and control larvae at the indicated time points were incubated in a 50 ml tube with 250 μl of embryo medium containing 10% DMSO and 0.5 mM EdU (Thermo Fisher Scientific, prepared in embryo water) for 20 min on ice. After 20 min, 50 ml of embryo medium was added to the larvae and then incubated at 28 °C for 30 min. For 10 dpf larvae, 0.22-μm filtered system water was used instead of embryo medium for the EdU incorporation. After EdU, larvae were fixed with 4% paraformaldehyde overnight at 4 °C, washed once with PBS, and gradually dehydrated in 100% methanol and stored at 4 °C overnight. EdU pulsed larvae were stained by Click-IT (Thermo Fisher Scientific) as described (Madakashira et al, 2024; Magnani et al, 2024). Briefly, larvae were gradually rehydrated in 100% PBS and permeabilized for 30 min in PBS containing 1% Triton-X and 20 μg/ml of Proteinase K (Machery-Nagel). After permeabilization, larvae were washed three times with PBS for 5 min and incubated twice with PBS containing 8 mM CuSO_4_ (Sigma-Aldrich), 4 nM Sodium Azide (A555 or A647, Thermo Fisher Scientific), and 50 mM of freshly prepared Ascorbic Acid (Sigma-Aldrich) for 30 min at room temperature. Livers were dissected, stained with Hoechst (1:2000, Thermo Fisher Scientific) and GFP booster (1:1000, Chromotek) for 1 h at room temperature and washed three times in PBS before mounting them in 3% methylcellulose. A z-stack through each liver (*z* = 0.33 mm) was acquired at Leica Stellaris 8 Confocal Microscope using the Lightning function. Images were quantified by Imaris using the Spot function to count Hoechst, EGFP, and EdU-positive cells. To determine the level of EGFP in the EdU-positive cells, the intensity of EGFP in EdU-positive cells that were identified by the Spot function was measured in pixels. For each liver, the pixel intensity of the EGFP signal for the three brightest EGFP-positive cells, regardless of EdU status, was measured and used to normalize the EGFP signal to set the top level. For each time point and condition, at least three independent biological replicates were used.

### Immunofluorescence

*hUHRF1* and control larvae from the indicated time points were collected and fixed with 4% paraformaldehyde at 4 °C overnight and processed as described (Madakashira et al, 2024). Briefly, larvae were washed twice with PBS, livers were manually dissected and incubated with PBS containing 1% Triton-X (Sigma-Aldrich) and 20 μg/ml of Proteinase K (Machery-Nagel) for 10 min at room temperature then washed with PBS and incubated for 1 h at room temperature with blocking solution (PBS containing 2% bovine serum albumin (Sigma-Aldrich)) followed by incubation at 4 °C overnight with primary antibody solution (1:100 antibody in blocking solution). After primary antibody staining, livers were washed three times with PBS containing 0.1% Tween-20 and two washes with PBS, followed by incubation with secondary antibody solution (blocking solution with 1:300 secondary antibody and 1:2000 Hoechst, Thermo Fisher Scientific) for 1 h at room temperature. After washing with PBS, livers were mounted with Vectashield (Vector) and imaged on a Leica Stellaris 8 Confocal Microscope and quantified with LasX software. Primary antibodies were γH2AX (gtx127342, Genetex) and p-RPA (ab211877, AbCam) and used at 1:100. Secondary antibodies were anti-rabbit Alexa 555 (Thermo Fisher Scientific), anti-rabbit Alexa 647 (Thermo Fisher Scientific), and used at 1:300. At least three livers were imaged for each experiment in at least 2 clutches.

### Analysis of immune cell recruitment

Macrophages and neutrophils were imaged using *Tg(mpeg1.1:dsRed)* and *Tg(lyzc:dsRed)*, respectively, using a Nikon Stereomicroscope. For macrophages, the left liver lobe was traced, and the total pixel intensity of dsRed2 in the liver area was measured using ImageJ. Fluorescence intensity was normalized by liver area for each larva. For neutrophils, the liver area was measured in µm^2^ in ImageJ, and the number of dsRed spots corresponding to the number of neutrophils was counted manually. The number of neutrophils was normalized on the liver area for each larva.

### Analysis of EGFP fluorescence heterogeneity

*hUHRF1* and nls-mCherry lines were crossed, and *hUHRF1;nls-mCherry* or *nls-mCherry* larvae were collected at the indicated time points, fixed in 4% paraformaldehyde at 4 °C overnight, and then transferred to PBS. Livers were manually dissected, permeabilized with PBS containing 1% Triton-X (Sigma-Aldrich) and 20 μg/ml Proteinase K (Machery-Nagel) for 10 min at room temperature, and stained with 1:2000 Hoechst (Thermo Fisher Scientific) in PBS for 1 h at room temperature. Livers were washed three times with PBS and were mounted on a glass slide with Vectashield (Vector). Images were acquired with Leica Stellaris 8 Confocal Microscope. For each liver, the laser power was set to have 1–2 saturated cells for each channel. Image analysis was performed with Fiji by creating a mask on the Hoechst channel and then quantifying the intensity of mCherry and EGFP for each cell present in the mask. For each image and for each channel, intensity levels of each cell were normalized based on the cell with the highest intensity in that image, and the ratio between normalized EGFP intensity and normalized mCherry intensity was plotted for visualization in GraphPad Prism 8. At least three livers were imaged for each experiment in at least three clutches.

### SA-β-gal staining

SA-β-gal staining was performed using the Senescence beta-galactosidase staining kit (Cell Signaling Technologies). Briefly, *hUHRF1* or control larvae at indicated time points were collected and fixed with fixing solution for 15 min at room temperature, followed by three washes with PBS pH 7.4. Staining was performed overnight at 37 °C with staining solution with optimized pH at 6.2 (Magnani et al, 2024). Larvae were washed in PBS and mounted in 3% methylcellulose for imaging at a stereoscope. SA-β-gal staining in the posterior gut was considered a positive control, and larvae were scored blindly for the presence of staining in the liver and were assigned a categorical score as positive or negative.

### TUNEL assay

TUNEL assay was performed as previously described (Magnani et al, 2024). Briefly, at the indicated time point, larvae were fixed in 4% PFA overnight at 4 °C. After fixation, larvae were washed three times with PBS and gradually dehydrated in 100% methanol, incubated at 4 °C overnight, and gradually rehydrated in PBS. Livers were manually dissected, permeabilized for 10 min at room temperature in PBS containing 0.1% sodium citrate, 0.1% Triton-X, 20 μg/mL of Proteinase K, and then washed three times in PBS and post-fixed in 4% PFA for 10 min. TUNEL solution was prepared according to the manufacturer’s instructions (In Situ Cell Death Detection Kit, TMR, Roche), added to the livers and incubated 1 h at 37 °C in the dark, followed by washing three times in PBS and mounted on a glass slide with DAPI-containing Vectashield (Vector). Images were acquired with a Leica Stellaris 8 Confocal Microscope. TUNEL-positive cells were counted using the spot function in Imaris, and liver area was measured in Fiji as an ROI and converted into μm^2^ based on the scale bar.

### Protein extraction and western blot

For protein extraction, 50 livers from *hUHRF1* and controls were manually dissected and collected in ice-cold PBS. After centrifugation, livers were resuspended in RIPA buffer (Sigma-Aldrich, 1 μl of RIPA buffer per liver) supplemented with protease inhibitors cocktail (Roche), incubated 15 min of ice, and sonicated to release proteins with a Hand sonicator (Hielscher Ultrasonics GmbH). Protein lysates were cleared by centrifugation at top speed for 10 min. Protein lysate was supplemented with Laemmli buffer (Bio-Rad), denatured for 5 min at 95 °C, loaded into acrylamide/bisacrylamide denaturing gel (Sigma-Aldrich), and run at constant 80 V. 10% and 15% acrylammide/bisacrylamide gels were used for Tp53 and Histone H3 analysis, respectively. Proteins were transferred onto PDVF membrane (Thermo Scientific) for 1.5 h at a constant 300 mA on ice. Membrane was blocked for 1 h at room temperature with TBS (Bio-Rad) supplemented with 0.1% Tween-20 (Sigma-Aldrich) containing 5% skim milk (Sigma-Aldrich), incubated overnight at 4 °C with 1:1000 primary antibody diluted in blocking solution (Tp53, Genetex; H3, Santa Cruz), followed by secondary antibody diluted 1:2500 in blocking solution (HRP-conjugated anti-Rabbit, Promega). Chemiluminescence was revealed with ECL Clarity (Bio-Rad) and imaged with ChemiDoc MP Imaging System (Bio-Rad). Images were quantified with GelAnalyzer.

### RNA extraction, library preparation, and bulk RNAseq analysis

Pools of 20–30 manually dissected livers from five independent clutches of *hUHRF1* and control larvae at 80 hpf or 10–15 livers at 120 hpf were used for total RNA isolation. Livers were collected directly in 500 μl of Trizol (Thermo Fisher Scientific). RNA was extracted by following the manufacturer’s instructions with some modifications. After chloroform, RNA was precipitated in isopropanol (Sigma-Aldrich) with 10 μg of Glycoblue (Thermo Fisher Scientific) overnight at −20 °C. After precipitation, RNA was centrifuged at 4 °C for 1 h, washed in 70% ethanol, and resuspended in 20 μl of DNAse/RNAse-free water. Possible genomic DNA contamination was removed with RapidOut DNA removal Kit (Thermo Fisher Scientific) for 30 min at 37 °C following the manufacturer’s instructions.

After quality control with Bioanalyzer (Agilent), 100 ng of RNA was used for library preparation with ILMN Stranded Total RNA Lig w/RBZ+ (Illumina). Libraries were sequenced on the NovaSeq 6000 Illumina platform. Quality of the sequences was assessed by using MultiQC v1.0 (https://multiqc.info). After adapter removal and trimming, reads were aligned to the *D. rerio* GRCz10 reference genome, with manual insertion of human UHRF1 and EGFP sequence, using HISAT2 with default parameters (Kim et al, 2019), and mapped and counted with HTSeq (Anders et al, 2015). Differential gene expression was calculated using a generalized linear model implemented in DESeq2 in Bioconductor (Love et al, 2014) to test differential gene expression between *hUHRF1* transgenic livers and WT sibling controls or, in the case of *tp53*^*−/−*^ mutants, normalized to *tp53*^*−/−*^ samples. Adjusted *P* value with a false discovery rate of <0.05 was used to determine DEGs.

Quantification of repetitive elements was assessed by the SQuIRE workflow (Yang et al, 2019) to quantify expression at the subfamily level and properly assign multi-mappers sequences (due to the nature of repetitive elements) using expectation–maximization algorithm. In brief, trimmed fastq were aligned to *D. rerio* GRCz10 reference genome using STAR. Repetitive Elements (REs) annotation from RepeatMasker was used for alignment with unique mapping reads aligned to a single locus and marking multi-mapped reads to multiple locations, and further refined with the EM algorithm with “auto” parameter. Total counts (unique and multi-mapped) are aggregated by subfamily of REs, and the output count table from SQuIRE has been analyzed by DEseq2 with standard parameters to calculate differential expression of REs between conditions.

For gene ontology analysis, DEG (*P* value adjusted <0.05) at 80 hpf and 120 hpf were used to perform Ingenuity Pathways Analysis (Qiagen), and the top induced pathways at 80 and 120 hpf were plotted using Graph Prism 8. Heatmaps were generated with Pheatmap on Log2FC of *hUHRF1* compared to controls and plotted in R.

### DNA extraction, RRBS, and DNA methylation analysis

Genomic DNA was extracted from 80-100 livers dissected from three independent clutches of *hUHRF1* or Tg(fabp10a:CAAX-EGFP) larvae as controls using a DNA extraction buffer (10 mM Tris-HCl pH 9, 10 mM EDTA, 200 mM NaCl, 0.5% DSD, 200 μg/ml proteinase K). DNA was resuspended in water and quantified by the Qubit dsDNA High Sensitivity kit. RRBS was performed on 80 ng of genomic DNA and digested with 200 U of MspI for 24 h (New England Biolabs) and used for library preparation with minor modifications on a previously published protocol (Magnani et al, 2021) (Garrett-Bakelman et al, 2015). To avoid loss of gDNA, after MspI digestion, end repair, and A-tailing, the reactions were stopped by heat inactivation. The adapters used for multiplexing were purchased separately (Next Multiplex Methylated Adapters-New England Biolabs). Libraries were size-selected for fragments from 175 bp to 670 bp by dual-step purification with Ampure XP Magnetic Beads (Beckman Coulter). Bisulfite conversion was performed with Lightning Methylation Kit (ZYMO Research) following the manufacturer’s instructions. Libraries were amplified using KAPA HiFi HotStart Uracil+ Taq polymerase (Roche) and purified with Ampure XP Magnetic Beads (Beckman Coulter) before sequencing. Libraries were sequenced as 150 bp paired-end reads on the Illumina Nextseq 600 platform. Quality control of the RRBS sequencing data was assessed using FASTQC and Trimmomatic and aligned to the reference genome GRCz10 (Bolger et al, 2014).

RRBS data were analyzed for CpG methylation levels using the R package “methylKit” (Akalin et al, 2012). CpGs covered at least ten times in one biological replicate were included in the analysis. CpGs with a *q* value indicating a false discovery rate of <0.01 were considered differentially methylated, and they were segregated between those that had a methylation change of greater or less than 25% in hUHRF1 samples compared to controls. For plotting and statistical analysis, R package “pheatmap” and GraphPad Prism software were used.

### Preparation of single-cell suspension and scRNAseq

In total, 150–800 livers at 5 dpf from controls and *hUHRF1* were manually dissected and put in ice-cold PBS containing 2% FBS (Gibco) and 1% penicillin/streptomycin (Gibco). After dissection, livers were briefly centrifuged at 200×* g* for 3 min, resuspended in 1 ml of Trypsin/EDTA 0.05% (Sigma-Aldrich), and incubated at 28 °C for 12–20 min. Every 2 min, livers were pipetted ten times to facilitate cell dissociation until all the cells resulted in a homogenous suspension. Overall, 200 μl of FBS (Gibco) was added to inactivate Trypsin, and cells were centrifuged at 4 °C for 7 min at 500 g. After centrifugation, cells were washed three times with PBS containing 0.4% BSA (Sigma-Aldrich). Cells were resuspended in 50–100 μl of PBS containing 0.4% BSA and counted with a Burker Chamber. Cell suspension at a concentration of 600–700 cells/μl was used to load the Chip for 10X Genomics acquisition. All steps of single-cell sequencing were performed following the Chromium Next GEM Single Cell 3ʹ Reagent Kits v3.1 (Dual Index).

### Single-cell RNAseq analysis

scRNAseq data were processed and filtered using Seurat v5.0.1 (Hao et al, 2024). Initial quality control and filtering of scRNAseq data were performed to ensure the retention of high-quality cells. In that line, cells with fewer than 200 detected features and nCount_RNA or mitochondrial gene content greater than 20% were excluded from downstream analysis. Data filtering resulted in 12,202 cells for controls and 10,919 cells for *hUHRF1*. Highly variable features were identified using the “vst” method, with the top 2000 features selected. Data integration was performed with the Seurat package using the FindIntegrationAnchors function with canonical correlation analysis (CCA) (Stuart et al, 2019). Dimensionality reduction and clustering of scRNAseq data were performed using principal component analysis (PCA), retaining the top 19 principal components. Uniform Manifold Approximation and Projection (UMAP) dimensionality reduction was applied using these components for visualization. Neighbor identification was conducted with FindNeighbors (k = 20), followed by clustering using FindClusters with a resolution of 0.3. DEGs in each cell cluster were identified using the FindMarkers function, and statistically significant markers between compared groups were selected based on an adjusted *P* value threshold (*P* value < 0.05) were used to perform gene ontology. All dimensionality reduction and DEG analyses were conducted using the Seurat package.

To define differences between populations, the ClusterProfiler package was used in R. Significantly enriched markers (*P* value adjusted <0.05 & Log2FC > 0.5) were used to perform Gene Ontology. The three most significant GO terms were plotted by the dotplot function in ClusterProfiler.

To define the differences between *hUHRF1* and controls, differential gene expression was performed on *hUHRF1* compared to controls for each population. DEGs (*P* value adjusted <0.05 & Log2FC of 1.5) in each hepatocyte population were used to perform KEEG pathway analysis using ClusterProfiler package in R. The five most significant KEEG terms were plotted using the dotplot function in ClusterProfiler. To identify high, mid, and low expressing cells, hepatocyte populations in *hUHRF1* and controls were selected, and quantiles of EGFP were defined. Cut-off of EGFP separating the 10% most expressing cells (HIGH) and 10% least expressing cells (LOW) were selected, and MID cells were defined as cells expressing the middle 11–89th percentile of EGFP levels. DEGs were identified using the FindMarkers function on High, Low, and Mid cells downsampled (seed 123) to match the number of cells in High and Low (1416 cells) and separated into controls and *hUHRF1*.

### Cell–cell communication analysis

Cell–cell communication was inferred at single-cell resolution using the CellChat R package (v2.1.2) following the standard workflow (Jin et al, 2021). Ligand-receptor interactions from secreted signaling were mapped using the zebrafish-specific CellChatDB.zebrafish database. Communication probabilities were estimated with *computeCommunProb*, and interaction pairs represented by fewer than ten cells were excluded. Pathway-level signaling probabilities were obtained with *computeCommunProbPathway* function and summarized using *aggregateNet*. Interaction numbers and strengths were visualized as chord diagrams using *netVisual_chord_cell*. Outgoing and incoming signaling roles were quantified with *netAnalysis_computeCentrality* and displayed using *netAnalysis_signalingRole_heatmap*.

### Statistical analysis

Statistical analysis was performed in GraphPad Prism 8. At least three biological replicates were used for each experiment. Methods to evaluate the statistical significance include two-tailed Student’s *t* test with adjustment for multiple comparisons, *t* test, or Chi-square for categorical variables. Tests used are indicated in the figure legend. All the plots were generated in GraphPad Prism 8 and RStudio 3.3.1.

## Supplementary information

Table EV1

Table EV2

Appendix

Peer Review File

Data Set EV1

Data Set EV2

Data Set EV3

Source data Fig. 1

Source data Fig. 2

Source data Fig. 3

Source data Fig. 5

Source data Fig. 6

Expanded View Figures

## Data Availability

Raw data (fastq files) and processed files (raw counts) for bulk RNAseq have been deposited in GEO as GEO: GSE302812. RRBS data have been deposited in GEO in GEO: GSE173792. Raw data (fastq files) and processed files (Cell Ranger output) for single-cell RNAseq have been deposited in GEO as GSE303361. This paper does not report original code. The source data of this paper are collected in the following database record: biostudies:S-SCDT-10_1038-S44319-026-00941-y.
