# Supplementary material for: UHRF1 overexpression generates distinct senescent states with different Tp53 dependencies
Source: EMBO Rep. 2026 Sep 28;27(21):6425–46. doi: 10.1038/s44319-026-00941-y (PMC13743750; doi:10.1038/s44319-026-00941-y)
Supplement: Supplementary file 1 [file 44319_2026_941_MOESM13_ESM.pdf]

Expanded View Figures

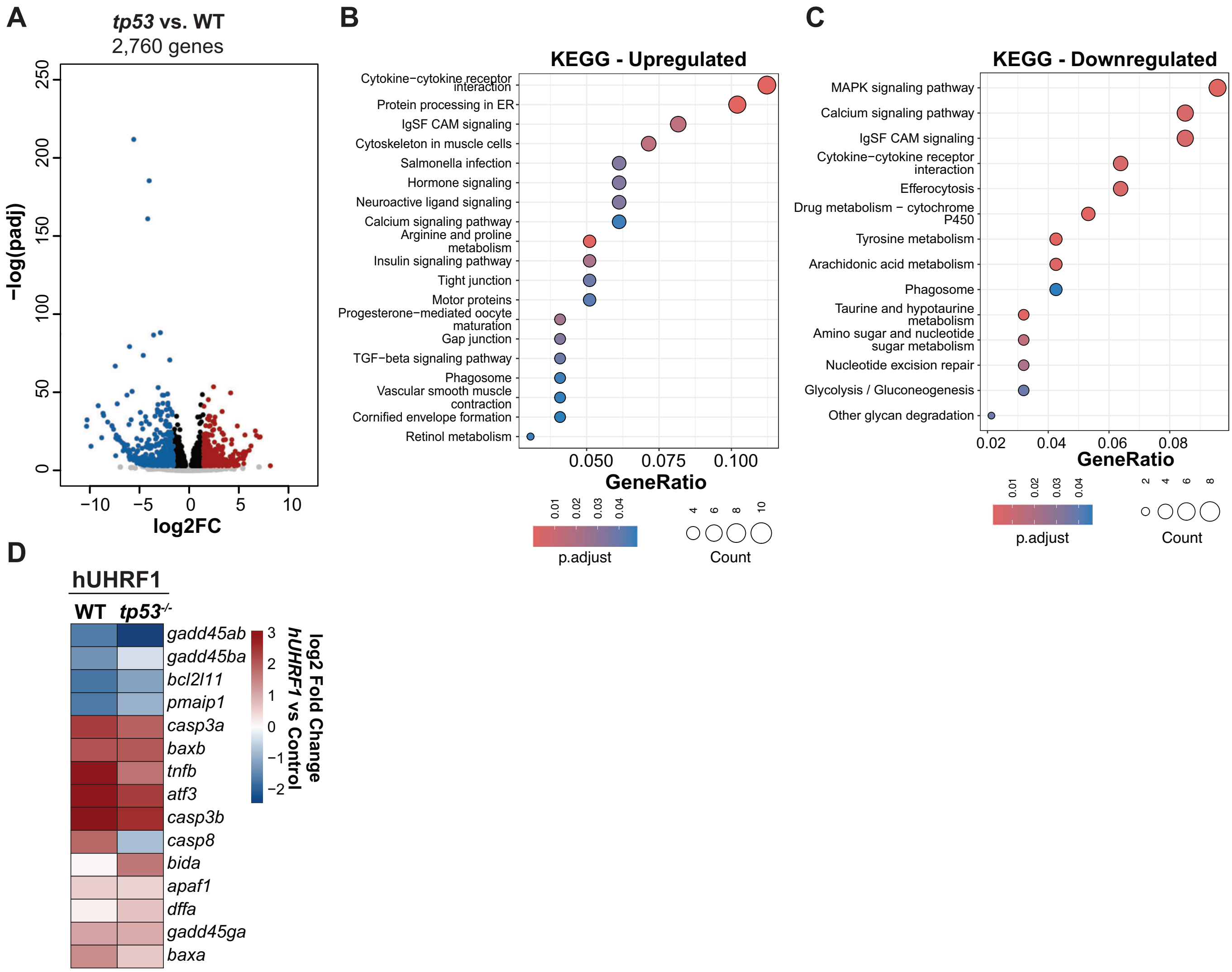

Figure EV1. Bulk RNAseq shows many differentially expressed genes in the liver of *tp53*$^{-/-}$ mutants.

(**A**) Volcano plot of differentially expressed genes in the liver of *tp53*$^{-/-}$ larvae compared to wild-type at 120 hpf in five biological replicates. Gray dots indicate genes with *P* value adjusted >0.05, black dots indicate genes with *P* value adjusted <0.05, red dots indicate genes with *P* value adjusted <0.05 and log2 fold change >1.5, and blue dots indicate genes with *P* value adjusted <0.05 and log2 fold change < −1.5. *P* values were calculated using the Wald test implemented in DESeq2 and adjusted for multiple testing using the Benjamini–Hochberg method. Genes with an adjusted *P* < 0.05 were considered significantly differentially expressed. (**B**) KEEG pathway analysis of upregulated genes (*P* value adjusted <0.05 and log2 fold change >0). KEGG pathways enrichment was assessed using a one-sided hypergeometric test with Benjamini–Hochberg correction for multiple testing. Dot size represents the gene ratio, and color represents −log10 of the adjusted *P* value. (**C**) KEEG pathway analysis of downregulated genes (*P* value adjusted <0.05 and log2 fold change <0). KEGG pathways enrichment was assessed using a one-sided hypergeometric test with Benjamini–Hochberg correction for multiple testing. Dot size represents the gene ratio, and color represents −log10 of the adjusted *P* value. (**D**) Heatmap of log2 fold change of genes involved in apoptosis in samples overexpressing hUHRF1, comparing *hUHRF1* to WT (WT) and *hUHRF1;tp53*$^{-/-}$ to *tp53*$^{-/-}$(*tp53*$^{-/-}$).

© The Author(s)

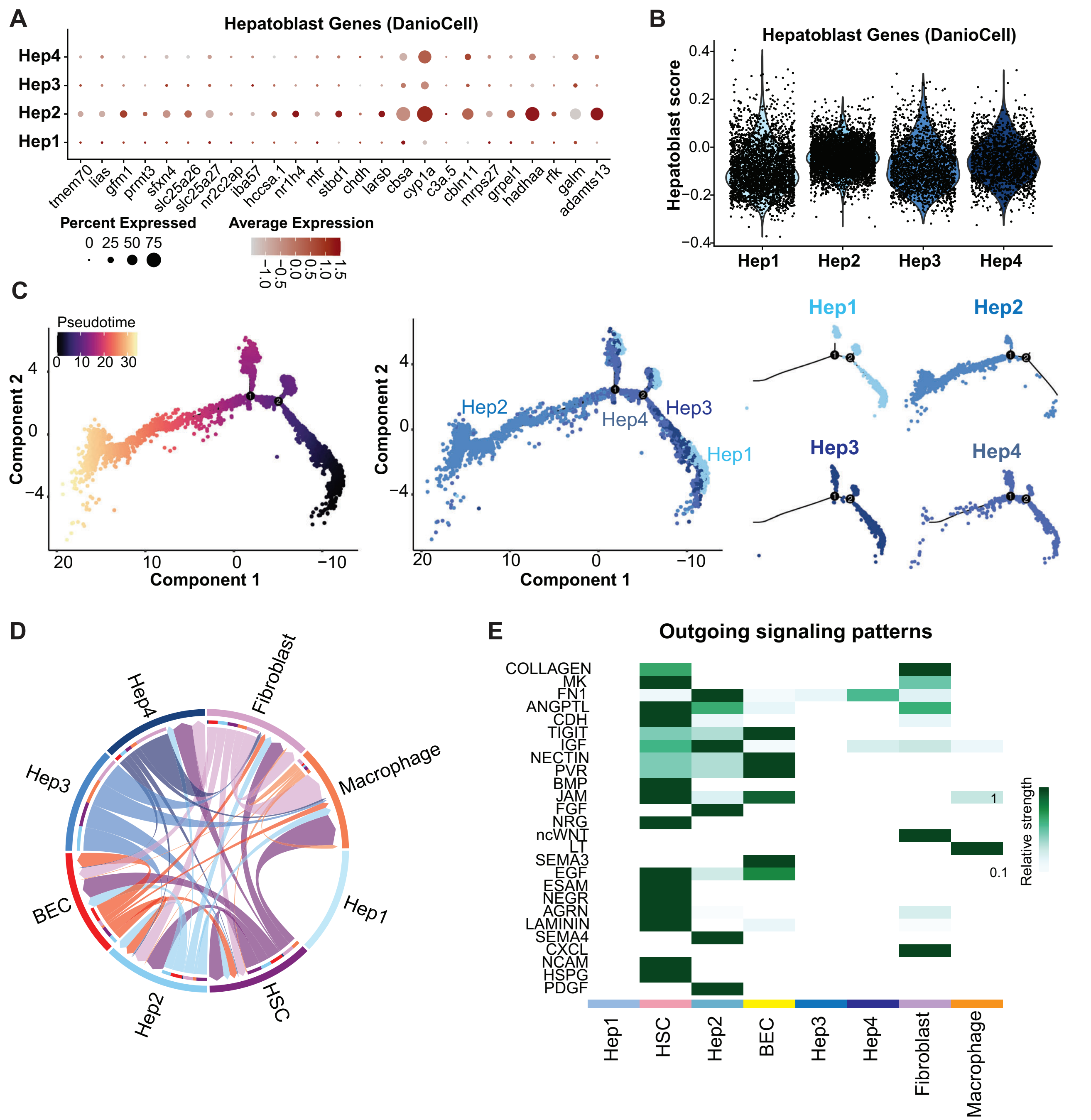

Figure EV2. Hepatocytes in control larvae cluster based on expression profile reflecting maturation.

(**A**) Expression of hepatoblast genes obtained from DanioCell in hepatocytes from control larvae at 120 hpf. (**B**) Hepatoblast Score of genes present in A in Hep1-4 in controls graphed in individual cells identified from scRNAseq. (**C**) Pseudotime projection of hepatocytes in controls. (**D**) Chord diagram showing cell-cell communication in control, with the width of each chord representing the proportion of cells with that signal and the arrows representing the direction of the signal. (**E**) The top outgoing signaling molecules in control are inferred based on the relative strength of the signaling.

© The Author(s)
