# Supplementary material for: UHRF1 overexpression generates distinct senescent states with different Tp53 dependencies
Source: EMBO Rep. 2026 Sep 28;27(21):6425–46. doi: 10.1038/s44319-026-00941-y (PMC13743750; doi:10.1038/s44319-026-00941-y)
Supplement: Supplementary file 2 [file 44319_2026_941_MOESM3_ESM.pdf]

Appendix Files

Table of content

Appendix Figures……………………………………………………………………2-7

Appendix Figure S1…………………………………………………………………..2

Appendix Figure S2…………………………………………………………………..4

Appendix Figure S3…………………………………………………………………..5

Appendix Figure S4…………………………………………………………………..6

Appendix Figure S5…………………………………………………………………..8

Appendix Figure S6…………………………………………………………………..9

Appendix Figure S1

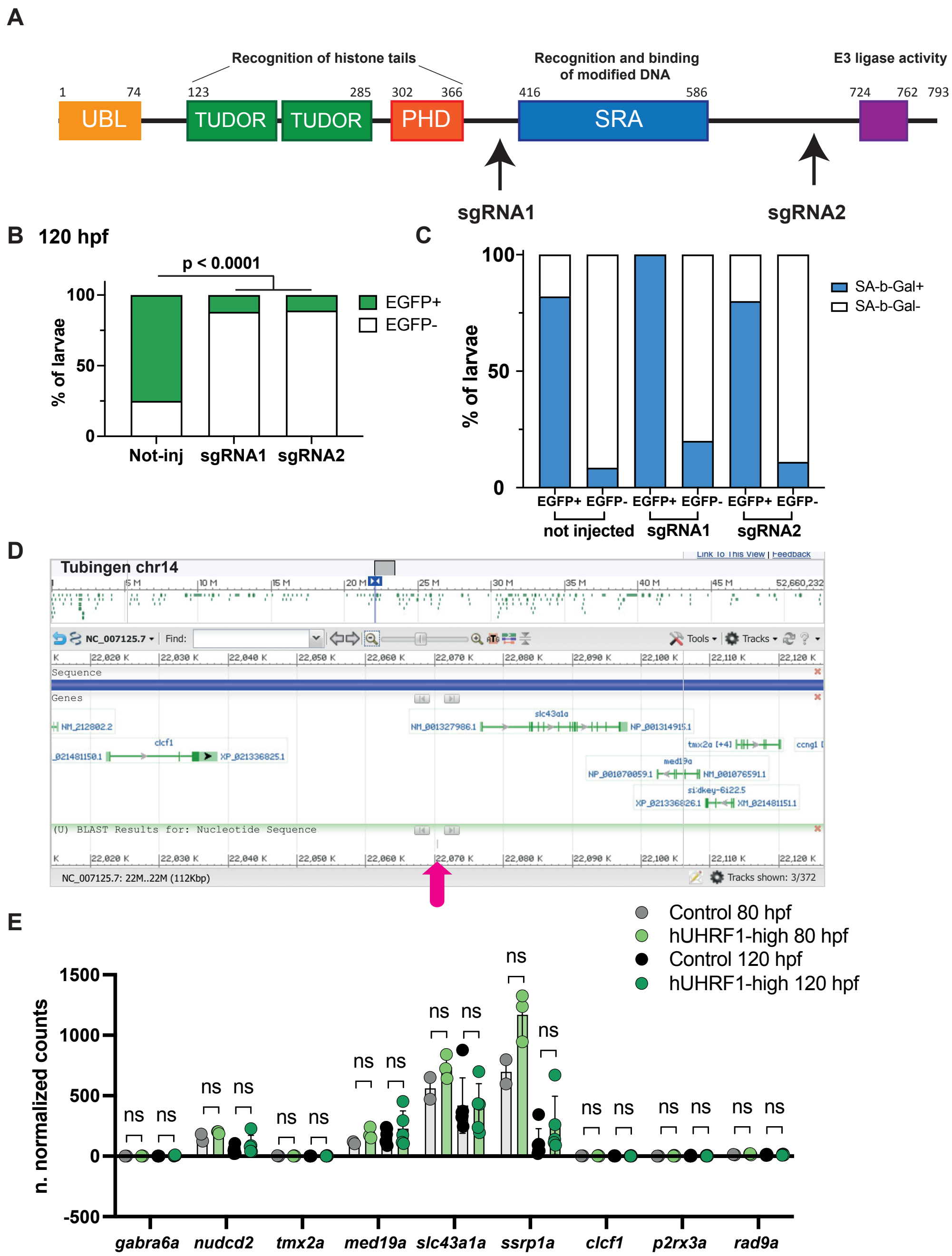

Appendix Figure S1. Senescence in the hUHRF1 is independent of the transgene insertion site. A. Schematic representation of hUHRF1, its domains and the region targeted by sgRNAs. **B.** Percent of larvae with EGFP expression as a marker of hUHRF1-EGFP mutation. Data are presented as mean of 3 biological replicates (clutches). **** p-value < 0.001 calculated by Chi-square test with the ratio in the non-injected sample considered the expected ratio. Exact p-value

for the annotated comparison are: sgRNA1 versus non-injected, p-value = $4.642X10^{-18}$; sgRNA2 versus non-injected, p-value = $1.843X10^{-20}$. **C.** SA-β-gal staining of non-injected and injected larvae separated by EGFP presence. Data are presented as mean of 3 biological replicates (clutches). **D.** Chromosomal view of hUHRF1 insertion. Pink arrow indicates the site of insertion of the fabp10a:hUHRF1-EGFP cassette into the genome of *Tg(fabp10a:hUHRF1-EGFP)* zebrafish line obtained in one experiment performed on a pool of 15 larvae derived from 3 different biological replicates (clutches) pooled. **E.** Normalized counts of genes flanking the insertion site at 80 and 120 hpf measured by DeSeq2 analysis of bulkRNAseq from 3 biological replicates of *hUHRF1* compared 2 biological replicates of Control livers at 80 and 6 biological replicates of *hUHRF1* compared 6 biological replicates of Control livers at 120 hpf. Data are presented as mean ± SD. Significance is measured by un-paired t-test. P-value > 0.05 is considered non-significant.

Appendix Figure S2

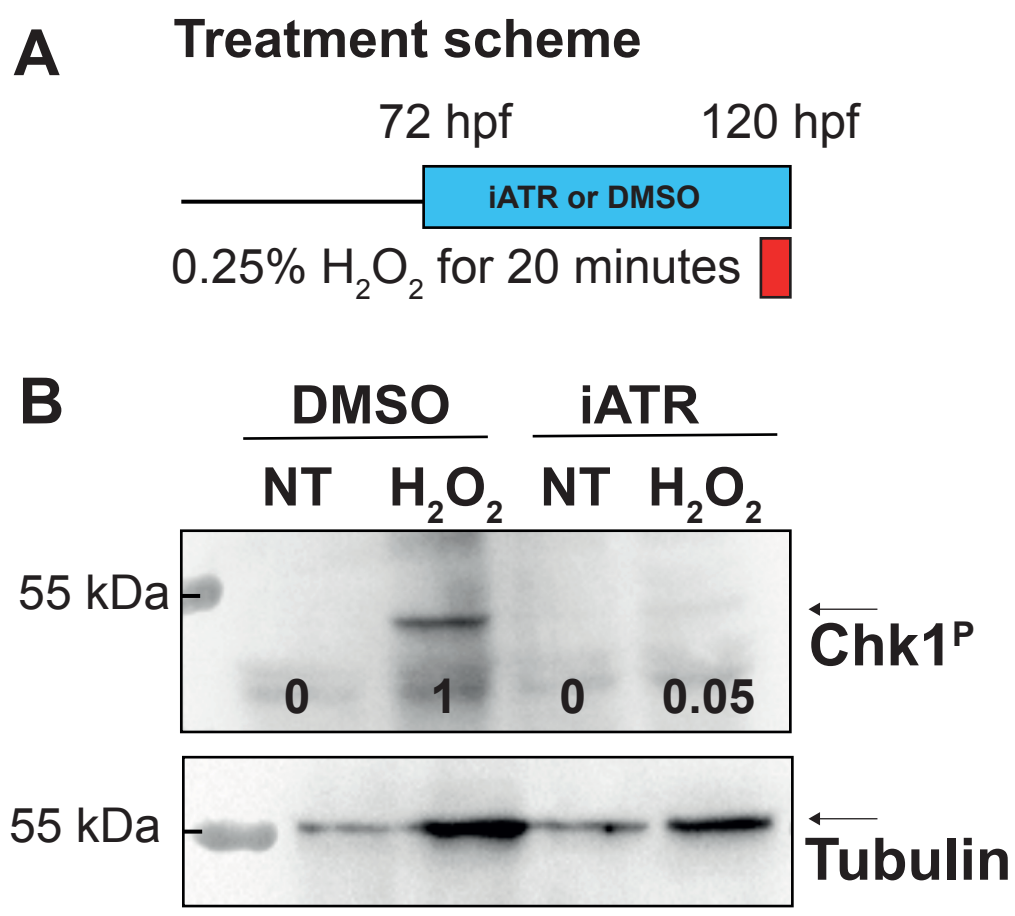

Appendix Figure S2. Validation of VE-821 as Atr inhibitor block. A. Treatment scheme of ATR inhibitor performed in *hUHRF1* and Control larvae. To determine the efficacy, H_2O_2 was used as DNA damage inducer. **B.** Western blot of Control and H_2O_2 treated larvae in presence and absence of the Atr inhibitor VE-821.

Appendix Figure S3

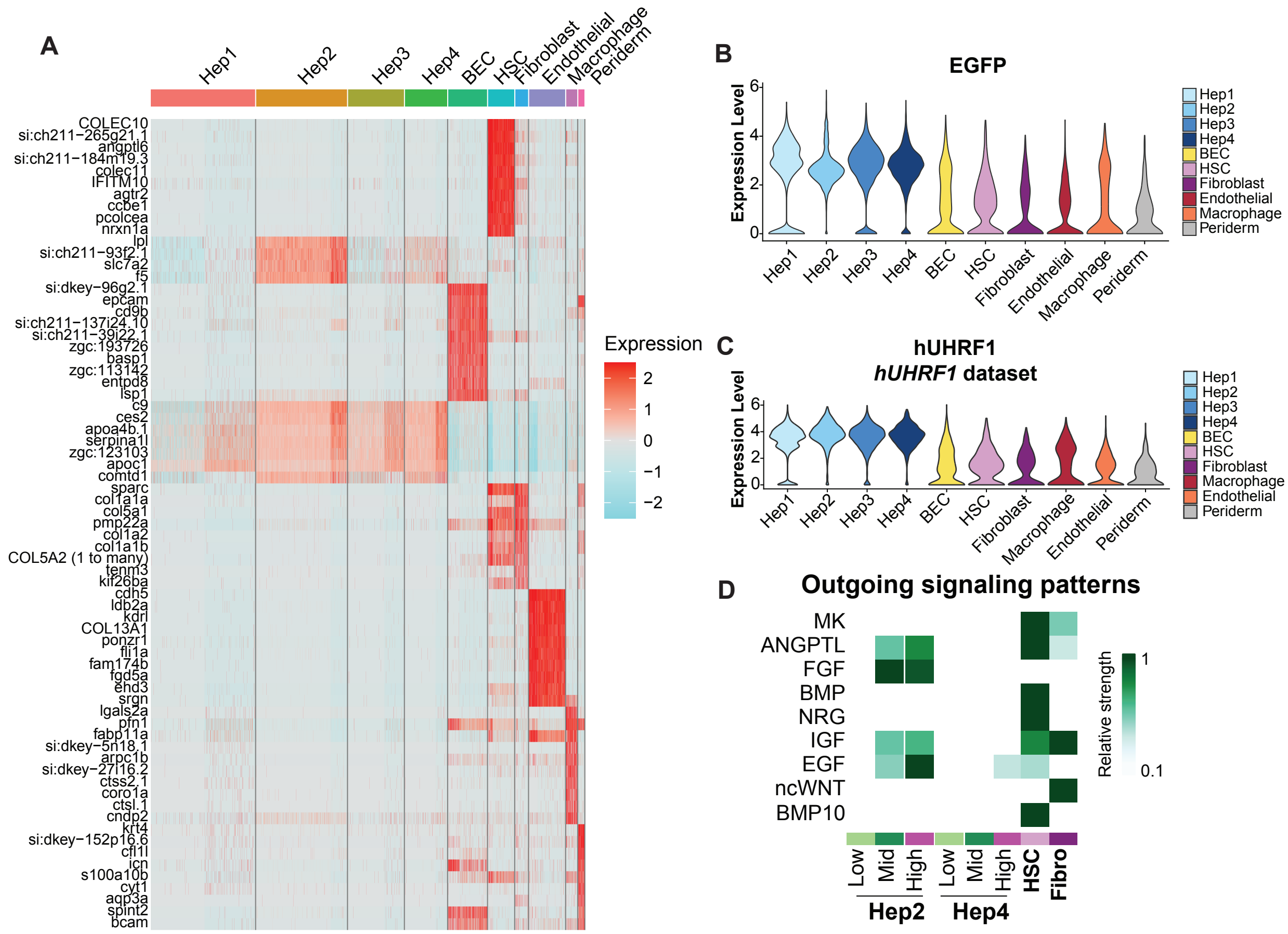

Appendix Figure S3. Single-cell RNAseq analysis. **A.** Heatmap of key identity markers in different cell populations. **B.** Violin plot of EGFP levels across populations in scRNAseq. **C.** Violin plot of hUHRF1 levels across populations in scRNAseq. **D.** Heatmap of outgoing signaling pathways in Hep population divided in high, mid, and low, mesenchymal cells and macrophages in *hUHRF1* samples.

Appendix Figure S4

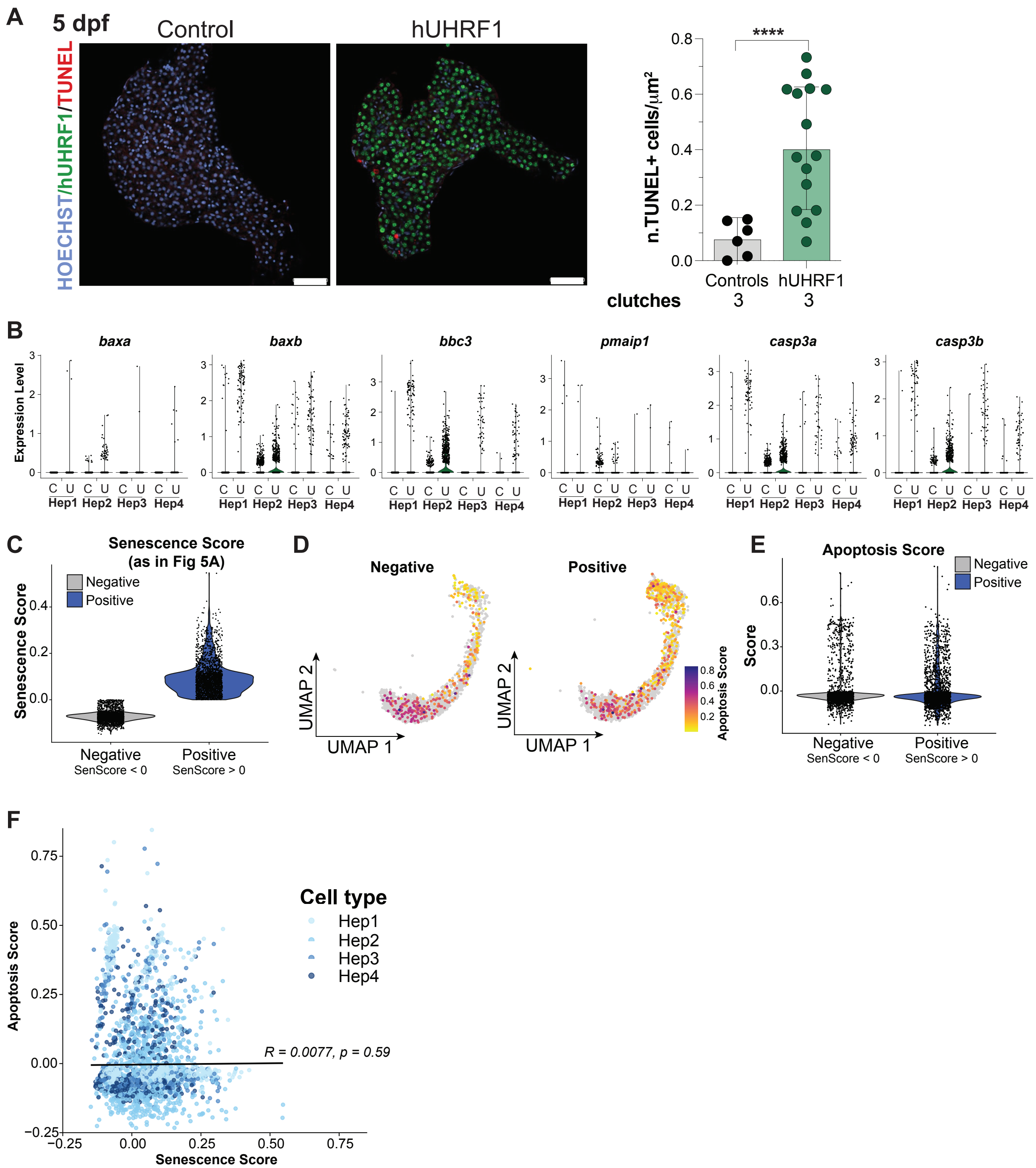

Appendix Figure S4. Apoptosis analysis of *hUHRF1* hepatocytes compared Control. **A.** TUNEL analysis of *hUHRF1* and control livers at 5 dpf. Dots indicate livers in 3 biological replicates (clutches). Data are presented as mean ± SD. **** < p-value 0.0001 by unpaired t-test. Exact p-value for the presented data is p-value = 3.577×10^{-5}. Scale bar: 50 μm. **B.** Violin plot of

genes involved in apoptosis across hepatocyte populations in *hUHRF1* and Controls in scRNseq. **C.** Violin plot of hUHRF1 hepatocytes categorized based on expression of Senescence Score as plotted in Figure 5A. Cells are scored as Positive if Senescence Score > 0 or negative if <0. Cells were randomly downsampled to achieve an equivalent number in each category. **D.** Feature plot of apoptosis score, calculated based on the genes plotted in B, across all hepatocytes segregated into positive and senescence negative cells based on Senescence Score as in C**. E.** Violin plot of the same score plotted in D. **F.** Correlation plot of Apoptosis Score and Senescence Score in each population hepatocytes in *hUHRF1* sample in scRNAseq. Each point represents one cell. R indicates Pearson's correlation coefficient. The association between senescence and apoptosis scores was assessed using a two-sided Pearson correlation test.

Appendix Figure S5

A

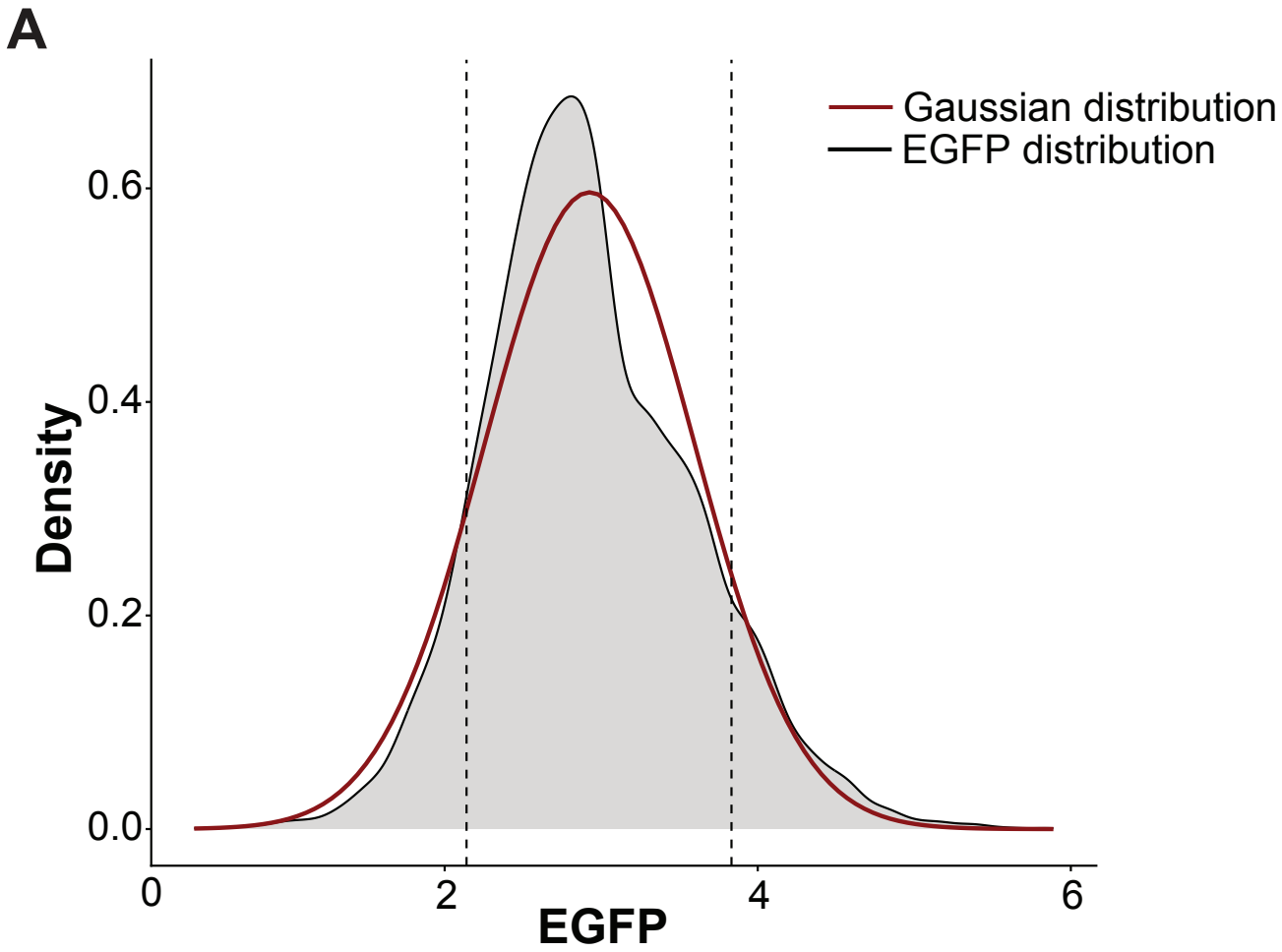

Appendix Figure S5. Gaussian distribution of EGFP. Gaussian distribution of EGFP in Control and hUHRF1 in scRNAseq. Dashed lines represent the lowest 10% quantile and the highest 10% quantile used to separate cells in High, Mid and Low.

Appendix Figure S6

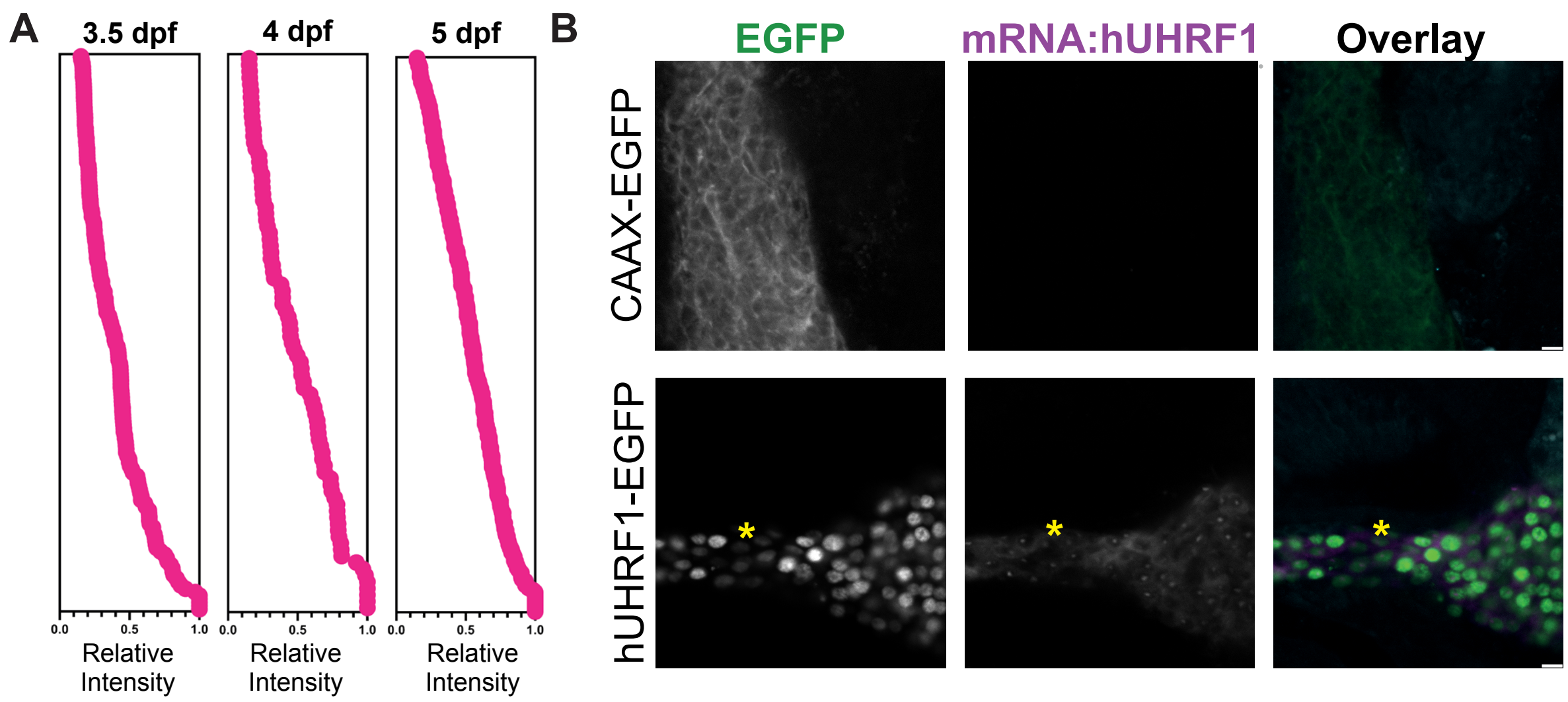

Appendix Figure S6. *hUHRF1* expression is heterogenous in hepatocytes.

A. Quantification of nls-mCherry signal in controls. **B.** In situ hybridization of hUHRF1 shows that cells with low/no GFP also have low/no hUHRF1 mRNA. Yellow asterisk indicates a liver area in which hUHRF1 protein levels and hUHRF1 mRNA levels are low compared to surrounding liver area. Scale bar: 10 μm.
