# Supplementary material for: UHRF1 overexpression generates distinct senescent states with different Tp53 dependencies
Source: EMBO Rep. 2026 Sep 28;27(21):6425–46. doi: 10.1038/s44319-026-00941-y (PMC13743750; doi:10.1038/s44319-026-00941-y)
Supplement: Supplementary file 3 [file 44319_2026_941_MOESM4_ESM.pdf]

UHRF1 Overexpression Generates Distinct Senescent States with Different Tp53 Dependencies

Elena Magnani, Charlene Chen, Filippo Macchi, Tijana Randic, Bhavani Madakashira, Ian McBain, and Kirsten Sadler

Corresponding author(s): Kirsten Sadler (kirsten.edepli@nyu.edu)

Review Timeline:

Transfer Date:	24th Jan 26
Editorial Decision:	30th Jan 26
Revision Received:	28th Apr 26
Editorial Decision:	29th May 26
Appeal Received:	20th Jul 26
Editorial Decision:	24th Aug 26
Revision Received:	29th Aug 26
Accepted:	4th Sep 26

Editor: Martina Rembold

Transaction Report: A revised version of this manuscript was transferred to EMBO reports following peer review at the EMBO Journal.

(Note: With the exception of the correction of typographical or spelling errors that could be a source of ambiguity, letters and reports are not edited. Depending on transfer agreements, referee reports obtained elsewhere may or may not be included in this compilation. Referee reports are anonymous unless the Referee chooses to sign their reports.)

Referee #1:

In the manuscript entitled "UHRF1 overexpression generates distinct senescent states with different tp53 dependencies" by Magnani and colleagues, the authors investigate the fate of hUHRF1 overexpression in zebrafish hepatocytes. The authors propose the generation of distinct senescent states with different Tp53 dependencies and investigate their response to senolytic treatment. However, they use descriptive data (imaging, bulk RNA-seq, scRNA-seq) and interpret them as evidence for mechanistically distinct senescent states with different p53, ATM/ATR, and senolytic sensitivity. The study relies heavily on indirect readouts, contains several mechanistic overinterpretations and introduces exciting concepts ("deep senescence", "pleiotropic senescent phenotypes") while simpler explanations are not ruled out.

While the goal of the authors is worthy and of general interest, the current version of the manuscript requires more mechanistic based experiments to sustain their interpretations. Therefore, I am not in favor of publication of the current study.

Major criticisms:
A central weakness of the study is the systematic use of RNA-seq (bulk and single-cell) to infer activation levels of pathways that are regulated at the protein and post-translational level.
1- The authors often mention increased tp53 transcript and expression of "p53 target genes" with "Tp53 activation" and use bulk RNA-seq to support this conclusion (e.g. Figure 1E-F, Figure 3C-E). p53 is regulated by protein stability and post-translational modifications and transcript levels are a very indirect readout. Without direct measurements of p53 protein abundance and phosphorylation, these claims are not sustained.
2- Similarly, the manuscript used transcriptomic signatures (SASP genes, cell cycle genes, tp53 targets) as sufficient to define senescent states in different hepatocyte clusters. Senescence is notoriously difficult to assess, and a visual SA-β-gal staining is required to support this assertion. However, the authors use off-the-shelf kits to measure SA-β-gal activity without localizing it to specific cells or tissues. False positives are common and attributable to unrelated cells. Without a co-localised SA-β-gal assessment (histochemical staining combined with hepatocyte EGFP), the strong functional conclusions ("deep senescent state", "pleiotropic senescent phenotypes", "escapable senescence") are therefore unreliable.
3- The authors argue that "genes involved in multiple DDR pathways, in particular homologous recombination and non-homologous end joining were induced" and interpret this as evidence that DDR pathways are activated and functionally engaged. DNA repair is

controlled by protein levels, complex formation, enzymatic activity and recruitment to damage sites. High mRNA levels do not show DNA repair activity or pathway choice.

4- The text describes γH2AX foci as a "marker of double strand breaks (DSBs)" and infers that UHRF1 overexpression "causes DSBs followed by formation of single stranded DNA" based on γH2AX and p-RPA staining at 80-96 hpf. This is an oversimplification. H2AX phosphorylation can be mediated by ATM, ATR or DNA-PK in response to various types of DNA damage and replication stress and is not specific to DSBs. γH2AX is a marker of DNA damage and that its phosphorylation is also associated with ATR-dependent signaling in response to ssDNA- and replication lesions. To draw the conclusion that DSBs precede ssDNA formation requires a co-localisation with S-phase marker (EdU, PCNA) to determine if, in contrast to the authors supposition, ssDNA originating from replication stress can precede the formation of DSBs.

5- The authors conclude that the ATM-p53 is required for hUHRF1-induced senescence, while ATR is dispensable based on SA-β-gal scoring in atm-/-, tp53-/-, VE-821-treated and combined atm-/- + VE-821 embryos. Given the negative result on ATR, the authors must provide VE-821 controls for ATR inactivation. The authors then extrapolate from early time points to a general conclusion that ATR "does not compensate" for ATM loss, but they do not examine later stages or functional outcomes (e.g., tumor incidence) in atm-/- + VE-821 embryos.

6- The authors use the term "deep senescence" to describe hepatocytes with high hUHRF1-EGFP expression, elevated Cdkn1a/Tp53 and SASP genes, and no detectable EdU incorporation even upon tp53 loss. However, "Deep senescence" is currently not defined by transcript abundance but by irreversible cell cycle exit and persistent senescence phenotypes. The same transcription profile could also reflect cells with DNA damage or entering apoptosis rather than a qualitatively distinct senescent state. To use this term, the authors should test the irreversibility of senescence phenotypes (e.g. using microscopy tracking experiments and/or induced regeneration). As currently framed, the author's "deep senescence" is a description of high expression of specific genes and not a biological state.

7- The manuscript does not distinguish "different senescent states" from hepatocyte subtypes with different sensitivity to hUHRF1/damage. In fact, the sensitivity to the oncogene may come from its over-expression in specific cells rather than the development of a new cell state. EdU positive cells at 10dpf are assumed to originate from "escaped" senescent cells, but they could simply be non-senescent survivors. The idea that these populations are direct tumour precursors is never tested. The authors should provide

evidence for origin of these populations in cancer cells (e.g. lineage tracing or tumour scRNA-seq).

8- The authors suggest that Navitoclax selectively removes specific senescent hepatocytes while allows for expansion of others promoting tumorigenesis. The evidence is limited to reduced SA-β-gal and modestly increased EdU incorporation. However, there is no direct demonstration which hepatocytes are affected apart from inference using expression of senolytic target genes in scRNA-seq. So, the suggestion that senolytics may promote tumor growth in this model is rather speculative.

Minor Criticism:

1. The authors mention "apoptosis pathways" from RNA data but there is no direct measurement of apoptosis (e.g. TUNEL, cleaved caspase). It is unclear whether cells with "deep" transcriptional signatures are truly senescent, pre-apoptotic, or dying.
2. Binning strategies for "high/mid/low" EGFP and senescence signatures in scRNA-seq are arbitrary with limited statistical testing of differences between classes.

Referee #2:

Senescence induced by UHRF1 overexpression in zebrafish has been previously established. Magnani et al. further characterised senescence heterogeneity in this model using methylome and bulk/scRNA-seq analyses. Interestingly, they identified four hepatocyte clusters, with "Hep 2" showing a more robust senescence score. Their data indicate that ectopic UHRF1 levels contribute to senescence heterogeneity. They also show that Hep 2 contains a higher proportion of high-UHRF1 cells, and that p53 depletion promotes proliferation in low-UHRF1 cells but not in high-UHRF1 cells. Given that UHRF1 is commonly upregulated in cancer, including liver cancer, and that senescence heterogeneity is an important emerging topic, the experiments are well conducted and the findings are highly relevant. However, there are several points the authors should consider to strengthen the manuscript.

Fig. 1L. The data seem to suggest that these genes are downregulated in 120 hpf controls rather than upregulated in 120 hpf UHRF1-overexpressing samples. Can the authors clarify this? Are these genes normally silenced at this developmental stage but remain expressed when UHRF1 is overexpressed? Or could this be due to a normalisation issue?

I also wonder whether the authors can provide correlations between UHRF1 levels and

DNA methylation, gene expression, and TE activation. Can their scRNA-seq dataset detect any transposable element or LTR-derived transcripts?

Fig. 4-5. The scRNA-seq data at 120 hpf reveal four hepatocyte clusters in control fish. I wonder whether these clusters correspond to known features of hepatocyte heterogeneity, such as metabolic zonation, cell-cycle states, or maturation gradients. It would be helpful if the authors could project these features onto the UMAP (e.g., Fig. 4B, Fig. 5C). In addition, a pseudotime analysis could provide insight into potential maturation trajectories among these hepatocyte subsets.

Regarding the cell-cycle analysis in Fig. 5B, the selected genes appear somewhat limited. Including canonical E2F targets/S-phase markers (e.g., CCNA, PCNA, MCM family genes) or applying a "cell-cycle signature" may strengthen the assessment.

For the CellChat analysis (e.g. Fig. 5F), it is unclear whether each cluster contains a mixture of control and UHRF1 cells, or focused only on UHRF1 fish. It may be more informative to visualise signalling networks for control and UHRF1 conditions separately to highlight condition-specific changes.

Finally, the model in Fig. 6D is intriguing. If this zebrafish model is indeed tumorigenic, it would be informative to know whether, in extended follow-up, the cancer-initiating population corresponds to the low-UHRF1, p53-deficient hepatocytes.

Dear Prof. Sadler

Thank you for the transfer of your manuscript to EMBO reports. As discussed, I would be happy to consider your manuscript for publication at EMBO Reports if the main reviewer comments can be addressed satisfactorily, as outlined in your revision plan. It will be important to complement your RNA-based analysis with functional and protein-based assays, to expand the analysis of the proposed senescent states and to address all concerns regarding the Navitoclax treatment.

I would thus like to invite you to revise your manuscript for EMBO reports. Please address all referee concerns in a complete point-by-point response. Acceptance of the manuscript will depend on a positive outcome of a second round of review. I will walk the referees through our transfer process. It is EMBO reports policy to allow a single round of major revision only and acceptance or rejection of the manuscript will therefore depend on the completeness of your responses included in the next, final version of the manuscript.

We realize that it is difficult to revise to a specific deadline. In the interest of protecting the conceptual advance provided by the work, we recommend a revision within 3 months (2nd May 2026). Please discuss the revision progress ahead of this time with me if you require more time to complete the revisions.

You can either publish the study as a short report or as a full article. For short reports, the revised manuscript should not exceed 27,000 characters (including spaces but excluding materials & methods and references) and 5 main plus 5 expanded view figures. The results and discussion sections must further be combined, which will help to shorten the manuscript text by eliminating some redundancy that is inevitable when discussing the same experiments twice. For a normal article there are no length limitations, but it should have more than 5 main figures and the results and discussion sections must be separate. In both cases, the entire materials and methods must be included in the main manuscript file.

IMPORTANT NOTE: we perform an initial quality control of all revised manuscripts before re-review. Your manuscript will FAIL this control and the handling will be DELAYED if the following APPLIES:
1) A data availability section providing access to data deposited in public databases is missing. If you have not deposited any data, please add a sentence to the data availability section that explains that.
2) Your manuscript contains statistics and error bars based on n=2. Please use scatter blots in these cases. No statistics should be calculated if n=2.

When submitting your revised manuscript, please review the submission guidelines (https://link.springer.com/journal/44319/submission-guidelines#cms-Revised-submissions) and carefully review the instructions that follow below. Failure to include requested items will delay the evaluation of your revision.

1) a .docx formatted version of the manuscript text (including legends for main figures, EV figures and tables). Please make sure that the changes are highlighted to be clearly visible.

2) individual production quality figure files as .eps, .tif, .jpg (one file per figure). See hhttps://media.springernature.com/original/springer-cms/rest/v1/content/27825798/data/v1 for more info on how to prepare your figures.

3) We replaced Supplementary Information with Expanded View (EV) Figures and Tables that are collapsible/expandable online. A maximum of 5 EV Figures can be typeset. EV Figures should be cited as 'Figure EV1, Figure EV2" etc... in the text and their respective legends should be included in the main text after the legends of regular figures.

- For the figures that you do NOT wish to display as Expanded View figures, they should be bundled together with their legends in a single PDF file called *Appendix*, which should start with a short Table of Content. Appendix figures should be referred to in the main text as: "Appendix Figure S1, Appendix Figure S2" etc. See 'Expanded View' section of the submission guidelines.

- Additional Tables/Datasets should be labeled and referred to as Table EV1, Dataset EV1, etc. Legends have to be provided in a separate tab in case of .xls files. Alternatively, the legend can be supplied as a separate text file (README) and zipped together with the Table/Dataset file.

4) a .docx formatted letter INCLUDING the reviewers' reports and your detailed point-by-point responses to their comments. As part of the EMBO Press transparent editorial process, the point-by-point response is part of the Review Process File (RPF), which will be published alongside your paper.

5) a complete author checklist, which you can download from our author guidelines. Please insert information in the checklist that is also reflected in the manuscript. The completed author checklist will also be part of the RPF.

6) Please note that all corresponding authors are required to supply an ORCID ID for their name upon submission of a revised

manuscript (<https://orcid.org/>), which can be linked to your profile in the manuscript submission system.

7) Before submitting your revision, primary datasets produced in this study need to be deposited in an appropriate public database. Please remember to provide a reviewer password if the datasets are not yet public. The accession numbers and database should be listed in a formal "Data Availability" section placed after Methods. Please note that the Data Availability Section is restricted to new primary data that are part of this study. * Note - All links should resolve to a page where the data can be accessed. *

If your study has not produced novel datasets, please mention this fact in the Data Availability Section.

8) At EMBO Press we ask authors to provide source data for the main manuscript figures. You will receive a separate email with instructions for providing source data with your revised manuscript, including how to upload and organize the files.

9) Our journal also encourages inclusion of *data citations in the reference list* to directly cite datasets that were re-used and obtained from public databases. Data citations in the article text are distinct from normal bibliographical citations and should directly link to the database records from which the data can be accessed. In the main text, data citations are formatted as follows: "Data ref: Smith et al, 2001" or "Data ref: NCBI Sequence Read Archive PRJNA342805, 2017". In the Reference list, data citations must be labeled with "[DATASET]". A data reference must provide the database name, accession number/identifiers and a resolvable link to the landing page from which the data can be accessed at the end of the reference.

10) Regarding data quantification, the following points must be specified in each figure legend:

- the name of the statistical test used to generate error bars and P values,

- the number (n) of independent experiments (please specify technical or biological replicates) underlying each data point,

- the nature of the bars and error bars (s.d., s.e.m.),

- If the data are obtained from n Program fragment delivered error ``Can't locate object method "less" via package "than" (perhaps you forgot to load "than"?) at //ejpvfs23/sites23b/embor_www/letters/embor_decision_revise_and_review.txt line 54.' 2, use scatter blots showing the individual data points.

Discussion of statistical methodology can be reported in the materials and methods section, but figure legends should contain a basic description of n, P and the test applied.

- Please also include scale bars in all microscopy images.

11) The journal requires a statement specifying whether or not authors have competing interests (defined as all potential or actual interests that could be perceived to influence the presentation or interpretation of an article). In case of competing interests, this must be specified in your disclosure statement.

12) All Materials and Methods need to be described in the main text using our 'Structured Methods' format, which is required for all research articles. According to this format, the Methods section includes a Reagents and Tools Table (listing key reagents, experimental models, software and relevant equipment and including their sources and relevant identifiers) followed by a Methods and Protocols section describing the methods using a step-by-step protocol format. The aim is to facilitate adoption of the methodologies across labs. More information on how to adhere to this format as well as a downloadable template (.docx) for the Reagents and Tools Table can be found in our author guidelines.

We would also welcome the submission of cover suggestions, or motifs to be used by our Graphics Illustrator in designing a cover.

As part of the EMBO publication's Transparent Editorial Process, EMBO reports publishes online a Review Process File (RPF) to accompany accepted manuscripts. This File will be published in conjunction with your paper and will include the referee reports, your point-by-point response and all pertinent correspondence relating to the manuscript.

You are able to opt out of this by letting the editorial office know (emboreports@embo.org). If you do opt out, the Review Process File link will point to the following statement: "No Review Process File is available with this article, as the authors have chosen not to make the review process public in this case."

I look forward to seeing a revised form of your manuscript when it is ready.

Kind regards,
Martina

Martina Rembold, PhD
Senior Editor
EMBO reports

We appreciate the positive comments and constructive criticism on the manuscript. We have responded to each query through additional experiments and data analysis and revisions to the text. Each reviewer comment is listed in full below, with our response in blue text.

Referee #1:

In the manuscript entitled "UHRF1 overexpression generates distinct senescent states with different tp53 dependencies" by Magnani and colleagues, the authors investigate the fate of hUHRF1 overexpression in zebrafish hepatocytes. The authors propose the generation of distinct senescent states with different Tp53 dependencies and investigate their response to senolytic treatment. However, they use descriptive data (imaging, bulk RNA-seq, scRNA-seq) and interpret them as evidence for mechanistically distinct senescent states with different p53, ATM/ATR, and senolytic sensitivity. The study relies heavily on indirect readouts, contains several mechanistic overinterpretations and introduces exciting concepts ("deep senescence", "pleiotropic senescent phenotypes") while simpler explanations are not ruled out.

While the goal of the authors is worthy and of general interest, the current version of the manuscript requires more mechanistic based experiments to sustain their interpretations. Therefore, I am not in favor of publication of the current study.

Major criticisms:

A central weakness of the study is the systematic use of RNA-seq (bulk and single-cell) to infer activation levels of pathways that are regulated at the protein and post-translational level.

We appreciate the suggestions from this reviewer that additional experimental data are of interest to improve the story. We agree and have addressed this directly through additional experiments and analysis. At the same time, we emphasize that scRNA-seq is a state-of-the-art approach to identify distinct cell populations in a heterogeneous pre-cancer model that has been used extensively in the literature. We coupled this approach with mechanistic genetic and pharmacological interventions to demonstrate that different cell populations induced by UHRF1 overexpression have distinct outcomes. While protein level validation of the RNA expression data can strengthen the findings, it does not alter the overall conclusions or impact of the study.

1. The authors often mention increased tp53 transcript and expression of "p53 target genes" with "Tp53 activation" and use bulk RNA-seq to support this conclusion (e.g. Figure 1E-F, Figure 3C-E). p53 is regulated by protein stability and post-translational modifications and transcript levels are a very indirect readout. Without direct measurements of p53 protein abundance and phosphorylation, these claims are not sustained.

This is a valuable point. To further confirm the RNA expression analysis and functional genetic data showing that UHRF1 overexpression activates Tp53, we used Western blotting of dissected livers as the standard method in zebrafish to assess Tp53 levels, since there are no optimized

antibodies in this model for phosphorylated Tp53, and assessing Tp53 using immunofluorescence is challenging for the entire zebrafish research community. **NEW Figure 3A** shows Tp53 protein is significantly elevated in the liver of *Tg(fabp10a:hUHRF1-GFP)* larvae at 5 dpf, further supporting the conclusion that UHRF1 overexpression activates Tp53 and downstream targets.

2. Similarly, the manuscript used transcriptomic signatures (SASP genes, cell cycle genes, tp53 targets) as sufficient to define senescent states in different hepatocyte clusters. Senescence is notoriously difficult to assess, and a visual SA-β-gal staining is required to support this assertion. However, the authors use *off-the-shelf kits* to measure SA-β-gal activity without localizing it to specific cells or tissues. False positives are common and attributable to unrelated cells. Without a co-localized SA-β-gal assessment (histochemical staining combined with hepatocyte EGFP), the strong functional conclusions ("deep senescent state", "pleiotropic senescent phenotypes", "escapable senescence") are therefore unreliable.

We agree with the reviewer on several of the points here and have addressed them directly. First, since SA-β-gal staining on tissue sections generates a diffuse stain across the tissue, making cellular resolution impossible, we optimized a SA-β-gal staining protocol for whole mount zebrafish larvae, as previously published (Magnani *et al*, 2024b; Mudbhary *et al*, 2014) and shown in Reviewer Figure 1. Using this protocol, we generated **NEW Figure 1B** showing images of whole mount SA-β-gal stained larvae and EGFP in the same *Tg(fabp10a:hUHRF1-EGFP)* larvae to show that staining is localized to the liver, overlaps with EGFP, and we have quantified the percent of larvae with SA-β-gal positive livers over the time course of senescence induction in this model.

Figure for referee with unpublished data and its description has been removed upon request by the authors.

3. The authors argue that "genes involved in multiple DDR pathways, in particular homologous recombination and non-homologous end joining were induced" and interpret this as evidence that DDR pathways are activated and functionally engaged. DNA repair is controlled by protein levels, complex formation, enzymatic activity and recruitment to damage sites. High mRNA levels do not show DNA repair activity or pathway choice.

We thank the reviewer for highlighting that RNA expression of DNA damage response genes does not demonstrate active DNA repair and have modified the text to reflect this caveat. We point to the functional genetic data using *atm* mutants (verified in (Ajouaou *et al*, 2023)) which abrogates SA-β-gal in *Tg(fabp10a:hUHRF1-EGFP)* 5 dpf larvae (Figure 3B). This provides an important functional consequence of our finding that Tp53 protein levels and target genes are induced, supporting the conclusion that the *atm-tp53* axis is required for senescence in this model. We have added to the discussion that the dependency on *atm* could reflect a distinct cell cycle stage in which DNA damage occurs, as shown by Hsu and colleagues (Hsu *et al*, 2019).

4. The text describes γH2AX foci as a "marker of double strand breaks (DSBs)" and infers that UHRF1 overexpression "causes DSBs followed by formation of single stranded DNA" based on γH2AX and p-RPA staining at 80-96 hpf. This is an oversimplification. H2AX phosphorylation can be mediated by ATM, ATR or DNA-PK in response to various types of DNA damage and replication stress and is not specific to DSBs. γH2AX is a marker of DNA damage and that its phosphorylation is also associated with ATR-dependent signaling in response to ssDNA- and replication lesions. To draw the conclusion that DSBs precede ssDNA formation requires a co-localization with S-phase marker (EdU, PCNA) to determine if, in contrast to the authors supposition, ssDNA originating from replication stress can precede the formation of DSBs.

We appreciate the reviewer highlighting this nuance of γH2AX foci. Since there are so few cells in hUHRF1 livers that are positive for EdU, and many more cells with γH2AX foci, any overlap of these markers would represent a fraction of the γH2AX positive cells and quantification would rely on assessment in a very small number of all hepatocytes making interpretation difficult. We have further clarified that while H2AX phosphorylation is widely used as a marker of DSB, it can reflect different types of DNA damage and have modified the text to include this interpretation.

5. The authors conclude that the ATM-p53 is required for hUHRF1-induced senescence, while ATR is dispensable based on SA-β-gal scoring in atm-/-, tp53-/-, VE-821-treated and combined atm-/- + VE-821 embryos. Given the negative result on ATR, the authors must provide VE-821 controls for ATR inactivation.

We appreciate the reviewer's comment about the role of Atr in this model. We used a previously optimized protocol to inhibit Atr using VE-821 in zebrafish larvae (Madakashira *et al*, 2024) and demonstrate its efficacy by showing that it effectively blocks activation of Chk1 as assessed by phospho-Chk1 Western Blot (Appendix Figure S2). While the most logical conclusion from the finding that VE-821 does not affect SA-β-gal in *Tg(fabp10a:hUHRF1-EGFP)* is that Atr is not required for this phenotype, it is possible that other aspects of the phenotype caused by hUHRF1 are affected by Atr. We have clarified this in the text and will investigate this important question in future experiments.

6. The authors then extrapolate from early time points to a general conclusion that ATR "does not compensate" for ATM loss, but they do not examine later stages or functional outcomes (e.g., tumor incidence) in atm-/- + VE-821 embryos.

This is a very interesting point, and we agree with the reviewer that we do not know if, at later stages, there is a compensatory relationship between Atm and Atr. We have clarified this in the text. We have been studying the relationship between senescence and UHRF1-induced tumors extensively but not included that data in this paper as it goes beyond the scope of the current study. We are working on a subsequent study to examine how intervention in early stages of tumorigenesis impacts cancer related outcomes and will explore this exciting new area in future studies.

7. The authors use the term "deep senescence" to describe hepatocytes with high hUHRF1-EGFP expression, elevated Cdkn1a/Tp53 and SASP genes, and no detectable EdU incorporation even

upon tp53 loss. However, "Deep senescence" is currently not defined by transcript abundance but by irreversible cell cycle exit and persistent senescence phenotypes. The same transcription profile could also reflect cells with DNA damage or entering apoptosis rather than a qualitatively distinct senescent state. To use this term, the authors should test the irreversibility of senescence phenotypes (e.g. using microscopy tracking experiments and/or induced regeneration). As currently framed, the author's "deep senescence" is a description of high expression of specific genes and not a biological state.

We appreciate this perspective and agree that the term Deep Senescence is not well defined in the field. We utilize standard assays as recommended by the recent important reference outlining the Minimal Information for Senescence Experiments *in vivo* (Ogrodnik *et al*, 2024) to define this phenotype in zebrafish. We propose that the model of UHRF1-induced senescence enables functional assessment of different senescence phenotypes.

To address this interesting idea proposed by the reviewer that deep senescent cells should be resistant to proliferative signals, we used a recently published system in which the nitroreductase (NTR) gene is induced mosaically by a Cre-mediated induction to allow partial ablation when treated with Metronidazole (MTZ; (Eski *et al*, 2025)). This enables investigation of the response of the remaining cells, and potentially asking whether some hepatocytes in hUHRF1 larvae were refractory to this regeneration induced signal. We successfully generated mosaic NTR-mcherry expression and at 5 and 6 dpf; however the treatment with MTZ did not eliminate the NTR-mCherry expressing cells (See **Reviewer Figure 2**), despite the ability of MTZ to eliminate NTR expressing hepatocytes at later time points or using a different model where NTR is expressed under a constitutive hepatocyte promoter (not shown). We discussed this finding with the lab that generated this tool and they shared similar findings which are attributed to lack of sufficient NTR accumulation shortly after Cre-mediated induction of the transgene. Therefore, the proposed experiment is not technically feasible given the current tools available. We modified use of the term “deep senescence” and we defined senescence phenotypes based on molecular markers instead of a functional readout.

Figure for referee with unpublished data and its description has been removed upon request by the authors.

7. The manuscript does not distinguish "different senescent states" from hepatocyte subtypes with different sensitivity to hUHRF1/damage. In fact, the sensitivity to the oncogene may come from its over-expression in specific cells rather than the development of a new cell state. EdU positive

cells at 10 dpf are assumed to originate from "escaped" senescent cells, but they could simply be non-senescent survivors. The idea that these populations are direct tumor precursors is never tested. The authors should provide evidence for origin of these populations in cancer cells (e.g. lineage tracing or tumor scRNA-seq).

We agree with the reviewer that discerning between these nuances is important to understand the different responses to UHRF1 overexpression. This is the focus of an extensive study by our group, partially addressed in a preprint which is now being prepared for submission (Magnani *et al*, 2024a). Here, our focus is on the early response to hUHRF1 overexpression as this is important to address since this potentially represents the initial cause of some types of cancer and has not been investigated previously. We proposed that fully addressing this query is beyond the scope of the current study.

8. The authors suggest that Navitoclax selectively removes specific senescent hepatocytes while allows for expansion of others promoting tumorigenesis. The evidence is limited to reduced SA-β-gal and modestly increased EdU incorporation. However, there is no direct demonstration which hepatocytes are affected apart from inference using expression of senolytic target genes in scRNA-seq. So, the suggestion that senolytic may promote tumor growth in this model is rather speculative.

This is a good point that we have addressed with new experimental data and new analyses. First, we optimized the timing and length of Navitoclax treatment to maximize the removal of senescent cells (**NEW Figure 6C**). We then used this optimized treatment protocol to ask whether Navitoclax eliminated distinct cell populations based on hUHRF1-EGFP levels. To do this, we compared EGFP levels to a hepatocyte marker *(Tg(fabp10a:nls-mCherry*) in response to Navitoclax treatment compared to controls. As shown in **NEW Figure 6E** as well as in reanalyzed data in **NEW Figure 5H,** the level of hUHRF1-EGFP is highly heterogenous in untreated larvae, shown by its high variation compared to nls-mCherry levels. Navitoclax treatment reduces UHRF1-EGFP heterogeneity significantly, shown by the reduction in EGFP levels in most cells to be comparable to nls-mCherry. This indicates that Navitoclax either eliminates cells with high hUHRF1-EGFP expression or, less likely, regulates the levels of hUHRF1-EGFP. We conclude the former explanation is best supported by the evidence.

Minor Criticism:

1. The authors mention "apoptosis pathways" from RNA data but there is no direct measurement of apoptosis (e.g. TUNEL, cleaved caspase). It is unclear whether cells with "deep" transcriptional signatures are truly senescent, pre-apoptotic, or dying.

Figure for referee with unpublished data and its description has been removed upon request by the authors.

Good point. We have addressed this with new data and analysis. In **NEW Appendix Figure S4,** we show TUNEL staining and induction of apoptosis regulators in some cells in this model. To determine whether the cells with a "deep" transcriptional signature were indeed apoptotic cells we separated all the cells in *hUHRF1* scRNA-seq dataset with Senescence Score (Figure 5A) higher than 0 and randomly selected the same amount of *hUHRF1* cells with Senescence Score lower than 0 as shown in **Reviewer Figure 3A**. We then compared the expression levels of apoptosis effectors shown in **NEW Appendix Figure S4** and found that they are not enriched in cells with high Senescence Score (**Reviewer Figure 3B**). This suggests that dying cells are not enriched in populations that we identified as senescent.

2. Binning strategies for "high/mid/low" EGFP and senescence signatures in scRNA-seq are arbitrary with limited statistical testing of differences between classes.

We thank the reviewer for pointing this out. We used quartiles to define the range of hUHRF1-EGFP and we have now included the Gaussian distribution to differentiate high/mid/low in **NEW Appendix Figure S5**.

Referee #2:

Senescence induced by UHRF1 overexpression in zebrafish has been previously established. Magnani et al. further characterized senescence heterogeneity in this model using methylome and bulk/scRNA-seq analyses. Interestingly, they identified four hepatocyte clusters, with "Hep 2" showing a more robust senescence score. Their data indicate that ectopic UHRF1 levels contribute to senescence heterogeneity. They also show that Hep 2 contains a higher proportion of high-UHRF1 cells, and that p53 depletion promotes proliferation in low-UHRF1 cells but not in high-UHRF1 cells. Given that UHRF1 is commonly upregulated in cancer, including liver cancer, and that senescence heterogeneity is an important emerging topic, the experiments are well conducted, and the findings are highly relevant. However, there are several points the authors should consider strengthening the manuscript.

1. Fig. 1L. The data seem to suggest that these genes are downregulated in 120 hpf controls rather than upregulated in 120 hpf UHRF1-overexpressing samples. Can the authors clarify this? Are these genes normally silenced at this developmental stage but remain expressed when UHRF1 is overexpressed? Or could this be due to a normalization issue?

We thank the reviewer for highlighting this aspect of our study. As pointed out by the reviewer, normalization of the counts showed in Figure 1L was performed on 80 hpf (Controls and hUHRF1) and 120 hpf (Controls and hUHRF1) separately, which upon further analysis revealed a normalization bias. We therefore reanalyzed this data using a normalization approach including both genotypes and both time points. We further responded to this query by investigating all genes in the DNA damage response geneset. The analysis presented in **NEW Figure 1K** shows that 70% of the genes involved in DNA damage response that were upregulated at 80 hpf were also upregulated at 120 hpf. Therefore, we conclude that the DNA damage response is an early and sustained response to UHRF1 overexpression.

2. I also wonder whether the authors can provide correlations between UHRF1 levels and DNA methylation, gene expression, and TE activation. Can their scRNA-seq dataset detect any transposable element or LTR-derived transcripts?

We recognize this is an interesting point and we are very interested in studying the impacts of TEs on the tumor phenotype. We show that a subset of TEs is upregulated as an early response to UHRF1 levels using bulk RNAseq and have preliminary data on TE expression using scRNA-seq analysis. However, comparison with the bulk RNAseq data is complicated by a difference in sequencing depth and the implications of this are unclear so have chosen not to include this in the current version of the manuscript but will focus on this interesting question in the future.

3. Fig. 4-5. The scRNA-seq data at 120 hpf reveal four hepatocyte clusters in control fish. I wonder whether these clusters correspond to known features of hepatocyte heterogeneity, such as metabolic zonation, cell-cycle states, or maturation gradients. It would be helpful if the authors could project these features onto the UMAP (e.g., Fig. 4B, Fig. 5C). In addition, a pseudotime analysis could provide insight into potential maturation trajectories among these hepatocyte subsets.

Figure for referee with unpublished data and its description has been removed upon request by the authors.

We thank the reviewer for this insight and have performed additional analysis as suggested. The differences among the 4 hepatocyte clusters in controls reflect different stages of maturation. Since the architecture of the zebrafish liver is different from mammals and lacks the lobular structure with no evidence of zonation, zonation markers are not clustered in distinct populations in control zebrafish livers (see **Reviewer Figure 3A**), and the hepatocyte clusters do not differ significantly for cell cycle phases (see **review Figure 3B**).

To determine whether these clusters reflected stages of hepatocyte maturation, we used a list of specific hepatoblast markers defined in the single cell atlas of developing zebrafish (DanioCell; https://daniocell.nichd.nih.gov/). We found that Hep2 is the most enriched for hepatoblast markers, and was farthest on pseudotime from Hep1, suggesting a trajectory whereby Hep2 is the most immature and differentiates into the other hepatocyte clusters. These data are presented in **NEW Extended Figure EV2**.

4. Regarding the cell-cycle analysis in Fig. 5B, the selected genes appear somewhat limited. Including canonical E2F targets/S-phase markers (e.g., CCNA, PCNA, MCM family genes) or applying a "cell-cycle signature" may strengthen the assessment.

We thank the reviewer for this helpful comment. We used a set of canonical genes that are highly expressed in S-phase to assess the cell-cycle. Not all of them were found in the list of differentially expressed genes but we included the ones that were presented in **UPDATED Figure 5B**. Interestingly, Hep2 cells have high expression of cell cycle genes involved in DNA repair as *pcna* and nucleotide synthesis *rrm2* but low expression of genes such as the *mcms* that are involved in DNA replication genes.

5. For the CellChat analysis (e.g. Fig. 5F), it is unclear whether each cluster contains a mixture of control and UHRF1 cells or focused only on UHRF1 fish. It may be more informative to visualize signaling networks for control and UHRF1 conditions separately to highlight condition-specific changes.

We apologize for the confusion. The analysis presented in the paper is performed on *hUHRF1* alone. We clarified in the text and have responded to the reviewer's request by performing additional analysis of hepatocytes Controls (**NEW Extended Figure EV2**).

Finally, the model in Fig. 6D is intriguing. If this zebrafish model is indeed tumorigenic, it would be informative to know whether, in extended follow-up, the cancer-initiating population corresponds to the low-UHRF1, p53-deficient hepatocytes.

This is a very interesting point which we would also like to know the answer top. Hoewver, addressing it is beyond the scope of this study as we are undertaking a full analysis of the mechanism of tumorigenesis in this model and will present that data in an independent manuscript that is in preparation.

References

Ajouaou Y, Magnani E, Madakashira B, Jenkins E, Sadler KC (2023) atm Mutation and Oxidative Stress Enhance the Pre-Cancerous Effects of UHRF1 Overexpression in Zebrafish Livers. *Cancers* 15

Eski SE, Mi J, Pozo-Morales M, Hovhannisyan GG, Perazzolo C, Manco R, Ez-Zammoury I, Barbhaya D, Lefort A, Libert F *et al* (2025) Cholangiocytes contribute to hepatocyte regeneration after partial liver injury during growth spurt in zebrafish. *Nat Commun* 16: 5260

Hsu CH, Altschuler SJ, Wu LF (2019) Patterns of Early p21 Dynamics Determine Proliferation-Senescence Cell Fate after Chemotherapy. *Cell* 178: 361-373 e312

Madakashira BP, Magnani E, Ranjan S, Sadler KC (2024) DNA hypomethylation activates Cdk4/6 and Atr to induce DNA replication and cell cycle arrest to constrain liver outgrowth in zebrafish. *Nucleic Acids Res* 52: 3069-3087

Magnani E, Macchi F, Randic T, Chen C, Madakashira B, Ranjan S, Eski SE, Singh SP, Sadler KC (2024a) Epigenetic Disordering Drives Stemness, Senescence Escape and Tumor Heterogeneity. *bioRxiv*

Magnani E, Nair AR, McBain I, Delaney P, Chu J, Sadler KC (2024b) Methods to Study Liver Disease Using Zebrafish Larvae. *Methods Mol Biol* 2707: 43-69

Mudbhary R, Hoshida Y, Chernyavskaya Y, Jacob V, Villanueva A, Fiel MI, Chen X, Kojima K, Thung S, Bronson RT *et al* (2014) UHRF1 overexpression drives DNA hypomethylation and hepatocellular carcinoma. *Cancer Cell* 25: 196-209

Ogrodnik M, Carlos Acosta J, Adams PD, d'Adda di Fagagna F, Baker DJ, Bishop CL, Chandra T, Collado M, Gil J, Gorgoulis V *et al* (2024) Guidelines for minimal information on cellular senescence experimentation in vivo. *Cell* 187: 4150-4175

Editorial Decision **29th May 2026**

Dear Prof. Sadler

Thank you for the submission of your revised manuscript to EMBO reports. We have now received the comments from the referees that were asked to assess it.

I am sorry to say that the evaluation of your manuscript is not a positive one. As you will see, while the referees acknowledge that you have addressed several of their concerns in the revised version, referee 1 is not convinced that the data provided are sufficiently strong to support your key conclusions.

Given these substantial concerns, the fact that you already had a chance to significantly revise the study and that EMBO reports allows a single round of revision only, I am afraid that we cannot offer to publish the manuscript at this point. I am sorry that this decision emerges as the outcome of a lengthy review process but given the significant concerns that remain, I have no other option but to reject your manuscript.

I am sorry that I could not bring better news this time and hope that the referee comments will be helpful in your continued work in this area.

Kind regards,

Martina Rembold, PhD
Senior Editor
EMBO reports

Referee #1:

I thank the authors for the revised manuscript that has improved through the addition of new data and clarifications. Nevertheless, my main concern remains unresolved. The manuscript repeatedly converts correlative observations into mechanistic conclusions that are not fully supported by the experiments. This problem affects the interpretation of the scRNAseq data, the definition of senescent states and the conclusions drawn from the Navitoclax treatment. In the current form, the study does not yet support the stronger claims of mechanistically distinct senescent states or selective senolytic removal of a defined senescent population. I therefore recommend major revision.
Major points
1. The manuscript still equates transcriptional heterogeneity with functionally distinct senescent states. The scRNA-seq analysis identifies heterogeneous hepatocyte profiles, but these profiles do not establish distinct senescent populations in a functional sense. In particular, the interpretation of Hep2 and high-UHRF1 cells as biologically distinct senescent states remains uncertain. There is no direct cell-resolved evidence that these specific populations are SA-β-gal-positive, irreversibly arrested, resistant to proliferative cues, or otherwise functionally senescent. At present, the data support heterogeneity among stressed hepatocytes, not conclusive evidence for distinct senescent states.
2. The DDR section continues to present an inferred model as if it were experimentally resolved. γH2AX and p-RPA are compatible with DNA damage and/or replication stress, but they do not establish the specific sequence proposed by the authors. The manuscript still implies that UHRF1 first induces double-strand breaks and subsequently single-stranded DNA formation. This order is not demonstrated. The data support the presence of DNA damage response markers, but they do not define the initiating lesion, the pathway hierarchy, or the causal progression of the response..
3. The term "deep senescence" remains insufficiently supported. The manuscript does not demonstrate irreversible arrest, persistence of the state over time, stability after removal of stress, or failure to respond to regenerative stimuli. The unsuccessful regeneration experiment described in the response cannot be used as supporting evidence. At most, the data identify high-UHRF1, non-proliferative cells enriched for senescence-associated transcripts. This is not sufficient to support the stronger concept of deep senescence.
4. The manuscript still implies that EdU-positive cells at 10 dpf in tp53 mutants have escaped senescence, but there is no evidence that they were previously senescent. These cells may simply represent less-damaged hepatocytes, low-UHRF1 cells, or cells that never entered senescence. Lineage tracing or stage scRNA-seq would be needed to solve this question.
5. The Navitoclax interpretation remains too definitive. Reduced SA-β-gal staining and decreased representation of high-EGFP cells do not demonstrate selective killing of high-UHRF1 senescent cells. Without direct evidence of cell death within that specific population, the conclusion that Navitoclax eliminates a defined senescent population remains speculative. The data support a reduction in senescence-associated staining and a reduction in the high-UHRF1-EGFP compartment. They do not demonstrate selective senolytic removal in a mechanistically resolved sense.
6. The increased TUNEL signal in hUHRF1 livers creates an important interpretative problem. The manuscript does not clarify whether apoptotic and senescence-associated populations are distinct, overlapping, or sequentially related. This uncertainty weakens the interpretation of the proposed senescent populations and should be addressed more directly.
Minor point
The manuscript would benefit from shortening, especially where speculative interpretations are repeated.

Referee #2:

The authors have addressed most of my comments. Here are some minor points for the authors to consider.

The additional analysis of scRNA-seq data seems to make sense in my interpretation: Hep2 is most hepatoblastic/immature in control samples, and its abundance is reduced in hUHRF1-expressing samples. Since hepatoblastic/immature features and senescence are often not compatible, to gain an overall consistency, it would be useful to confirm or examine that Hep2 in hUHRF1-expressing samples are less hepatoblastic.

Page 12. "This suggests that while all cells are senescent at this time point, gene expression profiles identify distinct populations". This is an overstatement. Their analysis is mostly based on pseudo-bulk analysis, and it is highly unlikely that "all cells" are senescent.

Fig. 1K labellings of Venn - dark green should be 120 hpf?

** As a service to authors, EMBO Press provides authors with the ability to transfer a manuscript that one journal cannot offer to publish to another journal, without the author having to upload the manuscript data again. To transfer your manuscript to another EMBO Press journal using this service, please click on
Link Not Available

Reviewer 1:

I thank the authors for the revised manuscript that has improved through the addition of new data and clarifications. Nevertheless, my main concern remains unresolved. The manuscript repeatedly converts correlative observations into mechanistic conclusions that are not fully supported by the experiments. This problem affects the interpretation of the scRNAseq data, the definition of senescent states and the conclusions drawn from the Navitoclax treatment. In the current form, the study does not yet support the stronger claims of mechanistically distinct senescent states or selective senolytic removal of a defined senescent population. I therefore recommend major revision.

We present our findings of a functional difference in the cellular response to hUHRF1 overexpression in the context of recent evidence that senescence is heterogeneous, dynamic, and context-dependent [1-4]. The main concern of a correlation between observation and function is refuted based on our hypothesis testing approach to this study. This starts with the observations that hUHRF1 overexpression induces senescence *in vivo*, and that there is heterogeneity in the cell populations. These observations combined with the literature showing that cells in different senescent states have different behaviors [1-6], especially in response to Tp53 manipulation[7] and senolytics[3], we hypothesize that hUHRF1 induces cell populations with different functional outcomes. This is demonstrated in 3 ways:

1. hUHRF1 overexpression in hepatocytes causes senescence *in vivo*, defined by applying standards as recommended by experts in the field as the "minimal information for cellular senescence *in vivo*"[8]. All markers of senescence that are available to use in zebrafish are induced in this model. Therefore, based on this and our previous study we conclude that hUHRF1 overexpression causes senescence, not merely cell stress.
2. Using scRNAseq as the state-of-the-art approach to delineate between cell populations, we identify 4 hepatocyte populations in hUHRF1 overexpressing samples which have different expression of senescence genes. This is complemented by our finding that these different populations are correlated with UHRF1-EGFP mRNA and protein levels. While we agree that this is not a one-to-one correlation between UHRF1 expression and hepatocyte population, and that protein and RNA levels are not always a perfect match, this is a useful surrogate to examine the different behaviors of cells generated by hUHRF1 overexpression.
3. We utilize genetic and pharmacological interventions to test the hypothesis that different cell populations generated by hUHRF1 overexpression have different responses to loss of *tp53* and Navitoclax treatment. We find a highly statistically significant correlation with low UHRF1 levels and proliferation, while those with high UHRF1 levels do not in the conditions studied. This is consistent with other studies, for instance the finding of Beauséjour et al. who showed that senescent cells with low versus high p16 levels respond divergently to p53 inactivation, with only the low-marker population resuming proliferation[7], directly analogous to the differential response we observe based on UHRF1 levels. Whether these high-expressing UHRF1 cells never proliferate is simply too difficult to demonstrate, and we state caveats to the interpretation.

While it can be questioned whether cells marked with high hUHRF1-EGFP protein are the same population of Hep2 cells as defined by scRNAseq, as the correlation is only made based on mRNA expression, it is significant and strong (Figure 5 C-D). Addressing this specifically through profiling cells sorted based on EGFP levels or finding a specific marker of the different cell populations that could be used for staining would be useful, but is experimentally challenging.

Major points

1. The manuscript still equates transcriptional heterogeneity with functionally distinct senescent states. The scRNA-seq analysis identifies heterogeneous hepatocyte profiles, but these profiles do not establish distinct senescent populations in a functional sense. In particular, the interpretation of Hep2 and high-UHRF1 cells as biologically distinct senescent states remains uncertain. There is no direct cell-resolved evidence that these specific populations are SA-β-gal-positive, irreversibly arrested, resistant to proliferative cues, or otherwise functionally senescent. At present, the data support heterogeneity among stressed hepatocytes, not conclusive evidence for distinct senescent states.

We agree that our finding of distinct populations by scRNA-seq alone cannot define functional senescent states, but our interpretation is not based solely on transcriptional heterogeneity. Our findings do not support a model whereby UHRF1 induces stressed, but not senescent, hepatocytes. We deploy, and find positive, every available assay or marker for senescence guidelines from the field [8] which can be used in zebrafish. Each of these assays for senescence are positive in the liver of hUHRF1 larvae at 5 days post fertilization: SA-β-gal staining, hepatocytes do not proliferate despite developmental proliferative cues present in the model, Tp53 activation at the RNA, protein and target level, SASP activation, immune recruitment, and gene signatures used by several groups to identify senescent cells. We agree that this does not define functional heterogeneity, but argue that it is definitive evidence of senescence. In contrast, there is no evidence that the cells are simply “stressed”, a term with no defined marker or phenotypic classification. We agree that this aspect of the work does not does not define functional heterogeneity, but argue that the phenotype is definitively senescence.

scRNA-seq has recently been used to differentiate senescent states, and our manuscript is positioned within these this context [1,2,4]. While we agree that RNA expression does not equate separate functional senescent states, and that a marker to differentiate between populations would be ideal. This does not exist, not only for zebrafish but in the field, as there are no markers that definitively distinguish different senescent populations. Identifying which individual cells carry which specific markers is a separate and distinct challenge from establishing that senescence has occurred. For instance, in hUHRF1 animals at 5 days post fertilization, the whole liver is SA-β-gal-positive, but because this is a diffuse stain it cannot resolve individual cells within the tissue, so a cell-by-cell correlation between this stain and another senescence marker is not feasible. We instead use UHRF1-EGFP protein levels as a surrogate marker for different cell populations to distinguish between the different populations. This is not an absolute correlation with the different senescent states, but it is a strong one, and it provides genuine insight into the functional relevance of these populations.

When integrated with the tissue-level senescence assays, proliferation data, *tp53* pathway interrogation using genetics and pharmacologic approaches, the data support the conclusion that hUHRF1 induces heterogeneous senescence-associated hepatocyte states in vivo. This interpretation is consistent with recent work showing that senescence can comprise multiple transcriptional and functional states *in vivo* [6].

2. The DDR section continues to present an inferred model as if it were experimentally resolved. γH2AX and p-RPA are compatible with DNA damage and/or replication stress, but they do not establish the specific sequence proposed by the authors. The manuscript still implies that UHRF1 first induces double-strand breaks and subsequently single-stranded DNA formation. This order is not demonstrated. The data support the presence of DNA damage response markers, but they do not define the initiating lesion, the pathway hierarchy, or the causal progression of the response.

We agree that γH2AX and phospho-RPA do not, by themselves, define the initiating DNA lesion. However, we present data showing a sequence of events whereby γH2AX is detected first (at 80 hours post fertilization) and phospho-RPA next (at 96 hours post fertilization). This is coupled with the activation of the DDR by RNAseq at 80 hours post fertilization. Together this indicates that DNA damage is an early result of hUHRF1 overexpression in this model, and are In the revised manuscript we modified our description to explicitly present this as a proposed model rather than a resolved mechanism: the revised text states that the data *suggest* DSB formation followed by single-stranded DNA formation, while also acknowledging that phospho-RPA may reflect ongoing DNA repair, or that multiple mechanisms of DNA damage may occur depending on hUHRF1 levels or cell-cycle stage. This conclusion is strengthened by our functional data showing that, *atm* the major responding factor to double-strand breaks, is required for UHRF1-induced SA-β-gal. Together, our data showing induction of a well established marker of DNA double stranded breaks (Figure 2A), activation of the doublestrand break pathway (Figure 1G) and that *atm* is required for a canonical senescence marker (Figure 2B), supports the conclusion that hUHRF1 overexpression causes double stranded breaks. While we acknowledge that we do not provide definitive evidence for the initiating lesion, this is not central to the manuscript, and does not alter the conclusion that hUHRF1 overexpression induces a robust DNA damage response associated with hepatocyte senescence *in vivo.*

3. The term "deep senescence" remains insufficiently supported. The manuscript does not demonstrate irreversible arrest, persistence of the state over time, stability after removal of stress, or failure to respond to regenerative stimuli. The unsuccessful regeneration experiment described in the response cannot be used as supporting evidence. At most, the data identify high-UHRF1, non-proliferative cells enriched for senescence-associated transcripts. This is not sufficient to support the stronger concept of deep senescence.

The reviewer referred to a term used in a previous version of the manuscript evaluated at *EMBO J*. We did not use the term “deep senescence” in the revised version of the manuscript to describe the hUHRF1-induced senescence, and therefore this critique is not relevant to the current version of the manuscript evaluated for *EMBO Reports*.

4. The manuscript still implies that EdU-positive cells at 10 dpf in tp53 mutants have escaped senescence, but there is no evidence that they were previously senescent. These cells may simply represent less-damaged hepatocytes, low-UHRF1 cells, or cells that never entered senescence. Lineage tracing or stage scRNA-seq would be needed to solve this question.

We agree that lineage tracing could prove definitively that EdU-positive cells at 10 dpf in tp53 mutants were previously senescent or that scRNAseq in *tp53* mutants would identify how this intervention shifted the senescent populations. Since there is no marker that enables labeling senescent cells and tracking their fate and the time and resource investment required for another scRNAseq experiment is prohibitive, we have instead based our conclusion on the combined evidence that hUHRF1 overexpression induces senescence at 5 dpf, and that the later reappearance of EdU-positive hepatocytes in *tp53* mutants is consistent with cell-cycle re-entry of senescent cells, consistent with recent literature showing that senescence is escapable [9,10]. However, in the revised version, we framed this result as suggestive, not conclusive, of senescence escape.

The important part of this finding is that we never see cells with the highest levels of hUHRF1 re-enter the cell cycle, even when tp53 is removed. This is evidence that hUHRF1 levels matter, these levels distinguish functional capacity, and using high hUHRF1 as a surrogate marker for Hep2 is consistent with a model whereby UHRF1 levels influence whether cells can proliferate, similar to other studies with Ras [6], Myc [11] and p21[5].

5. The Navitoclax interpretation remains too definitive. Reduced SA-β-gal staining and decreased representation of high-EGFP cells do not demonstrate selective killing of high-UHRF1 senescent cells. Without direct evidence of cell death within that specific population, the conclusion that Navitoclax eliminates a defined senescent population remains speculative. The data support a reduction in senescence-associated staining and a reduction in the high-UHRF1-EGFP compartment. They do not demonstrate selective senolytic removal in a mechanistically resolved sense.

Navitoclax is expected to induce death of senescent cells. Our finding that Navitoclax reduces both SA-β-gal staining and cells with high hUHRF1 levels provides a logical foundation to the conclusion that Navitoclax targets senescent cells with high hUHRF1 levels. An alternative explanation such as Navitoclax altering UHRF1 expression, is speculative and, while it could explain the decrease in senescence, is not the more logical interpretation of the data. We recognize that performing TUNEL would address this concern directly, but we do not believe this point justifies rejection of the paper.

6. The increased TUNEL signal in hUHRF1 livers creates an important interpretative problem. The manuscript does not clarify whether apoptotic and senescence-associated populations are distinct, overlapping, or sequentially related. This uncertainty weakens the interpretation of the proposed senescent populations and should be addressed more directly.

We detect apoptosis markers in a small population of cells, and therefore apoptosis is not the dominant cellular state captured in the dataset. In contrast, the majority of cells express senescence markers. Therefore, even if there is overlap, it would be confined to a small subset. Additional analysis to directly overlap these two markers in scRNAseq data is straightforward, making and is addressable in a minor revision.

Minor point

The manuscript would benefit from shortening, especially where speculative interpretations are repeated.

This is addressable in a minor revision.

Referee #2:

The authors have addressed most of my comments. Here are some minor points for the authors to consider.

The additional analysis of scRNA-seq data seems to make sense in my interpretation: Hep2 is most hepatoblastic/immature in control samples, and its abundance is reduced in hUHRF1-expressing samples. Since hepatoblastic/immature features and senescence are often not compatible, to gain an overall consistency, it would be useful to confirm or examine that Hep2 in hUHRF1-expressing samples are less hepatoblastic.

We agree that this is interesting and that the hepatoblastic or immature features of Hep2 are relevant. We are currently preparing a separate manuscript focused specifically on how hUHRF1 influences hepatoblastic/immature features and how these contribute to tumorigenesis. To avoid overextending the present study, we have limited the interpretation here to the senescence-associated phenotype.

Page 12. "This suggests that while all cells are senescent at this time point, gene expression profiles identify distinct populations". This is an overstatement. Their analysis is mostly based on pseudo-bulk analysis, and it is highly unlikely that "all cells" are senescent.

This is a minor edit which can modify the text to “These data suggest that hUHRF1-expressing hepatocytes broadly acquire senescence-associated features at this time point, while gene-expression profiles reveal heterogeneity within this response”.

Fig. 1K labellings of Venn - dark green should be 120 hpf?

This is a minor edit.

References

1. Tao, W., Yu, Z., and Han, J.J. (2024). Single-cell senescence identification reveals senescence heterogeneity, trajectory, and modulators. Cell Metab *36*, 1126-1143.e1125. 10.1016/j.cmet.2024.03.009.
2. Fernandez, B., Passanisi, V.J., Ashraf, H.M., and Spencer, S.L. (2025). Single-cell RNA sequencing reveals a quiescence-senescence continuum and distinct senotypes following chemotherapy. Nature Communications *17*, 169. 10.1038/s41467-025-66836-z.
3. Kamat, P., Macaluso, N., Li, Y., Agrawal, A., Winston, A., Pan, L., Stewart, T., Starich, B., Milcik, N., Min, C., et al. (2025). Single-cell morphology encodes functional subtypes of senescence in aging human dermal fibroblasts. Sci Adv *11*, eads1875. 10.1126/sciadv.ads1875.
4. Sanborn, M.A., Wang, X., Gao, S., Dai, Y., and Rehman, J. (2025). Unveiling the cell-type-specific landscape of cellular senescence through single-cell transcriptomics using SenePy. Nature Communications *16*, 1884. 10.1038/s41467-025-57047-7.
5. Hsu, C.H., Altschuler, S.J., and Wu, L.F. (2019). Patterns of Early p21 Dynamics Determine Proliferation-Senescence Cell Fate after Chemotherapy. Cell *178*, 361-373 e312. 10.1016/j.cell.2019.05.041.
6. Chan, A.S.L., Zhu, H., Narita, M., Cassidy, L.D., Young, A.R.J., Bermejo-Rodriguez, C., Janowska, A.T., Chen, H.C., Gough, S., Oshimori, N., et al. (2024). Titration of RAS alters senescent state and influences tumour initiation. Nature *633*, 678-685. 10.1038/s41586-024-07797-z.
7. Beausejour, C.M., Krtolica, A., Galimi, F., Narita, M., Lowe, S.W., Yaswen, P., and Campisi, J. (2003). Reversal of human cellular senescence: roles of the p53 and p16 pathways. EMBO J *22*, 4212-4222. 10.1093/emboj/cdg417.
8. Ogrodnik, M., Carlos Acosta, J., Adams, P.D., d'Adda di Fagagna, F., Baker, D.J., Bishop, C.L., Chandra, T., Collado, M., Gil, J., Gorgoulis, V., et al. (2024). Guidelines for minimal information on cellular senescence experimentation in vivo. Cell *187*, 4150-4175. 10.1016/j.cell.2024.05.059.
9. Martínez-Zamudio, R.I., Stefa, A., Nabuco Leva Ferreira Freitas, J.A., Vasilopoulos, T., Simpson, M., Doré, G., Roux, P.-F., Galan, M.A., Chokshi, R.J., Bischof, O., and Herbig, U. (2023). Escape from oncogene-induced senescence is controlled by POU2F2 and memorized by chromatin scars. Cell Genomics *3*. 10.1016/j.xgen.2023.100293.
10. Patel, P.L., Suram, A., Mirani, N., Bischof, O., and Herbig, U. (2016). Derepression of <i>hTERT</i> gene expression promotes escape from oncogene-induced cellular senescence. Proceedings of the National Academy of Sciences *113*, E5024-E5033. doi:10.1073/pnas.1602379113.
11. Afifi, M.M., Crncec, A., Cornwell, J.A., Cataisson, C., Paul, D., Ghorab, L.M., Hernandez, M.O., Wong, M., Kedei, N., and Cappell, S.D. (2023). Irreversible cell cycle exit associated with senescence is mediated by constitutive MYC degradation. Cell Rep *42*, 113079. 10.1016/j.celrep.2023.113079.

Authors' Point-by-point response to feedback received from Referee #1 on the Appeal

Thank you for sharing the authors' appeal. Several issues are resolved, one concern I withdraw (the "deep senescence" of point 3), but one key claim remains experimentally unsupported, and I would not concede it based on the argumentation alone.

We thank the reviewer for positively considering our appeal and for suggesting experiments that added more detail to this study and text modifications that better contextualized our findings.

Solved:
- The DNA-damage sequence addressed in point 2 is now adequately handled by reframing it as a proposed model supported by ATM dependence.
- The interpretation of 10 dpf EdU^{+} cells in point 4 is acceptable if softened to "suggestive" and not reiterated strongly elsewhere.
- The authors' more robust negative result-that the highest-UHRF1 cells never re-enter the cycle, even in the absence of tp53 is the defensible claim.

To be solved:
- For point 1, I think a textual clarification is sufficient. The functional claim that UHRF1 level stratifies proliferative fate, as shown in Fig. 5K-L, is cell-resolved and convincing. However, the claim that the Hep2 cluster represents that state rests on an mRNA alone and therefore remains inferential. The text should state the correspondence as proposed rather than demonstrated. No new data are required for this point.

Text has been modified to clarify that Hep2 cells are a proposed senotype based on transcriptomic data and that hUHRF1-EGFP levels are proposed as a surrogate marker with the direct relationship between these two identities remaining to be clarified.

- Point 5 remains unresolved and, in my view, decisive. Selective senolytic removal has not been established. The co-loss of SA-β-gal signal and high-EGFP cells is consistent with senolysis, but it does not demonstrate cell death of that population. Excluding alternative explanations is on the authors' side. Since they acknowledge that TUNEL would resolve this issue, and they already use TUNEL elsewhere in the manuscript (e.g. Appendix Fig. S4), I would think an easy proposal to provide data on TUNEL/EGFP co-staining in Navitoclax treated cells.

To address the efficacy of Navitoclax to induce apoptosis in this system, we performed TUNEL assay on DMSO and Navitoclax-treated *hUHRF1* and controls. This showed a significant increase of TUNEL-positive cells in *hUHRF1* samples (**NEW Figure 6E**). To test the hypothesis that Navitoclax is targeting hUHRF1-EGFP expressing cells, we assessed the overlap of TUNEL and EGFP. This showed negligible overlap between TUNEL and EGFP in DMSO treated hUHRF1 larvae, but in Navitoclax treated larvae, 30% of TUNEL+ cells were also EGFP+ (**NEW Figure 6E**). This shows that Navitoclax induces cell death in hUHRF1-EGFP expressing cells, but since EGFP levels are diminished during sample processing for TUNEL, we cannot directly correlate EGFP levels with TUNEL labeling.

Since Navitoclax causes hUHRF1-EGFP cells to undergo apoptosis and cells with the highest level of hUHRF1-EGFP are not present after Navitoclax treatment, deductive reasoning leads to the conclusion that Navitoclax induces senolysis in cells with the highest UHRF1 levels.

- For point 6, the apoptosis/senescence overlap should be addressed by co-mapping both markers in the existing scRNAseq dataset. This analysis appears feasible with data already in

the manuscript and should be included.

We addressed this by extending the data presented in the previous version by performing additional analysis of the scRNAseq data as recommended. The new data are shown **in NEW Appendix Figure S4C, D, E, F**. We show that the apoptotic cells are a minor population that is not enriched in any distinct senotype, and that there is no overlap between senescent cells and apoptotic cells. We did this by first analyzing the expression of genes involved in apoptosis in cells which we categorized as senescent based on a senescent score (SenScore >0). We next defined an Apoptosis Score and then compared the expression with cells with a positive and negative SenScore as UMAP as well as through a correlation plot that directly compares each cell based on the SenScore and Apoptosis Score. This shows that there is no correlation between the two.

Overall, the core of the manuscript is novel and of interest to a broad audience. If your policy can accommodate it, I would support one further revision limited to point 5 (experimental), point 6 (reanalysis), and point 1 rewording. I could not endorse the selective-senolysis claim without the point 5 data, but I do not regard the remaining issues as grounds for rejection.

Dear Kirsten,

Thank you once more for the submission of your revised manuscript to EMBO reports. I have already forwarded you the referee report and am now following up with the formal decision letter.

As you will see from the report below, former referee #1 considers the revised version much improved and recommends publication pending minor textual changes.

From the editorial side, there are also a few things that we need before we can proceed with the official acceptance of your study.

- Summary should be Abstract.

- Material and Methods should be Methods

- Regarding the Author Contributions, we use CRediT to specify the contributions of each author in the journal submission system. Therefore, please remove the Author Contributions from the manuscript file and make sure that the author contributions in our online manuscript tracking system are correct and up-to-date. The information you specified in the system will be automatically retrieved and typeset into the article. You can enter additional information in the free text box provided, if you wish. See also our guide to authors https://www.embopress.org/page/journal/14693178/authorguide#authorshipguidelines.

- Author Checklist: please add the manuscript ID (line 6)

- Please describe your findings in the abstract in present tense.

- Iwasaki et al 2026; Madakashira et al, 2023; Sur et al 2023 are preprints and need to be cited as follows:
* in the text: (preprint: AUTHOR name et al, 2026)
* in the reference list: add [PREPRINT] at the end of the reference.

- Funding information in the online manuscript tracking system: the text/funders provided in the Comments box need to be removed as each funder acknowledged in the manuscript needs to be provided as a separate entry in the system. Please make sure that the information in the manuscript and in the system is congruent. The one in the system is eventually submitted to Pubmed.

- Fig. 1L is called out on page 7, but it is not labeled in the figure file nor listed in the figure legend. Please check and correct.

- Figure 6G misses a callout in the text.

- The Supplemental tables S1, S3, S4 are complex datasets and need to be updated to Dataset EV1 - EV3 in all places; the sheet name in Appendix Table S4 needs updating to Dataset EV3 (currently titled as Supplemental Table S3); the legends of these datasets need to be removed from the manuscript file and each should be provided in its corresponding Excel file as a separate sheet/tab.

- Supplemental tables S2 and S5 can be uploaded as Table EV1 and EV2 and should also have a legend in the .xls file.

- Please move the Supplementary Materials and Methods to the main methods section.

- The Data Availability statement needs to be placed before Acknowledgments. We also need the specific URLs for datasets (GEO: GSE302812, GSE173792, GSE303361).

- Please provide the synopsis image in jpeg, TIFF or png format: exactly 550 pixels wide x 300 pixels high.

- The short summary text in the manuscript file needs to be removed and uploaded separately as Synopsis text with 3-4 bullet points describing the key findings.

- Please address the following points pertaining to the figure legends:

*) Figure EV1: the panel descriptions are incorrect for C and D.

*) Please provide the exact p values in the legends of 1B,C,D,J; 2A,B; 3A,B,E; 5D,J,K,L;
6B,D,E,F; Appendix Figure S4A

*) Please indicate the statistical test used for data analysis in the legends of figures 1E,F; 3C; 4D,G; EV-1A,B,C; Appendix Figure S1-B,C,E; Appendix Figure S4F

*) Please note that information related to n is missing in the legends of 1D,E; 2A,B; 3A,B,E; 5A; EV-1A; EV-2B; Appendix Figure S1B,D,E; Appendix Figure S3-B,C; Appendix Figure S4-A,B,F.

*) Please note that the error bars are not defined in the legends of figures 3A; Appendix Figure S1-E.

*) Please note that the scale bar needs to be defined for figures 2A,B; 5G; 6F; Appendix Figure S6-B.

*) Please note that scale bar and its definition are missing for figures 1A,B,D,I; 5G; 6C,E; Appendix Figure S4A; Appendix Figure S6B.

*) Please note that the yellow asterisk is not defined in the legend of figure Appendix Figure S6-B. This needs to be rectified. The pink arrow needs to be defined for Appendix Figure S1D.

*) Appendix Figure S4A:. The significance value in the legend (**) does not match the one shown in the panel (*).

- We perform a spot check on the supplied source data and I noticed for Figure 1A that the Control for 72 hpf is missing. You supplied 80 hrs instead.
- The control images for Figure 1B seem not to match the one shown in the figure panel. Please also indicate that you changed the background to a uniform white for these images.
- Figure 1I Control mpeg1: the images in the figure panel and the source data seem not to match.
Please carefully check all source data images provided for consistency.

I look forward to seeing a new revised version of your manuscript as soon as possible.

With kind regards,

Martina

Martina Rembold, PhD
Senior Editor
EMBO reports

===================

Referee #1:

I thank the authors for a thorough revision. My remaining concerns are now adequately addressed. I recommend acceptance subject to the minor textual modifications noted below; no further experiments are required.
Point 1 (Hep2 correspondence). Presenting Hep2 as a proposed senotype and hUHRF1-EGFP as a proposed surrogate marker, with the direct correspondence left open, resolves my concern. This was a matter of framing rather than data, and the revised wording is appropriate.
Point 5 (selective senolysis). This was my decisive concern, and the new Figure 6 satisfies it. The evidence is now convergent rather than correlational: navitoclax increases apoptosis in hUHRF1 but not in controls (a built-in control against non-specific toxicity), the induced apoptosis co-localizes with hUHRF1-EGFP, and the high-intensity population is depleted. Together these exclude the alternatives I mentioned (off-target toxicity and marker downregulation). I accept that specificity for the highest-UHRF1 cells is inferred rather than measured cell-by-cell, given EGFP attenuation during processing, provided this limitation is stated. My one request is textual: the claim should rest explicitly on this convergent evidence rather than on the single co-localization statistic, and the phrase "deductive reasoning" should be replaced with a statement that senolysis is the best-supported interpretation.
Point 6 (apoptosis/senescence overlap). The reanalysis (Appendix Fig. S4C-F) shows apoptotic and senescent cells do not overlap at baseline ($R = 0.0077$, $p = 0.59$). This is consistent with the senolysis result, since senescent cells resist apoptosis until BCL-2-family inhibition. A sentence making this link explicit would be helpful, but the point is resolved.
The core findings are novel and of broad interest, and the manuscript is now suitably supported. I recommend acceptance pending the text modifications above.

All editorial suggestions have been addressed as described below each point in blue.

Editorial Comments

1. Summary should be Abstract.

Done.

2. Material and Methods should be Methods

Done.

3. Regarding the Author Contributions, we use CRediT to specify the contributions of each author in the journal submission system. Therefore, please remove the Author Contributions from the manuscript file and make sure that the author contributions in our online manuscript tracking system are correct and up-to-date. The information you specified in the system will be automatically retrieved and typeset into the article. You can enter additional information in the free text box provided, if you wish. See also our guide to authors https://www.embopress.org/page/journal/14693178/authorguide#authorshipguidelines.

Done.

4. Author Checklist: please add the manuscript ID (line 6)

Done.

5. Please describe your findings in the abstract in present tense.

Done.

6. Iwasaki et al 2026; Madakashira et al, 2023; Sur et al 2023 are preprints and need to be cited as follows:
* in the text: (preprint: AUTHOR name et al, 2026)
* in the reference list: add [PREPRINT] at the end of the reference.

Some of these have been published and we have updated to the published references Madakashira et al, and Sur et al. We changed the reference as described for the preprint of Iwasaki et al.

7. Funding information in the online manuscript tracking system: the text/funders provided in the Comments box need to be removed as each funder acknowledged in the manuscript needs to be provided as a separate entry in the system. Please make sure that the information in the manuscript and in the system is congruent. The one in the system is eventually submitted to Pubmed.

Done.

8. Fig. 1L is called out on page 7, but it is not labeled in the figure file nor listed in the figure legend. Please check and correct.

Reference to Figure 1L is corrected to Figure 1K.

9. Figure 6G misses a callout in the text.

We now listed this in the text.

10. The Supplemental tables S1, S3, S4 are complex datasets and need to be updated to Dataset EV1, EV3 in all places; the sheet name in Appendix Table S4 needs updating to Dataset EV3 (currently titled as Supplemental Table S3); the legends of these datasets need to be removed from the manuscript file and each should be provided in its corresponding Excel file as a separate sheet/tab.

Done.

11. Supplemental tables S2 and S5 can be uploaded as Table EV1 and EV2 and should also have a legend in the .xls file.

Done.

12. Please move the Supplementary Materials and Methods to the main methods section.

Done.

13. The Data Availability statement needs to be placed before Acknowledgments. We also need the specific URLs for datasets (GEO: GSE302812, GSE173792, GSE303361).

All datasets have been publicly released and URLs provided.

14. Please provide the synopsis image in jpeg, TIFF or png format: exactly 550 pixels wide x 300 pixels high.

Done.

15. The short summary text in the manuscript file needs to be removed and uploaded separately as Synopsis text with 3-4 bullet points describing the key findings.

Done.

16. Please address the following points pertaining to the figure legends:

*) Figure EV1: the panel descriptions are incorrect for C and D.

Corrected.

*) Please provide the exact p values in the legends of 1B, C, D, J; 2A, B; 3A, B, E; 5D, J, K, L; 6B, D, E, F; Appendix Figure S4A.

Done.

*) Please indicate the statistical test used for data analysis in the legends of figures 1E, F; 3C; 4D, G; EV-1A, B,C; Appendix Figure S1-B, C, E; Appendix Figure S4F

Done, except for S1C since we didn't perform statistical test in this panel

*) Please note that information related to n is missing in the legends of 1D, E; 2A, B; 3A, B, E; 5A; EV-1A; EV-2B; Appendix Figure S1B, D, E; Appendix Figure S3-B, C; Appendix Figure S4-A, B, F.

Done, except for all of the violin plots containing cells extracted from scRNAseq as this does not represent the number of replicates but instead is the number of cells in each population (as shown in Figure 4F) and therefore the n for these figures is not relevant.

*) Please note that the error bars are not defined in the legends of figures 3A; Appendix Figure S1-E.

Done.

*) Please note that the scale bar needs to be defined for figures 2A, B; 5G; 6F; Appendix Figure S6-B.

Done.

*) Please note that scale bar and its definition are missing for figures 1A, B, D, I; 5G; 6C, E; Appendix Figure S4A; Appendix Figure S6B.

Done.

*) Please note that the yellow asterisk is not defined in the legend of figure Appendix Figure S6-B. This needs to be rectified. The pink arrow needs to be defined for Appendix Figure S1D.

Done.

*) Appendix Figure S4A: The significance value in the legend (**) does not match the one shown in the panel (*).

Done. The p-value was incorrect in the previous version and we have rectified this in both the panel and in the figure legend.

16. We perform a spot check on the supplied source data and I noticed for Figure 1A that the Control for 72 hpf is missing. You supplied 80 hrs instead.

Updated with 72 hpf control.

17. The control images for Figure 1B seem not to match the one shown in the figure panel. Please also indicate that you changed the background to a uniform white for these images.

Done.

18. Figure 1I Control mpeg1: the images in the figure panel and the source data seem not to match.
Please carefully check all source data images provided for consistency.

Updated source data.

====================

Referee #1:

I thank the authors for a thorough revision. My remaining concerns are now adequately addressed. I recommend acceptance subject to the minor textual modifications noted below; no further experiments are required.

We are grateful for the reviewer for the positive feedback about the revision.

Point 1 (Hep2 correspondence). Presenting Hep2 as a proposed senotype and hUHRF1-EGFP as a proposed surrogate marker, with the direct correspondence left open, resolves my concern. This was a matter of framing rather than data, and the revised wording is appropriate.

This indicates that this point is resolved.

Point 5 (selective senolysis). This was my decisive concern, and the new Figure 6 satisfies it. The evidence is now convergent rather than correlational: navitoclax increases apoptosis in hUHRF1 but not in controls (a built-in control against non-specific toxicity), the induced apoptosis co-localizes with hUHRF1-EGFP, and the high-intensity population is depleted. Together these exclude the alternatives I mentioned (off-target toxicity and marker downregulation). I accept that specificity for the highest-UHRF1 cells is inferred rather than measured cell-by-cell, given EGFP attenuation during processing, provided this limitation is

stated. My one request is textual: the claim should rest explicitly on this convergent evidence rather than on the single co-localization statistic, and the phrase "deductive reasoning" should be replaced with a statement that senolysis is the best-supported interpretation.

The term “deductive reasoning” was use only in the response to the reviewers, and not in the manuscript and therefore this is not relevant to the manuscript. We clarified in the current version of the manuscript that “This showed that Navitoclax treatment significantly reduced hUHRF1-EGFP heterogeneity and cells with the highest level of hUHRF1-EGFP were no longer detected in Navitoclax treated samples (Figure 6F)”

Point 6 (apoptosis/senescence overlap). The reanalysis (Appendix Fig. S4C-F) shows apoptotic and senescent cells do not overlap at baseline ($R = 0.0077$, $p = 0.59$). This is consistent with the senolysis result, since senescent cells resist apoptosis until BCL-2-family inhibition. A sentence making this link explicit would be helpful, but the point is resolved.

Done.

The core findings are novel and of broad interest, and the manuscript is now suitably supported. I recommend acceptance pending the text modifications above.

We are grateful for the positive comments and are pleased by this outcome.

Prof. Kirsten Sadler
NYU Abu Dhabi
Program in Biology
PO Box 129188
Abu Dhabi, Abu Dhabi 0000
United Arab Emirates

Dear Kirsten,

I am very pleased to accept your manuscript for publication in the next available issue of EMBO reports. Thank you for your contribution to our journal.

Your manuscript will be processed for publication by EMBO Press. It will be copy edited and you will receive page proofs prior to publication. Please note that you will be contacted by Springer Nature Author Services to complete licensing and payment information.

You may qualify for financial assistance for your publication charges - either via a Springer Nature fully open access agreement or an EMBO initiative. Check your eligibility: https://link.springer.com/journal/44319/how-to-publish-with-us

Should you be planning a Press Release on your article, please notify the production team via this form in order to coordinate publication and release dates: https://forms.cloud.microsoft/e/eH4A8JVP6g

If you have any questions, please do not hesitate to contact the Editorial Office. Thank you for your contribution to EMBO Reports.

Kind regards,

Martina

Martina Rembold, PhD
Senior Editor
EMBO reports

>>> Please note that it is EMBO Reports policy for the transcript of the editorial process (containing referee reports and your response letter) to be published as an online supplement to each paper. If you do NOT want this, you will need to inform the Editorial Office via email immediately. More information is available here: https://link.springer.com/partners/embo-press/editorial-policies#Peer%20review

EMBO Press Author Checklist

Corresponding Author Name: Kirsten C. Sadler	
Journal Submitted to: EMBO Report	
Manuscript Number: EMBOR-2026-63714V4	

USEFUL LINKS FOR COMPLETING THIS FORM
The EMBO Journal - Author Guidelines
EMBO Reports - Author Guidelines
Molecular Systems Biology - Author Guidelines
EMBO Molecular Medicine - Author Guidelines

Reporting Checklist for Life Science Articles (updated January
This checklist is adapted from Materials Design Analysis Reporting (MDAR) Checklist for Authors. MDAR establishes a minimum set of requirements in transparent reporting in the life sciences (see Statement of Task: 10.31222/osf.io/9sm4x). Please follow the journal's guidelines in preparing your
Please note that a copy of this checklist will be published alongside your article.

Abridged guidelines for figures
1. Data
The data shown in figures should satisfy the following conditions:
- the data were obtained and processed according to the field's best practice and are presented to reflect the results of the experiments in an accurate and unbiased manner.
- ideally, figure panels should include only measurements that are directly comparable to each other and obtained with the same assay.
- plots include clearly labeled error bars for independent experiments and sample sizes. Unless justified, error bars should not be shown for technical
- if n<5, the individual data points from each experiment should be plotted. Any statistical test employed should be justified.
- Source Data should be included to report the data underlying figures according to the guidelines set out in the authorship guidelines on Data

2. Captions
Each figure caption should contain the following information, for each panel where they are relevant:
- a specification of the experimental system investigated (eg cell line, species name).
- the assay(s) and method(s) used to carry out the reported observations and measurements.
- an explicit mention of the biological and chemical entity(ies) that are being measured.
- an explicit mention of the biological and chemical entity(ies) that are altered/varied/perturbed in a controlled manner.
- the exact sample size (n) for each experimental group/condition, given as a number, not a range;
- a description of the sample collection allowing the reader to understand whether the samples represent technical or biological replicates (including how many animals, litters, cultures, etc.).
- a statement of how many times the experiment shown was independently replicated in the laboratory.
- definitions of statistical methods and measures:
  - common tests, such as t-test (please specify whether paired vs. unpaired), simple χ2 tests, Wilcoxon and Mann-Whitney tests, can be unambiguously identified by name only, but more complex techniques should be described in the methods section;
  - are tests one-sided or two-sided?
  - are there adjustments for multiple comparisons?
  - exact statistical test results, e.g., P values = x but not P values < x;
  - definition of 'center values' as median or average;
  - definition of error bars as s.d. or s.e.m.

Please complete ALL of the questions below.
Select "Not Applicable" only when the requested information is not relevant for your study.

Materials

Newly Created Materials	Information included in the manuscript?	In which section is the information available? (Reagents and Tools Table, Materials and Methods, Figures, Data Availability Section)
New materials and reagents need to be available; do any restrictions apply?	Not Applicable	

Antibodies	Information included in the manuscript?	In which section is the information available? (Reagents and Tools Table, Materials and Methods, Figures, Data Availability Section)
For **antibodies** provide the following information:<br>- Commercial antibodies: RRID (if possible) or supplier name, catalogue number and or/clone number<br>- Non-commercial: RRID or citation	Yes	Reagents and Tools Table

DNA and RNA sequences	Information included in the manuscript?	In which section is the information available? (Reagents and Tools Table, Materials and Methods, Figures, Data Availability Section)
**Short novel DNA or RNA including primers, probes**: provide the sequences.	Not Applicable	

Cell materials	Information included in the manuscript?	In which section is the information available? (Reagents and Tools Table, Materials and Methods, Figures, Data Availability Section)
**Cell lines:** Provide species information, strain. Provide accession number in repository **OR** supplier name, catalog number, clone number, and/**OR** RRID.	Not Applicable	
**Primary cultures:** Provide species, strain, sex of origin, genetic modification status.	Not Applicable	
Report if the cell lines were recently **authenticated** (e.g., by STR profiling) and tested for mycoplasma contamination.	Not Applicable	

Experimental animals	Information included in the manuscript?	In which section is the information available? (Reagents and Tools Table, Materials and Methods, Figures, Data Availability Section)
**Laboratory animals or Model organisms:** Provide species, strain, sex, age, genetic modification status. Provide accession number in repository **OR** supplier name, catalog number, clone number, **OR** RRID.	Yes	Reagents and Tools Table, Materials and Methods
**Animal observed in or captured from the field:** Provide species, sex, and age where possible.	Not Applicable	
Please detail **housing and husbandry conditions**.	Yes	Materials and Methods

Plants and microbes	Information included in the manuscript?	In which section is the information available? (Reagents and Tools Table, Materials and Methods, Figures, Data Availability Section)
**Plants:** provide species and strain, ecotype and cultivar where relevant, unique accession number if available, and source (including location for collected wild specimens).	Not Applicable	
**Microbes:** provide species and strain, unique accession number if available, and source.	Not Applicable	

Human research participants	Information included in the manuscript?	In which section is the information available? (Reagents and Tools Table, Materials and Methods, Figures, Data Availability Section)
If collected and within the bounds of privacy constraints report on age, sex and gender or ethnicity for all study participants.	Not Applicable	

Core facilities	Information included in the manuscript?	In which section is the information available? (Reagents and Tools Table, Materials and Methods, Figures, Data Availability Section)
If your work benefited from core facilities, was their service mentioned in the acknowledgments section?	Yes	Acknowledgments

Design

Study protocol	Information included in the manuscript?	In which section is the information available? (Reagents and Tools Table, Materials and Methods, Figures, Data Availability Section)
If study protocol has been **pre-registered, provide DOI in the manuscript**. For clinical trials, provide the trial registration number **OR** cite DOI.	Not Applicable	
Report the **clinical trial registration number** (at ClinicalTrials.gov or equivalent), where applicable.	Not Applicable	

Laboratory protocol	Information included in the manuscript?	In which section is the information available? (Reagents and Tools Table, Materials and Methods, Figures, Data Availability Section)
Provide DOI OR other citation details if **external detailed step-by-step protocols** are available.	Not Applicable	

Experimental study design and statistics	Information included in the manuscript?	In which section is the information available? (Reagents and Tools Table, Materials and Methods, Figures, Data Availability Section)
Include a statement about **sample size** estimate even if no statistical methods were used.	Yes	Materials and Methods
Were any steps taken to minimize the effects of subjective bias when allocating animals/samples to treatment (e.g. **randomization procedure**)? If yes, have they been described?	Yes	Materials and Methods
Include a statement about **blinding** even if no blinding was done.	Yes	Materials and Methods
Describe **inclusion/exclusion criteria** if samples or animals were excluded from the analysis. Were the criteria pre-established? If sample or data points were omitted from analysis, report if this was due to attrition or intentional exclusion and provide justification.	Not Applicable	
For every figure, are **statistical tests** justified as appropriate? Do the data meet the assumptions of the tests (e.g., normal distribution)? Describe any methods used to assess it. Is there an estimate of variation within each group of data? Is the variance similar between the groups that are being statistically compared?	Yes	Figure legends, Materials and Methods

Sample definition and in-laboratory replication	Information included in the manuscript?	In which section is the information available? (Reagents and Tools Table, Materials and Methods, Figures, Data Availability Section)
In the figure legends: state number of times the experiment was **replicated** in laboratory.	Yes	Figure legends, Materials and Methods
In the figure legends: define whether data describe **technical or biological replicates**.	Yes	Figure legends, Materials and Methods

Ethics

Ethics	Information included in the manuscript?	In which section is the information available? (Reagents and Tools Table, Materials and Methods, Figures, Data Availability Section)
Studies involving **human participants**: State details of **authority granting ethics approval** (IRB or equivalent committee(s), provide reference number for approval.	Not Applicable	
Studies involving **human participants**: Include a statement confirming that **informed consent** was obtained from all subjects and that the experiments conformed to the principles set out in the WMA Declaration of Helsinki and the Department of Health and Human Services Belmont Report.	Not Applicable	
Studies involving **human participants:** For publication of **patient photos**, include a statement confirming that consent to publish was obtained.	Not Applicable	
Studies involving experimental **animals**: State details of **authority granting ethics approval** (IRB or equivalent committee(s), provide reference number for approval. Include a statement of compliance with ethical regulations.	Yes	Materials and Methods
Studies involving **specimen and field samples:** State if relevant **permits** obtained, provide details of authority approving study; if none were required, explain why.	Not Applicable	

Dual Use Research of Concern (DURC)	Information included in the manuscript?	In which section is the information available? (Reagents and Tools Table, Materials and Methods, Figures, Data Availability Section)
Could your study fall under dual use research restrictions? Please check biosecurity documents and list of **select agents and toxins** (CDC): https://www.selectagents.gov/sat/list.htm	Not Applicable	
If you used a select agent, is the security level of the lab appropriate and reported in the manuscript?	Not Applicable	
If a study is subject to dual use research of concern regulations, is the name of the **authority granting approval and reference number** for the regulatory approval provided in the manuscript?	Not Applicable	

Reporting

The MDAR framework recommends adoption of discipline-specific guidelines, established and endorsed through community initiatives. Journals have their own policy about requiring specific guidelines and recommendations to complement MDAR.

Adherence to community standards	Information included in the manuscript?	In which section is the information available? (Reagents and Tools Table, Materials and Methods, Figures, Data Availability Section)
State if relevant guidelines or checklists (e.g., **ICMJE, MIBBI, ARRIVE, PRISMA**) have been followed or provided.	Not Applicable	
For **tumor marker prognostic studies,** we recommend that you follow the **REMARK** reporting guidelines (see link list at top right). See author guidelines, under 'Reporting Guidelines'. Please confirm you have followed these guidelines.	Not Applicable	
For **phase II and III randomized controlled trials**, please refer to the **CONSORT** flow diagram (see link list at top right) and submit the CONSORT checklist (see link list at top right) with your submission. See author guidelines, under 'Reporting Guidelines'. Please confirm you have submitted this list.	Not Applicable	

Data Availability

Data availability	Information included in the manuscript?	In which section is the information available? (Reagents and Tools Table, Materials and Methods, Figures, Data Availability Section)
Have **primary datasets** been deposited according to the journal's guidelines (see 'Data Deposition' section) and the respective accession numbers provided in the Data Availability Section?	Yes	Data Availability Section
Were **human clinical and genomic datasets** deposited in a public access-controlled repository in accordance to ethical obligations to the patients and to the applicable consent agreement?	Not Applicable	
Are **computational models** that are central and integral to a study available without restrictions in a machine-readable form? Were the relevant accession numbers or links provided?	Not Applicable	
If publicly available data were reused, provide the respective **data citations in the reference list**.	Not Applicable	
