# Supplementary material for: UHRF1 overexpression generates distinct senescent states with different Tp53 dependencies
Source: EMBO Rep. 2026 Sep 28;27(21):6425–46. doi: 10.1038/s44319-026-00941-y (PMC13743750; doi:10.1038/s44319-026-00941-y)

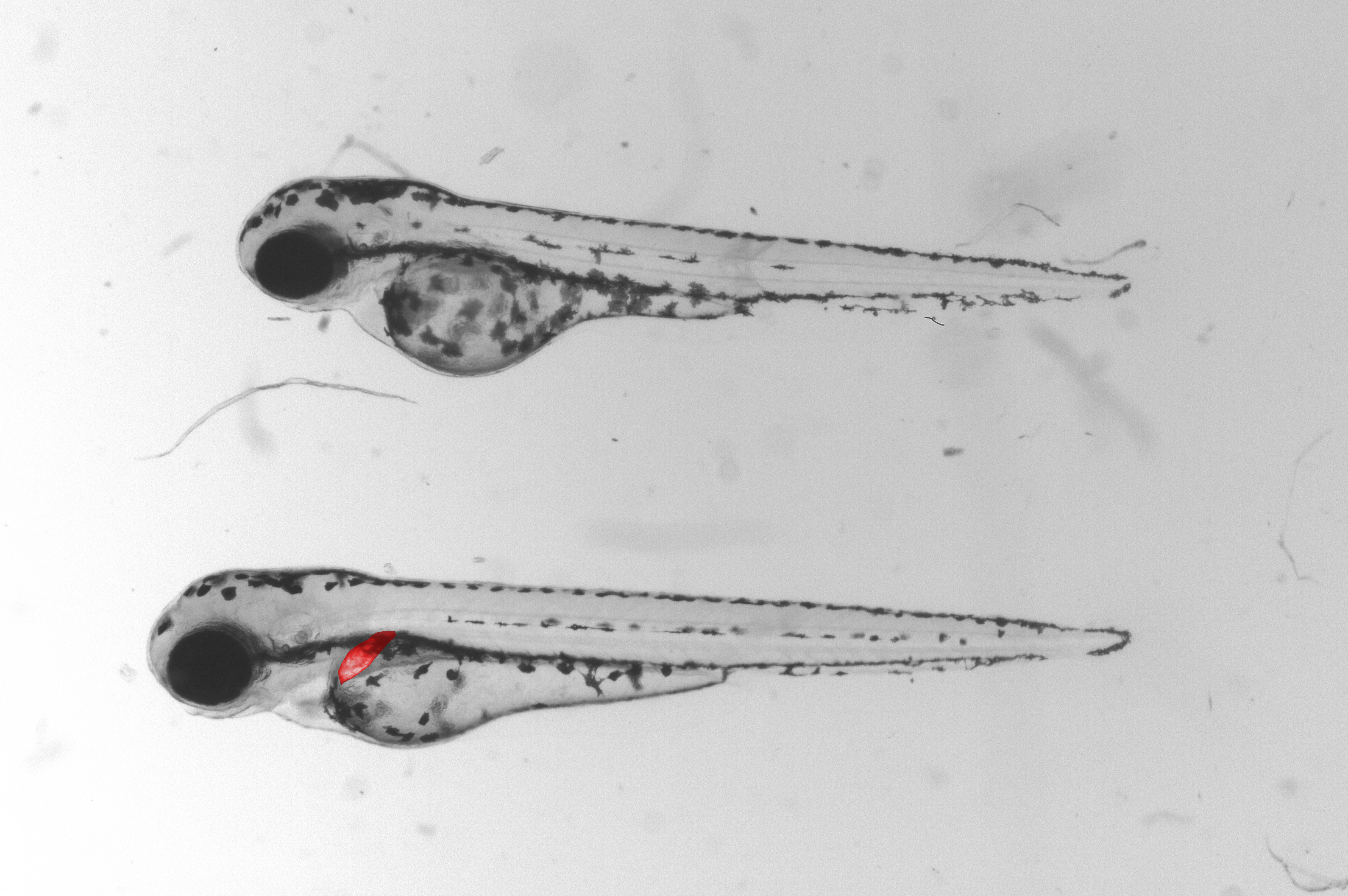

Supplement: Supplementary file 7 [file 44319_2026_941_MOESM8_ESM.zip › Fig1/Fig1A/Cherry_72h.pdf]
